# Spatiotemporal dynamics of HIV-1 transmission networks in a major migration hub: integrated phylogenetic and molecular evidence

**DOI:** 10.3389/fmicb.2025.1682213

**Published:** 2025-11-13

**Authors:** Min Zhu, Junfang Chen, Zhou Sun, Ke Xu, Xingliang Zhang, Sisheng Wu, Ling Ye, Xiaojuan Xu, Wenjie Luo

**Affiliations:** 1 Department of HIV/AIDS Control and Prevention, Hangzhou Center for Disease Control and Prevention (Hangzhou Health Supervision Institution), Hangzhou, China; 2 Zhejiang Key Laboratory of Multi-Omics in Infection and Immunity, Hangzhou, China; 3 Jiande Hospital of Traditional Chinese Medicine, Hangzhou, China

**Keywords:** HIV-1 transmission networks, population mobility, cross-regional connection, molecular epidemiology, Bayesian analysis

## Abstract

**Objective:**

Human Immunodeficiency Virus type 1 (HIV-1) cross-regional transmission poses a critical challenge in China, particularly in high-mobility metropolitan centers. This study aimed to characterize the transmission dynamics between Hangzhou—a megacity with 11.9 million residents (42% migrants)—and other Chinese regions using molecular epidemiology.

**Methods:**

We analyzed 4,249 Hangzhou-derived and 50,898 non-Hangzhou HIV-1 pol sequences. Molecular transmission network analysis was used to identify transmission clusters, and phylogenetic and Bayesian analyses were conducted to explore lineage characteristics, origins, and expansion of major clusters.

**Results:**

Molecular transmission network analysis identified 3,317 clusters, incorporating 43.5% (1,848/4,249) of Hangzhou sequences and 32.4% (16,511/50,898) of non-Hangzhou sequences. Crucially, 276 mixed-origin clusters bridged regions, comprising 1,222 (28.8%) Hangzhou and 8,954 (17.6%) non-Hangzhou individuals. Cross-regional connectivity was dominated by Shenzhen (48.1% of 46,962 edges), followed by Beijing (16.5%) and Guangzhou (7.9%). Multivariable regression revealed significantly higher odds of cross-regional connection for males versus females (aOR = 1.376, CI: 1.011–1.869, *p* = 0.043), homosexual transmission (aOR = 1.28, CI: 1.057–1.550, *p* = 0.009), non-residents (aOR = 1.207, CI: 1.040–1.402, *p* = 0.014), and first CD4 + T-cell count of 200–500 cells/uL (aOR = 1.348, CI: 1.057–1.718, *p* = 0.016). For subtypes, CRF07_BC and URF (CRF07_BC/CRF01_AE) demonstrated significant cross-regional spread versus other subtypes (aOR = 0.163–0.508, *p* < 0.001). Phylogenetic analysis of all Hangzhou CRF07_BC sequences identified two distinct lineages. Within the largest transmission CRF07_BC cluster, 99.5% of cross-regionally linked Hangzhou sequences (558/561) belonged to Lineage 1 indicating lineage 1 driving cross-regional spread. Bayesian dating indicated major URF clusters (HZC1-3, NHZ) originated between 2014 and 2020 (evolutionary rate: 1.73 × 10^−3^ subs/site/year).

**Conclusion:**

These findings identify key transmission routes connecting Hangzhou to economically developed regions and highlight CRF07_BC/URF strains and mobility as critical drivers. Targeted interventions disrupting these high-risk pathways are urgently needed to reduce regional HIV spread.

## Introduction

Human Immunodeficiency Virus type 1 (HIV-1) remains a significant global public health challenge, with approximately 40.8 million people living with HIV worldwide and 1.3 million new infections reported in 2024 alone (UNAIDS, 2024). By the end of 2020, China had an estimated 1.1 million people living with HIV (PLWH) and 351,000 cumulative reported deaths (He, 2021). A critical challenge within China’s densely populated landscape is the rapidly increasing HIV-1 prevalence among its substantial floating population (Su et al., 2018). Understanding the intricate patterns of HIV-1 transmission, particularly cross-regional spread, is therefore significant for designing effective, targeted prevention strategies to control the epidemic.

Hangzhou, the capital of economically dynamic Zhejiang Province and a central hub in the Yangtze River Delta, exemplifies this challenge. By 2020, its population reached 11.9 million long-term residents (Hangzhou Bureau of Statistics, 2023), including a floating population of 5.0 million, accounting for 42.0% of residents (Zhejiang Bureau of Statistics, 2022). Molecular epidemiological studies further demonstrate that these demographic conditions have established Hangzhou as a critical nexus for HIV transmission, with research showing the city accounts for 72% of local and 62% of cross-regional transmission within Zhejiang Province (Zhang et al., 2017). This central role in regional transmission networks, extending beyond its demographic significance, highlights Hangzhou’s importance in understanding and addressing HIV spread in the Yangtze River Delta region.

Molecular transmission network analysis has emerged as a powerful tool for elucidating transmission dynamics. By identifying phylogenetic clusters, researchers can identify actively growing transmission chains, geographical hotspots, and connections spanning different regions or populations (Rhee et al., 2019; Jiang et al., 2022). While previous studies in China have utilized these networks to characterize national patterns, provincial-level dynamics, and transmission within specific subtypes like CRF07_BC and CRF01_AE (An et al., 2020; Ge et al., 2021), analyses focusing on the interplay between localized transmission clusters and cross-regional virus importation/exportation within major metropolitan centers like Hangzhou remain relatively scarce.

To address this gap, we conducted a large-scale molecular transmission network analysis integrated with Bayesian evolutionary dating, centered on the Hangzhou metropolitan area. We assessed the extent, geographic linkages, and critical risk factors underpinning cross-regional spread and traced the origins and expansion of major transmission clusters. By demonstrating how this integrated molecular analysis can identify the most significant external connections and internal drivers for a given city, this study provides a replicable model for other Chinese metropolitan areas. Ultimately, characterizing these transmission patterns and drivers enables the design of targeted interventions to disrupt regional transmission networks.

## Materials and methods

### Study population and sequences collection

In this study, we analyzed 4,249 HIV-1 polymerase (pol) sequences derived from newly diagnosed cases in Hangzhou between 2019 and 2023. These sequences were generated in-house and had undergone complete quality control during the sequencing process. Whole blood samples were collected alongside comprehensive socio-demographic and clinical data, including sex, age, ethnicity, education level, occasion, current residence, marital status, transmission route, and high-risk sexual behaviors. All participants signed informed consent and the study was approved by the Medical Ethics Committee of the Hangzhou Municipal Center for Disease Control and Prevention.

**Figure 1:**
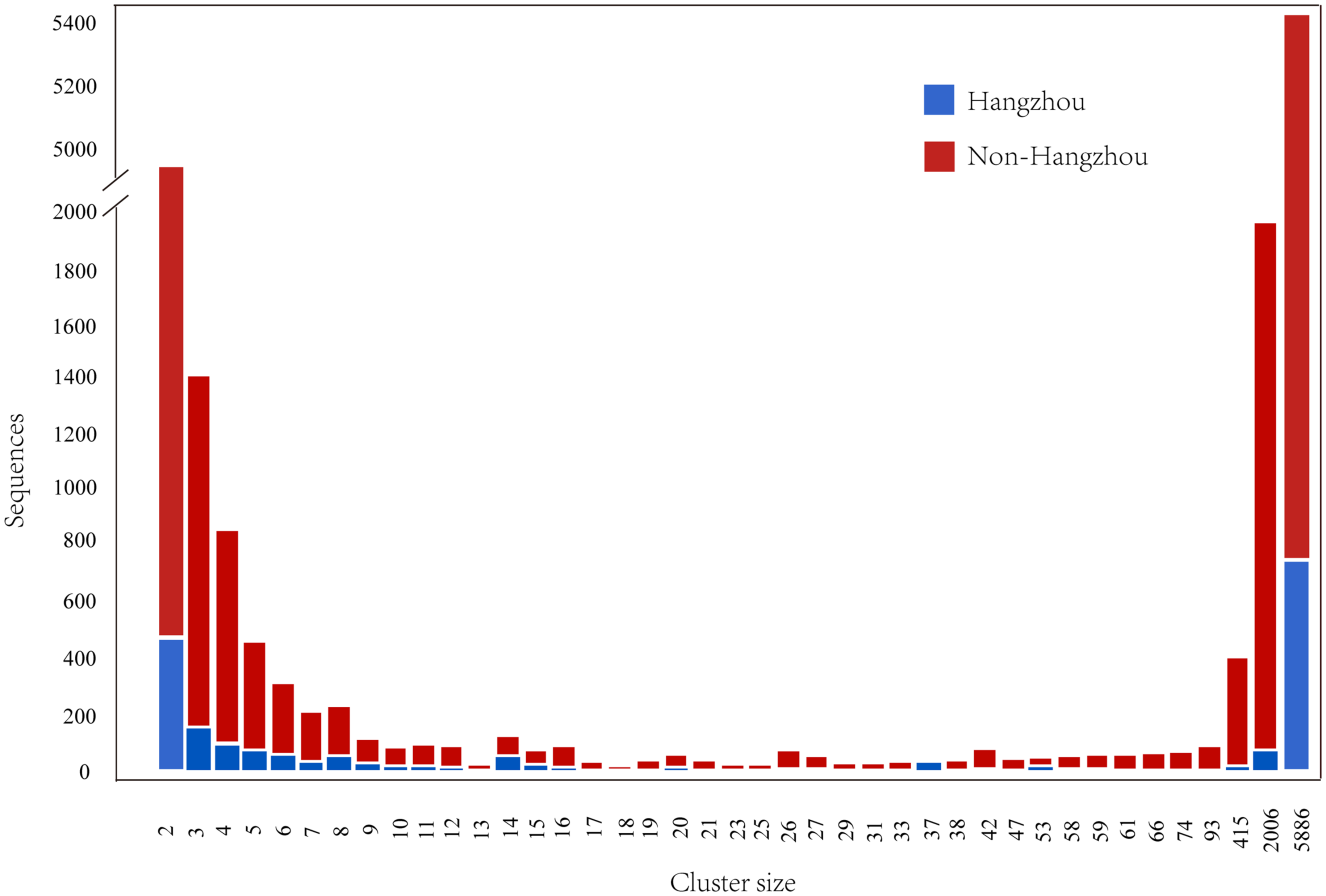
Composition of HIV-1 molecular transmission clusters by size. The plot shows the total number of Hangzhou (blue) and non-Hangzhou (red) sequences within all clusters of each size (ranging from 2 to 5,886 individuals).

For comparative background data, we searched the Los Alamos National Laboratories (LANL) HIV Sequence Database, which contains all published HIV-1 sequences. Figure 1 details the subsequent quality filtering process applied to these downloaded LANL sequences. The LANL HIV Sequence Database, generated January 16, 2024, included 59,022 HIV-1 sequences from China. These sequences underwent comprehensive quality filtering: sequences were required to cover HXB2 reference positions 2,253–3,554 (K03455), maintain ≥1,000 nucleotides in length, contain <5% ambiguous bases, and have documented sampling locations. We excluded sequences annotated as “problematic” in LANL. During the quality control process, we additionally excluded any sequences whose sampling location was documented as “Hangzhou” to avoid overlap with our local cohort. After this filtering, 50,898 non-Hangzhou sequences from China met the inclusion criteria for subsequent analysis. The flowchart is as follows.
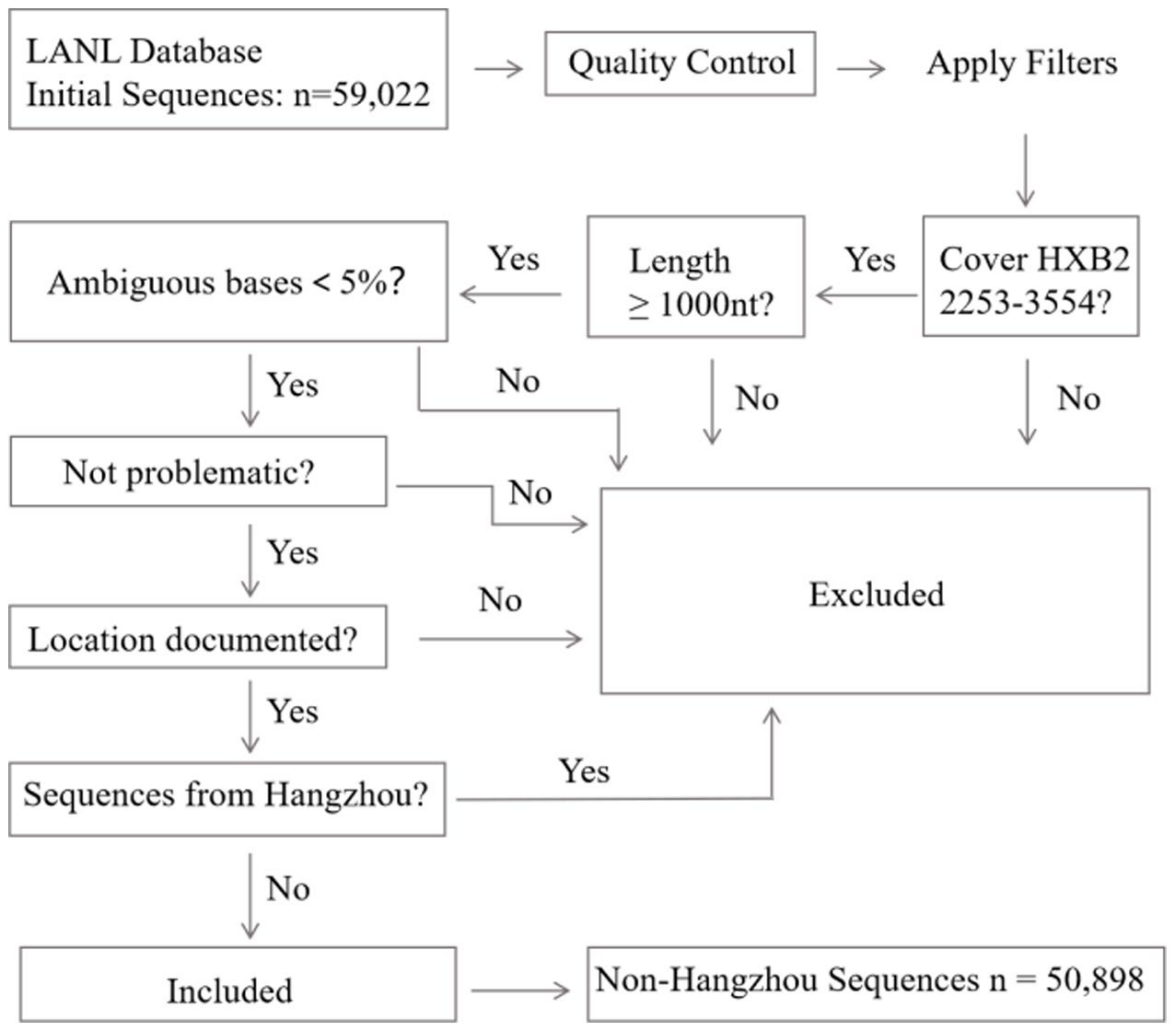


### Transmission network analysis

The Hangzhou sequences were aligned with HIV reference sequences downloaded from the LANL HIV Sequence Database by using MAFFT v7.037, followed by manual inspection and refinement using BioEdit 7.0.5.3. A total of 4,249 Hangzhou sequences and 50,898 non-Hangzhou reference sequences were included in this analysis. Subtypes were identified by using a neighbor-joining (NJ) phylogenetic tree constructed by using MEGA v11.0.13 based on the Kimura 2-parameter model. HIV-1 subtypes were determined by comparing the query sequences with reference sequences from the LANL database. The sequence was assigned to a particular subtype if it clustered within a monophyletic clade with the corresponding reference sequences with a bootstrap value ≥75%. The HIV-1 molecular transmission network was established using HIV-TRACE algorithm (Kosakovsky Pond et al., 2018). The pairwise genetic distances of sequences were calculated by the TN93 model using HyPhy (Pond et al., 2005). We implemented a 2-tiered TN93 distance cutoff to define a link (edge) between 2 sequences (nodes). The selection of these specific thresholds was guided by our study objective to identify recent transmission events for public health intervention. A threshold of 0.5% was applied for linkages exclusively within Hangzhou sequences or exclusively within non-Hangzhou sequences. This cutoff is consistent with recommendations for identifying transmission pairs within approximately 5 years, as it balances sensitivity for recent links with specificity against spurious connections arising from background genetic diversity (CDC, 2018). For cross-regional linkages between Hangzhou and non-Hangzhou sequences, a threshold of 1.0% was used. This slightly more relaxed criterion accounts for potential greater genetic variation and differences in sampling time across diverse geographic regions, a well-established strategy in the field (Rhee et al., 2019).

Using the above criteria, a molecular transmission network was reconstructed. Clusters were defined as groups of two or more sequences linked by genetic distance cutoffs. This analysis identified 3,512 clusters in total. Overall, 1,848 individuals from Hangzhou and 16,511 individuals from non-Hangzhou regions were linked into the network.

In the molecular network of HIV cross-regional transmission, connections between sequences from Hangzhou were defined as intra-city connections, while connections between Hangzhou sequences and those from non-Hangzhou regions were defined as cross-regional connections.

### Phylogenetic analysis

To further investigate the evolutionary history and transmission dynamics of key clusters identified in the transmission network analysis, a detailed phylogenetic analysis was performed on several vital clusters. Maximum likelihood (ML) tree was constructed in IQ-TREE v2.2.2.6 using the best-fitting nucleotide substitution model GTR + G + I. The ML tree was visually edited in FigTree v1.4.4.

BEAST v.1.10.4 under an uncorrelated relaxed clock model, GTR + G + I nucleotide substitution model, and Bayesian skyline plot demographic model were used to perform Bayesian evolutionary analysis (Suchard et al., 2018). BEAST analysis was performed using Markov Chain Monte Carlo (MCMC) runs of 100 million generations and sampled every 10,000 steps. The Bayesian MCMC output was analyzed using Tracer v1.7.2 (Rambaut et al., 2018). Maximum clade credibility (MCC) trees were generated using the TreeAnnotator v1.10.4 and visually edited in FigTree v1.4.4.

### Statistical analysis

A logistic regression model was used to analyze factors influencing the formation of cross-regional connections. Chi-square test, Fisher’s exact test and two-way ANOVA were performed in Graphpad Prism 9, Turkey multiple comparisons test was performed after two-way ANOVA. The Chi-square test was employed as a univariable regression. Multivariable logistic regression was performed in SPSS 25. The adjusted odds ratios (aOR) and 95% confidence intervals (CI) were calculated.

## Results

### Demographic characteristics of the study population in Hangzhou

Among 5,201 plasma samples from individuals newly diagnosed with HIV in Hangzhou (2019–2023), viral sequences were successfully obtained for 4,249 participants (81.7%). Comparison of available demographic and clinical characteristics between the successfully sequenced individuals and those for which sequencing failed showed no significant differences (Supplementary Table S1), indicating that the sequenced cohort was representative of the overall population. Demographically, the majority of sequenced cases (*N* = 4,249) were male (90.3%, 3,838/4,249), under 50 years old (78.0%, 3,314/4,249), and of Han ethnicity (95.6%, 4,064/4,249). Over half were current residents of Hangzhou (65.2%, 2,771/4,249), had attained at least senior high school education (60.3%, 2,562/4,249), and were unmarried (56.3%, 2,394/4,249). Clinically, 52.8% (2,243/4,249) presented with moderate immunosuppression (CD4 + T cell count: 200–500 cells/uL). The predominant HIV-1 acquisition risk factors were men who have sex with men (MSM, 64.6%, 2,743/4,249) and heterosexual contact (32.7%, 1,393/4,249), with minimal contributions from mother-to-child transmission (<0.1%; 2/4,249) or unknown routes (2.6%; 111/4,249).

The predominant subtype was CRF07_BC (45.0%, 1,913/4,249), followed by CRF01_AE (35.4%, 1,504/4,249), CRF08_BC (4.9%, 210/4,249), CRF55_01B (4.1%, 176/4,249), URF CRF07_BC/CRF01_AE (3.2%, 136/4,249), and B (2.5%, 108/4,249) (Table 1).

**Table 1:** Demographic characteristics of the study population in Hangzhou.

Variable	Newly reported HIV infections	% (*N* = 4,249)
Sex		
Male	3,838	90.3
Female	411	9.7
Ethnicity		
Han	4,064	95.6
Others	185	4.4
Age		
≥50 years old	935	22.0
25–50 years old	2,177	51.2
≤25 years old	1,137	26.8
Current place of residence		
Hangzhou	2,771	65.2
Outside Hangzhou city	1,478	34.8
Education		
Illiterate	95	2.2
Primary school	541	12.8
Junior high school	1,051	24.7
Senior high school and over	2,562	60.3
Occasion		
Commercial and public service workers	1,495	35.2
Workers and farmers	1,234	29.0
Domestic workers and unemployed	589	13.9
Staff of enterprises and public institutions	404	9.5
Students	184	4.3
Others	343	8.1
Marital status		
Unmarried	2,394	56.3
Married	1,120	26.4
Divorce or widowed	698	16.4
Unknown	37	0.9
Infection route		
Homosexual route	2,743	64.6
Heterosexual route	1,393	32.8
Others and unknown	113	2.7
Sample source		
Clinical samples	2,197	51.7
VCT	937	22.1
STD clinic	698	16.4
Sexual partner testing	43	1.0
Others	374	8.8
CD4 count before ART (cells/μL)		
≤200	1,582	37.2
200–500	2,243	52.8
≥500	424	10.0
Subtype		
CRF07_BC	1913	45.0
CRF01_AE	1,504	35.4
CRF08_BC	210	4.9
CRF55_01B	176	4.1
B	108	2.5
URF (CRF07_BC/CRF01_AE)	136	3.2
Others	202	4.8

### HIV molecular transmission network analysis

Molecular transmission network analysis identified 3,317 clusters ranging in size from 2 to 5,886 individuals. A total of 1,848 individuals from Hangzhou (43.5% of 4,249 Hangzhou sequences) and 16,510 individuals from non-Hangzhou regions (32.4% of 50,898 non-Hangzhou sequences) were incorporated into these clusters. Among these, 200 clusters contained exclusively Hangzhou individuals, comprising 555 individuals (13.1% of all Hangzhou sequences), while 276 clusters included both Hangzhou and non-Hangzhou individuals. The mixed-origin clusters contained 1,222 Hangzhou individuals (28.8% of all Hangzhou sequences) and 8,954 non-Hangzhou individuals (17.6% of all non-Hangzhou sequences). The size distribution followed a characteristic power-law pattern, with a majority of small clusters and a few exceptionally large clusters. Small clusters (2–16 individuals) predominated, accounting for 98.9% of all clusters (3,282/3,317). Among these, pairs (size 2) were the most frequent, representing 70.0% of all clusters (2,312/3,317). The frequency of clusters decreased rapidly with increasing size, with only 35 clusters (1.1%) containing ≥17 individuals. Notably, the network was dominated by three massive clusters containing 5,886, 2,006, and 415 individuals, respectively. These three largest clusters alone accounted for 15.1% (8,307/55,147) of all sequences incorporated into the network. In these largest clusters, non-Hangzhou individuals substantially outnumbered Hangzhou individuals, representing 90.5% (5,325/5,886), 95.9% (1,923/2,006), and 95.9% (398/415) of the cluster compositions, respectively.

Among the 35 clusters containing ≥17 individuals, 28 were of mixed origin (containing both Hangzhou and non-Hangzhou sequences). In 27 of these 28 mixed large clusters (96.4%), non-Hangzhou individuals numerically predominated, representing over 50% of the cluster membership. This pattern suggests extensive cross-regional transmission with non-Hangzhou sources playing a major role in the transmission and sustaining large transmission clusters in Hangzhou (Figure 1).

**Figure 2:**
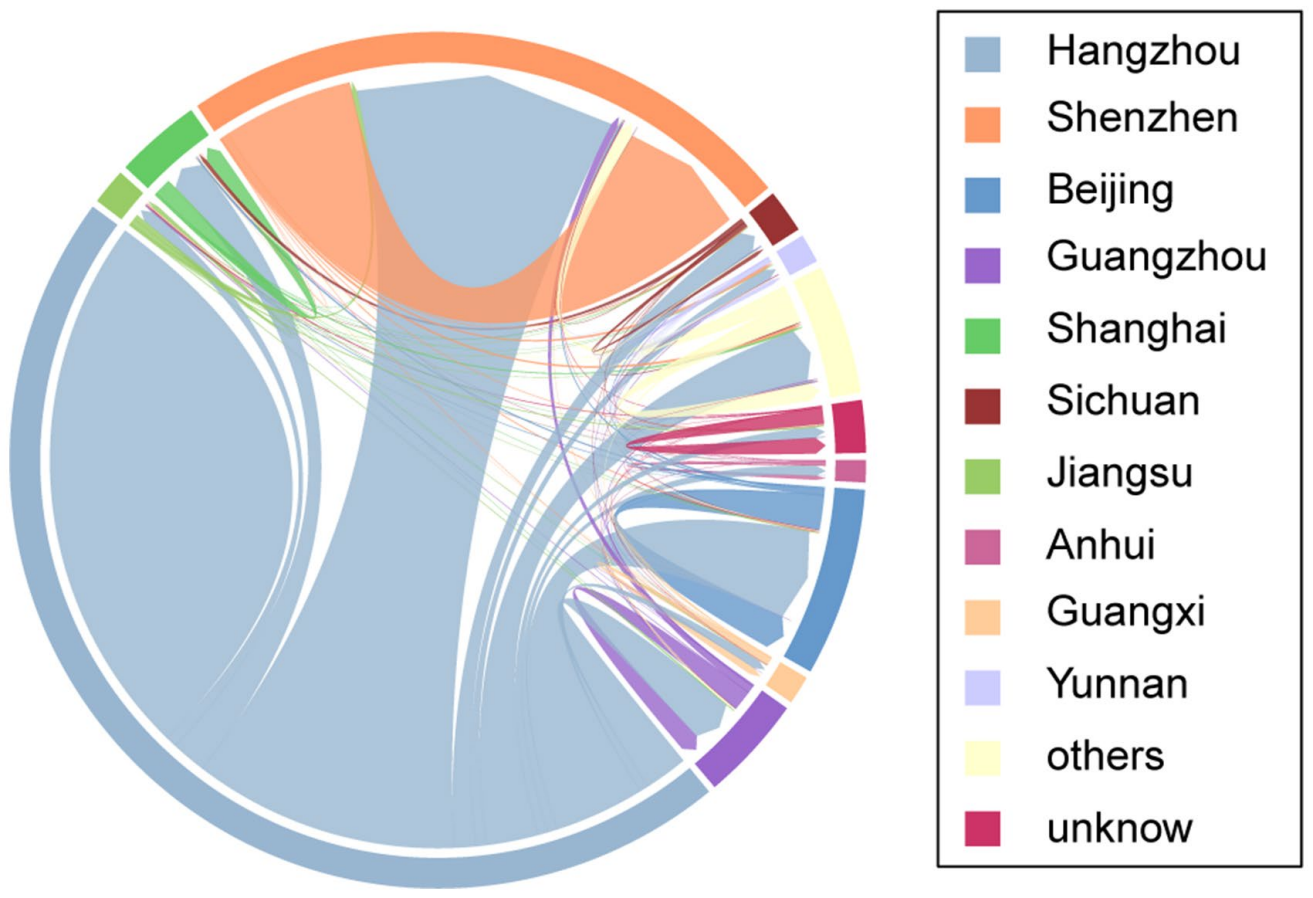
Connection patterns between Hangzhou and Non-Hangzhou sequences, categorized by different regions in China. Sequences connected to multiple regions are represented once for each connection.

Within the 276 mixed-origin clusters, 1,119 (91.6%) of the 1,222 Hangzhou individuals were directly linked to at least one non-Hangzhou individual. These connections formed 46,962 network edges. The strongest cross-regional linkages occurred with Shenzhen, accounting for 48.1% (22,570/46,962) of these edges, followed by Beijing (7,764/46,962; 16.5%), Guangzhou (3,729/46,962; 7.9%), Shanghai (2,984/46,962; 6.4%), Sichuan (1,891/46,962; 3.9%), Jiangsu (1,021/46,962; 2.2%), Yunnan (894/46,962; 1.9%), Anhui (839/46,962; 1.8%), and Guangxi (766/46,962; 1.6%). To account for the uneven sampling across regions, we also calculated the proportion of cross-regionally linked Hangzhou sequences that were connected to each major city. This normalized metric confirmed Shenzhen’s predominant role: among the 1,222 Hangzhou sequences in mixed-origin clusters, 45.2% (552/1222) were linked to sequences from Shenzhen, significantly higher than the proportions for Beijing (22.1%, 270/1222) and Guangzhou (11.5%, 141/1222). Figure 2 illustrates the complex connectivity patterns between Hangzhou and non-Hangzhou individuals.

### Influencing factors of cross-regional connection

Univariable logistic regression identified sex, age, education level, sampling occasion, marital status, infection route, first CD4 count prior to ART initiation, and viral subtype as factors potentially associated with cross-regional connections between Hangzhou and non-Hangzhou sequences in the transmission network. All significant variables from the univariable analysis were subsequently included in a multivariable logistic regression model (Table 2).

**Table 2:** Influencing factors of cross-regional connection.

Variable	Cross-regional connection in the transmission network		Univariable analysis		Multivariable logistic regression	
	Yes	Not	c^2	*P*-value	aOR (95% CI)	*P*-value
Sex			30.1	**<0.001**		
Female	73	338			1	
Male	1,180	2,658			1.376 (1.011–1.869)	**0.043**
Age			27.35	**<0.001**		
25–50 years old	651	1,526			1	
≤25 years old	384	753			1.052 (0.877–1.261)	0.585
≥50 years old	218	717			0.975 (0.781–1.218)	0.827
Current place of residence			1.47	0.2258		
Hangzhou	800	1971			1	
Outside Hangzhou city	453	1,025			1.207 (1.04–1.402)	**0.014**
Sampling site			3.3	0.0692		
Main urban area	1,051	2,443			1	
Non-main urban area	202	553			0.975 (0.8–1.187)	0.8
Education			28.18	**<0.001**		
Senior high school and over	808	1754			1	
Junior high school	312	739			1.1 (0.918–1.318)	0.304
Primary school	116	425			0.861 (0.655–1.133)	0.286
Illiterate	17	78			0.818 (0.456–1.466)	0.499
Occasion			15.84	**0.0073**		
Commercial and public service workers	467	1,028			1	
Workers and farmers	313	921			0.879 (0.72–1.073)	0.204
Domestic workers and unemployed	187	402			1.161 (0.931–1.447)	0.185
Staff of enterprises and public institutions	117	287			0.966 (0.748–1.246)	0.787
Students	62	122			0.944 (0.666–1.337)	0.744
Others	107	236			1.086 (0.835–1.412)	0.54
Marital status			32.43	**<0.001**		
Unmarried	786	1,608			1	
Married	278	842			0.926 (0.747–1.149)	0.486
Divorce or widowed	184	514			0.894 (0.709–1.126)	0.341
Unknown	5	32			0.4 (0.151–1.06)	0.065
Infection route			64.76	**<0.001**		
Heterosexual route	308	1,085			1	
Homosexual route	923	1820			1.280 (1.057–1.550)	**0.012**
Others and unknown	22	91			0.63 (0.384–1.032)	0.066
Sample source			4.23	0.3753		
Clinical samples	620	1,577				
VCT	292	645				
STD clinic	217	481				
Sexual partner testing	11	32				
Others	113	261				
CD4 count before ART (cells/μL)			8.45	**0.0146**		
≥500	107	317			1	
≤200	445	1,137			1.094 (0.938–1.277)	0.251
200–500	701	1,542			1.348 (1.057–1.718)	**0.016**
Subtype			171	**<0.001**		
CRF07_BC	715	1,198			1	
CRF01_AE	345	1,159			0.49 (0.421–0.572)	**<0.001**
CRF08_BC	20	190			0.237 (0.146–0.385)	**<0.001**
CRF55_01B	67	109			0.965 (0.7–1.331)	0.829
B	8	100			0.163 (0.078–0.339)	**<0.001**
URF (CRF07_BC/CRF01_AE)	53	83			0.496 (0.35–0.703)	**<0.001**
Others	45	157			0.965 (0.673–1.384)	0.848
Subtype (*)						
URF (CRF07_BC/CRF01_AE)	53	83			1	
CRF07_BC	715	1,198			1.036 (0.722–1.486)	0.848
CRF01_AE	345	1,159			0.508 (0.352–0.734)	**<0.001**
CRF08_BC	20	190			0.246 (0.136–0.446)	**<0.001**
CRF55_01B	67	109			1 (0.629–1.589)	1
B	8	100			0.169 (0.075–0.379)	**<0.001**
Others	45	157			0.514 (0.316–0.834)	0.007

aOR, adjusted odds ratios; CI, confidence intervals.

*Change the URF (CRF07_BC/CRF01_AE) as the reference subtype.

Males demonstrated significantly higher odds of cross-regional connection compared to females (aOR = 1.376, 95% CI: 1.011, 1.869, *p* = 0.043). Individuals infected via homosexual contact had significantly higher odds of connection compared to those infected heterosexually (aOR = 1.280, 95% CI: 1.057–1.550, *p* = 0.012). Non-Hangzhou residents exhibited 1.207 times higher odds of connection than Hangzhou residents (95% CI: 1.040–1.402, *p* = 0.014). Individuals with a first CD4 count of 200–500 cells/μL before ART had significantly higher odds compared to those with CD4 counts >500 cells/μL (aOR = 1.348, 95% CI: 1.057–1.718, *p* = 0.016).

For subtype, using CRF07_BC as the reference subtype, significantly lower odds of connection were observed for CRF01_AE (aOR = 0.49, 95% CI: 0.421–0.572, *p* < 0.001), CRF08_BC (aOR = 0.237, 95% CI: 0.146–0.385, p < 0.001), subtype B (aOR = 0.163, 95% CI: 0.078–0.339, *p* < 0.001), and URF (CRF07_BC/CRF01_AE) (aOR = 0.496, 95% CI: 0.35–0.703, *p* < 0.001). Notably, URF (CRF07_BC/CRF01_AE) showed significantly higher odds of cross-regional connection compared to CRF01_AE (aOR = 2.03, 95% CI: 1.362–3.025, *p* < 0.001), CRF08_BC (aOR = 4.11, 95% CI: 2.242–7.538, *p* < 0.001), and subtype B (aOR = 5.92, 95% CI: 2.639–13.289, *p* < 0.001).

### Phylogenetic analysis of notable clusters

**Figure 3:**
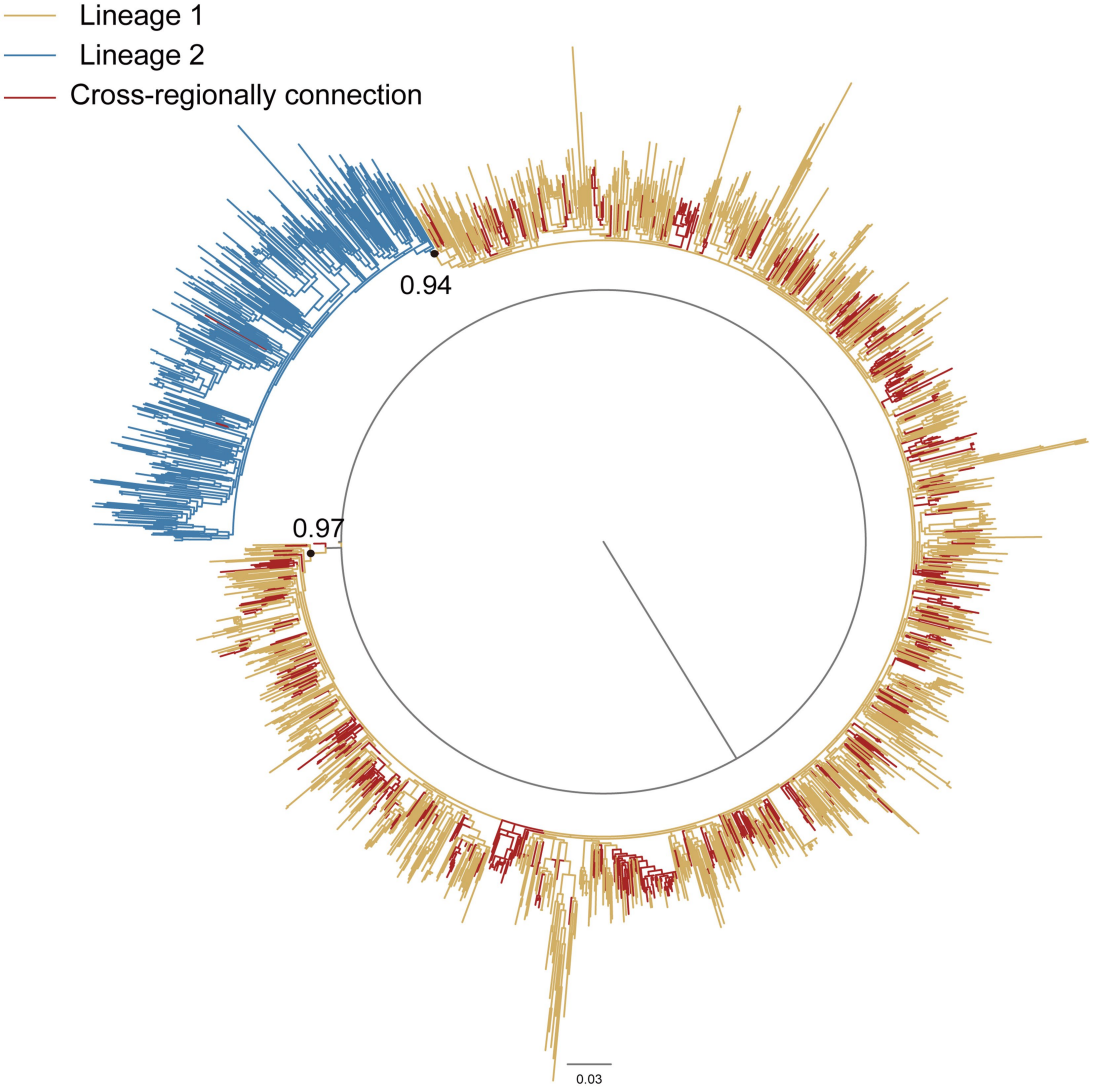
Maximum-likelihood phylogenetic tree of CRF07_BC. The nucleotide substitution mode was GTR + G + I. Blue branches represented the lineage 1. Yellow branches represented the lineage 2. Red branches represented Hangzhou sequences with cross-regional connections in the largest CRF07_BC cluster. Node support values (Bootstrap values) are shown for the two primary CRF07_BC lineages only, due to the large number of sequences.

Phylogenetic analysis was performed on notable transmission clusters identified within the network. The largest cluster (*n* = 5,886), predominantly comprising CRF07_BC sequences (561 from Hangzhou; 5,325 from non-Hangzhou regions), revealed significant geographical diversity. Non-Hangzhou sequences originated primarily from Shenzhen (2,317/5,325; 43.5%), Beijing (872/5,325; 16.4%), Guangzhou (579/5,325; 10.9%), and Shanghai (283/5,325; 5.3%). The earliest sequence isolated within this cluster was obtained in Yunnan in 2003. Six additional sequences were isolated in 2004 and 2005. These seven sequences formed 95 transmission links with sequences from Hangzhou, among which links to sequences from Beijing were the most frequent, accounting for 72.6% (69/95). Phylogenetic reconstruction identified two major distinct lineages (Lineage 1 and Lineage 2) of CRF07_BC circulating in Hangzhou (Figure 3). Notably, the 561 cross-regionally connected Hangzhou CRF07_BC sequences within this cluster were primarily distributed in Lineage 1 (99.4%, 558/561), suggesting this lineage was more strongly associated with cross-regional spread, while Lineage 2 appeared more locally focused within Hangzhou. Each lineage exhibited distinct epidemic characteristics, indicating complex HIV-1 transmission dynamics (Supplementary Table S2).

The second-largest cluster (*n* = 2,002), composed of CRF01_AE sequences (79 from Hangzhou; 1,923 non-Hangzhou), lacked similarly distinct major lineages upon phylogenetic analysis.

**Figure 4:**
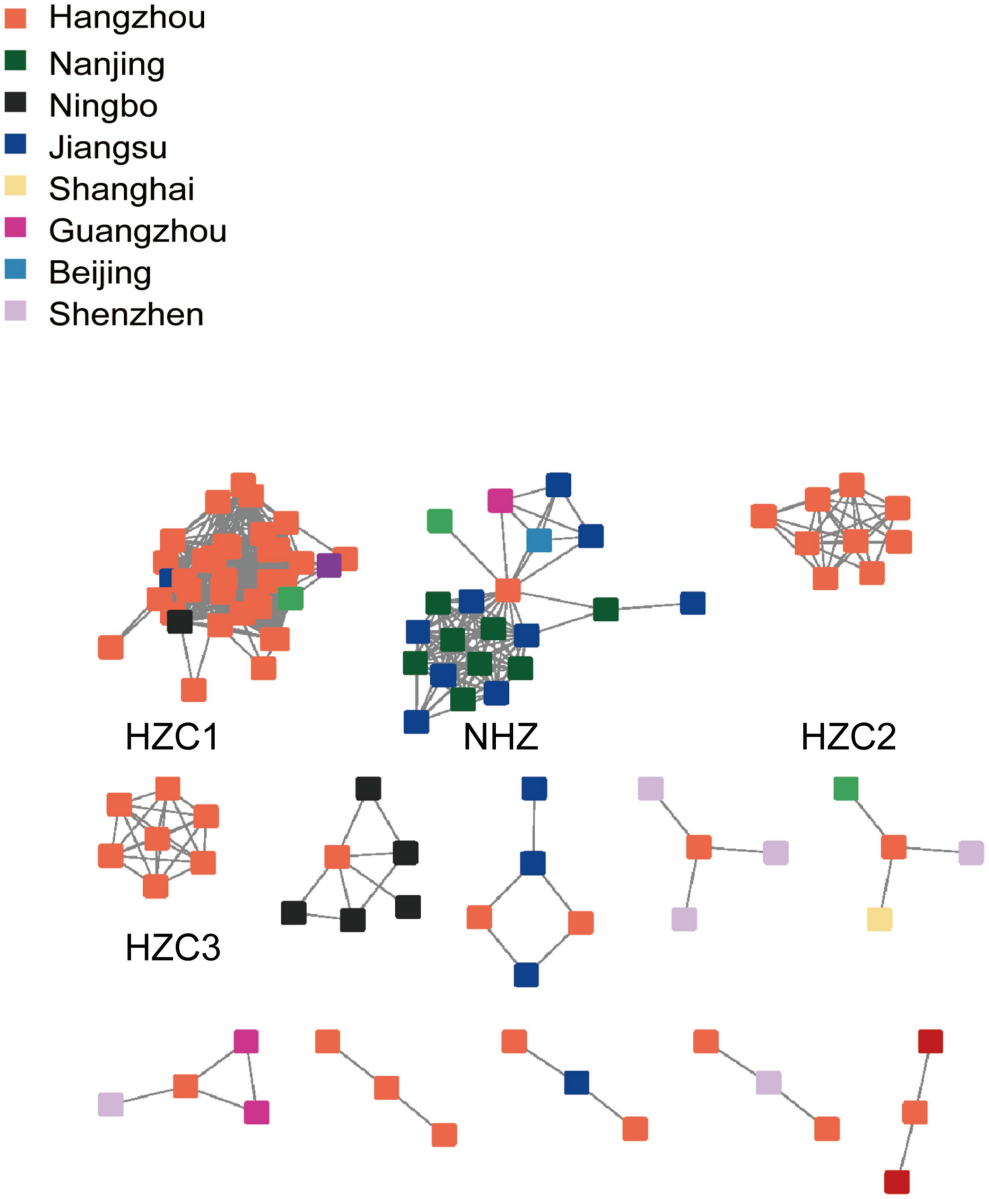
The molecular transmission network of Hangzhou sequences with cross-regional connections of URF (CRF07_BC/CRF01_AE) subtype. Only clusters with nodes ≥3 are shown in the figure. Colored nodes represent different regions.

Consistent with the multivariable logistic regression analysis identifying URF (CRF07_BC/CRF01_AE) as a significant factor for cross-regional connection, network analysis showed that 130 URF sequences formed 23 clusters (size range: 2–37). Among these, three large clusters (HZC1, HZC2, and HZC3) contained >7 Hangzhou individuals each, while another large cluster (NHZ) contained only one Hangzhou individual alongside 20 non-Hangzhou individuals (Figure 4).

To investigate the origins and spread of URF strains associated with Hangzhou’s major transmission clusters (HZC1, HZC2, HZC3, and NHZ), we estimated the time to the most recent common ancestor (tMRCA) using a Bayesian Markov chain Monte Carlo (MCMC) approach. We used TempEst v1.5.3 to test the molecular clock hypothesis and the result showed that the calculated R2 was 0.30. A relaxed molecular clock (log-normal) was applied under the GTR substitution model and a Bayesian skyline demographic model. The estimated evolutionary rate was 1.73 × 10^−3^ nucleotide substitutions/site/year (95% HPD: 1.39 × 10^−3^ – 2.10 × 10^−3^). The skyline plot indicated an exponential growth phase starting around 2016, stabilization between 2017 and 2018, a subsequent decline until 2021, followed by renewed stabilization.

**Figure 5:**
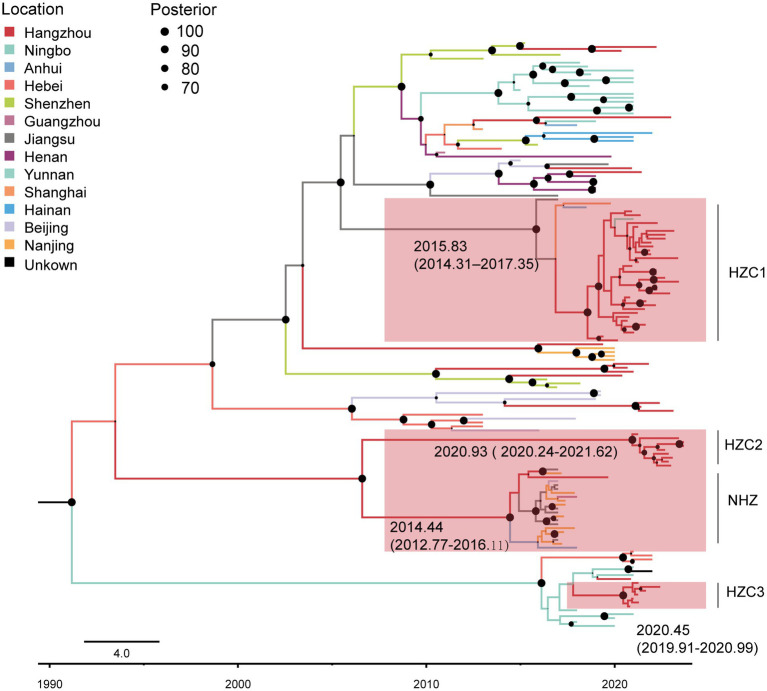
Maximum clade credibility (MCC) tree with the information of sample location and evolutionary time analyzed by BEAST v1.10.4 and constructed by FigTree v1.4.4. In the MCC tree, 130 URF sequences were included and 4 clusters HZC1-3, NHZ were highlighted. Different colors of branch represent different source locations of sequences. The branch lengths represent the evolutionary time, and nodes labeled with evolutionary time were mostly supported by a high posterior probability (≥80). The corresponding time scale was marked at the bottom of the MCC tree.

As shown in Figure 5, with the support of high posterior probability, the estimated tMRCAs of HZC1, HZC2, HZC3, and NHZ were 2015.83 (95% HPD interval 2014.31–2017.35), 2020.93 (95% HPD interval 2020.24–2021.62), 2020.45 (95% HPD interval 2019.91–2020.99), and 2014.44 (95% HPD interval 2012.77–2016.11), respectively.

Notably, in contrast to HZC1, HZC2, and HZC3 which primarily circulated within Hangzhou, phylogenetic reconstruction revealed that the NHZ lineage initially diverged through a Hangzhou sequence, followed by subsequent expansion to Jiangsu and Nanjing. This pattern is consistent with the central bridging position occupied by the Hangzhou sequence in the NHZ molecular cluster topology, suggesting its role in facilitating cross-regional transmission.

## Discussion

Our study demonstrates that Hangzhou acts as an important node in cross-regional transmission, with 29.4% of its clustered sequences forming connections beyond municipal boundaries. The disproportionate connectivity with Shenzhen, Beijing, and Guangzhou—China’s most economically developed cities—likely reflects extensive population mobility driven by economic activity. This pattern aligns with the elevated cross-regional risk observed among non-residents (aOR = 1.207) and individuals infected through homosexual contact (aOR = 1.280), suggesting that labor migration and key population mobility jointly drive viral dissemination. These two forms of migration likely shape the network through distinct mechanisms. Labor migration, represented by the non-resident population, may primarily facilitate the initial introduction and establishment of viral lineages into new locations through the movement of general populations. In contrast, mobility among MSM, a key population with high-risk sexual networks, appears to be a more potent driver of rapid cluster expansion and long-distance transmission, as evidenced by the dominance of the MSM-adapted CRF07_BC lineage in cross-regional clusters. These findings underscore the heterogeneous nature of ‘mobility’ as a risk factor, which correspond to the research that highlights mobility is not a monolithic process but encompasses diverse patterns—from economic migration to network-driven mobility among key populations (Deane et al., 2010). Furthermore, this dynamic is powerfully illustrated in Shenzhen, a key partner city in our network, where a study found that 90.3% of HIV-infected MSM were migrants, and these migrant MSM had significantly different HIV-1 subtype distributions compared to local residents (Zhao et al., 2016). This aligns with our observation of a massive transmission link between Hangzhou and Shenzhen, suggesting that the convergence of mass migration and MSM network mobility in Shenzhen creates a potent hub for viral amplification and redistribution, profoundly shaping the regional transmission network.

Notably, subtype-specific transmission patterns emerged as critical determinants. CRF07_BC dominated cross-regional spread, particularly through Lineage 1 which contained 99.4% of externally connected Hangzhou sequences in the largest cluster. CRF07_BC is the most prevalent strain circulating in China. According to the Chinese Center for Disease Control and Prevention (China CDC), CRF07_BC has undergone two distinct exponential growth phases, driven by the subclusters 07BC_O and 07BC_N, respectively. 07BC_O experienced significant expansion during the period 1991–2005, while 07BC_N exhibited rapid growth in the second phase following 2005. Although 07BC_N emerged later, it expanded rapidly after 2005, gradually superseding 07BC_O to become the dominant lineage. Furthermore, 07BC_N is predominantly circulating within the MSM population, leading to substantial increases in prevalence across central and eastern provinces (Gan et al., 2022; Wang et al., 2024). Critically, the overwhelming dominance of transmission links from Beijing (72.6%) observed among the earliest sequences within Hangzhou’s largest CRF07_BC cluster provides direct genetic evidence that Beijing served as a primary source for introducing and amplifying the 07BC_N subcluster into eastern China. This finding supports the north-to-east transmission dynamics driven by 07BC_N’s expansion within northern MSM networks, explaining its substantial contribution to the rising prevalence across eastern provinces.

The URF (CRF07_BC/CRF01_AE) subtype exhibited unexpectedly high dissemination potential, showing higher connection odds compared to other major subtypes despite its lower prevalence. Phylogenetic evidence further revealed heterogeneous transmission pathways: while clusters HZC1-HZC3 remained locally confined, the NHZ cluster originated from a Hangzhou sequence that occupied a central bridging position in the molecular topology, facilitating spread to Jiangsu and Nanjing with an estimated origin in 2014.44.

In contrast to prior studies focused either on single-city networks or national subtype dynamics (An et al., 2020; Ge et al., 2021; Gan et al., 2022; Li et al., 2023; Tan et al., 2023; Zhang et al., 2024), our analysis of Hangzhou within a national context provides a novel perspective. We move beyond describing local patterns to precisely quantify metropolitan-level connectivity, identify the specific viral lineages driving cross-regional spread, and link these findings to mobility, establishing a framework for targeting other key urban centers in China’s epidemic.

These findings necessitate targeted public health strategies. First, interventions should prioritize mobile populations moving between Hangzhou and high-GDP cities, particularly MSM and labor migrants. Second, enhanced surveillance of CRF07_BC and URF strains is warranted given their elevated cross-regional transmissibility. Finally, early identification of “bridge sequences” like the NHZ cluster could enable preventive disruption of emerging transmission networks.

This study had some limitations. First, reliance on the pol region alone may limit accurate identification of complex recombinant forms and obscure full-length genomic characteristics. Second, the results inferred from molecular networks constructed based on HIV evolutionary affinity may deviate from the real world.

## Conclusion

In conclusion, Hangzhou’s epidemic is characterized by complex connections to economically developed regions, driven by intersecting virological and demographic factors. Future strategies must adopt approaches that account for the unique dynamics of large urban centers and transcend administrative boundaries to disrupt transmission corridors.

## Data Availability

The datasets presented in this study can be found in online repositories. The names of the repository/repositories and accession number(s) can be found in the article/Supplementary material.
