# Supplementary material for: Spatiotemporal dynamics of HIV-1 transmission networks in a major migration hub: integrated phylogenetic and molecular evidence
Source: Front Microbiol. 2025 Nov 13;16:1682213. doi: 10.3389/fmicb.2025.1682213 (PMC12657489; doi:10.3389/fmicb.2025.1682213)
Supplement: Supplementary file 1 [file Data_Sheet_1.docx]

****Supplementary Table S1. Baseline Characteristics Comparison Between Sequenced and Non-sequenced Study Participants****

| Characteristic | Sequenced Group (N=4,249) (%) | Non-sequenced Group (N=952) (%) | P-value |
| --- | --- | --- | --- |
| **Sex** |  |  | 0.1446 |
| Male | 3838 (90.3) | 845 (88.8) |  |
| Female | 411 (9.7) | 107 (11.2) |  |
| **Ethnicity** |  |  | 0.9371 |
| Han | 4064 (95.6) | 910 (95.6) |  |
| Others | 185 (4.4) | 42 (4.4) |  |
| **Age** |  |  | 0.9138 |
| ≥ 50 years old | 935 (22.0) | 206 (21.6) |  |
| 25-50 years old | 2177 (51.2) | 495 (52.0) |  |
| ≤ 25 years old | 1137 (26.8) | 251 (26.4) |  |
| **Current place of residence** |  |  | 0.4178 |
| Hangzhou | 2771 (65.2) | 634 (66.6) |  |
| Outside Hangzhou city | 1478 (34.8) | 318 (33.4) |  |
| **Education** |  |  | 0.0657 |
| Illiterate | 95 (2.2) | 17 (1.8) |  |
| Primary school | 541 (12.8) | 114 (12.0) |  |
| Junior high school | 1051 (24.7) | 204 (21.4) |  |
| Senior high school and over | 2562 (60.3) | 617 (64.8) |  |
| **Marital status** |  |  | 0.0998 |
| Unmarried | 2394 (56.3) | 554 (58.2) |  |
| Married | 1120 (26.4) | 245 (25.7) |  |
| Divorce or widowed | 698 (16.4) | 138 (14.5) |  |
| Unknown | 37 (0.9) | 15 (1.6) |  |
| **Infection route** |  |  | 0.1156 |
| Homosexual route | 2743 (64.6) | 585 (61.4) |  |
| Heterosexual route | 1393 (32.8) | 345 (36.2) |  |
| Others and unknown | 113 (2.7) | 22 (2.3) |  |
| **CD4 count before ART (cells/µL** |  |  |  |
| ≤ 200 | 1582 (37.2) | 313 (32.9) | 0.0068 |
| 200-500 | 2243 (52.8) | 518 (54.4) |  |
| ≥ 500 | 424 (10.0) | 121 (12.7) |  |

****Supplementary Table S2.** Demographic characteristics of two lineages of CRF07_BC**

|  | CRF07_BC  Lineage1 （N=324） | CRF07_BC  Lineage2 （N=1586） |
| --- | --- | --- |
| **Sex** |  |  |
| Male | 237 (73.1%) | 1519 (95.8%) |
| Female | 87 (26.9%) | 67 (4.2%) |
| **Age** |  |  |
| ≥ 50 years old | 161 (49.7%) | 246 (15.5%) |
| 25-50 years old | 130 (40.1%) | 859 (54.2%) |
| ≤ 25 years old | 33 (10.2%) | 481 (30.3%) |
| **Current place of residence** |  |  |
| Hangzhou | 162 (50.0%) | 1092 (68.9%) |
| Outside Hangzhou city | 162 (50.0%) | 494 (31.1%) |
| **Education** |  |  |
| Illiterate | 25 (7.7%) | 14 (0.9%) |
| Primary school | 101 (31.2%) | 135 (8.5%) |
| Junior high school | 85 (26.2%) | 387 (24.4%) |
| Senior high school and over | 113 (34.9%) | 1050 (66.2%) |
| **Occasion** |  |  |
| Commercial and public service workers | 72 (22.2%) | 602 (38.0%) |
| Workers and farmers | 151 (46.6%) | 409 (25.8%) |
| Domestic workers and unemployed | 51 (15.7%) | 202 (12.7%) |
| Staff of enterprises and public institutions | 26 (8.0%) | 154 (9.7%) |
| Students | 5 (1.5%) | 85 (5.4%) |
| Others | 19 (5.9%) | 134 (8.4%) |
| **Marital status** |  |  |
| Unmarried | 93 (28.7%) | 986 (62.2%) |
| Married | 139 (42.9%) | 344 (21.7%) |
| Divorce or widowed | 88 (27.2%) | 244 (15.4%) |
| Unknown | 4 (1.2%) | 12 (0.8%) |
| **Infection route** |  |  |
| Homosexual route | 229 (70.7%) | 1251 (78.9%) |
| Heterosexual route | 79 (24.4%) | 306 (19.3%) |
| Others and unknown | 16 (4.9%) | 29 (1.8%) |
| **CD4 count before ART (Median and IQR, cells/μl)** | 223.5/243.8 | 280.0/206.8 |
| **Cross - regional connection with other cities** |  |  |
| Yes | 3 (0.9%) | 558 (35.2%) |
| No | 321 (99.1%) | 1028 (64.8%) |
