# Supplementary material for: Spatiotemporal dynamics of HIV-1 transmission networks in a major migration hub: integrated phylogenetic and molecular evidence
Source: Front Microbiol. 2025 Nov 13;16:1682213. doi: 10.3389/fmicb.2025.1682213 (PMC12657489; doi:10.3389/fmicb.2025.1682213)
Supplement: Supplementary file 2 [file Data_Sheet_2.docx]

Supplementary Table S3. Access number of sequences used for network analysis.

| MT986183 | MN800018 | MN427202 | MN797883 | MH300362 | KT378700 | KT379948 | KJ614200 | KJ401712 | OR521731 |
| --- | --- | --- | --- | --- | --- | --- | --- | --- | --- |
| MT986179 | MN799888 | MN427184 | MN797868 | MH300245 | KT379886 | KT379925 | KJ614162 | KJ401759 | OR521478 |
| MT986123 | MT589537 | MN427148 | MN797400 | MH300242 | KT378697 | KT379892 | KM258735 | KJ401725 | OR522447 |
| MT986112 | MN801515 | MN427138 | MN797385 | MH300241 | KT379680 | KT379834 | KJ614173 | KR188383 | OR522140 |
| MT986095 | MN803096 | MN427137 | MN798950 | MH300236 | KT379344 | KT379794 | KM395775 | KR188378 | OR521972 |
| MT986094 | MN802823 | MN427118 | MN797371 | MH300234 | KT378689 | KT379721 | KM395774 | KR188350 | OR521794 |
| MT986078 | MN802330 | MN427117 | MN798588 | MH300265 | KT378686 | KT379699 | KM395772 | KR188313 | OR521857 |
| MT590095 | MN802195 | MN427114 | MN797770 | MH300226 | KT378685 | KT379687 | KF267704 | KR188279 | OR521784 |
| MT986164 | MN801731 | MN427111 | MN797358 | MH632639 | KT379466 | KT379555 | KF267648 | KR188198 | MW957783 |
| MT589509 | MN800864 | MN427109 | MN797562 | MH300221 | KT379095 | KT379448 | KJ614187 | KR188178 | MW957831 |
| MT589494 | MT986081 | MN427099 | MN797350 | MH300382 | KT378683 | KT379423 | KJ614170 | KR188147 | MW957728 |
| MT589546 | MT590289 | MN427091 | MN798540 | MH300238 | KT378767 | KT379395 | KJ401672 | KR188143 | OR521709 |
| MT589467 | MT589422 | MN427070 | MN797345 | MH300215 | KT378680 | KT379320 | KJ401660 | KR188089 | OP831072 |
| MT589476 | MT588940 | MN427069 | MN799118 | MH300213 | KU050310 | KT379083 | KJ401491 | KR187957 | OP831039 |
| MT589091 | MT589485 | MN427067 | MN798964 | MH300210 | KT379594 | KT379015 | KJ401533 | KR187938 | OR522222 |
| MT589080 | MT589603 | MN427060 | MN798896 | MH300209 | KT378674 | KT378861 | KJ401462 | KR187923 | OP190436 |
| MT589829 | MT986157 | MN427058 | MN798652 | MH300211 | KT379950 | KT378839 | KF250366 | KR187870 | OR522321 |
| MT589054 | MN801875 | MN427054 | MN798395 | MH300206 | KT379890 | KT378658 | KP250792 | KR187774 | OR521790 |
| MT589497 | MN801327 | MN427053 | MN798355 | MH300373 | KT379697 | KT378655 | KM258724 | KR187719 | MW957957 |
| MT589037 | MN800660 | MN427051 | MN797872 | MH300203 | KT379254 | KR824017 | KM258837 | KR187647 | MW957685 |
| MT589004 | MT589102 | MN427050 | MN797763 | MH300287 | KT378879 | KR824003 | KM258741 | KR187602 | OR521375 |
| MT589352 | MN802578 | MN427049 | MN797522 | MH300201 | KT378721 | KR824001 | KJ401548 | KR187599 | OP830994 |
| MT588939 | MN802899 | MN427047 | MN797414 | MH300186 | KT378668 | KR823682 | KJ401416 | KR187548 | OP190702 |
| MT588996 | MN802763 | MN427046 | MN797323 | MH300185 | KT378667 | KR823673 | KJ401551 | KR187542 | OP831084 |
| MT588922 | MN801888 | MN427040 | MN797514 | MH300184 | KU050459 | KR823488 | KM258762 | KR187229 | OR522175 |
| MT589000 | MN801234 | MN427038 | MN797314 | MH300183 | KT378654 | KR823426 | KJ614034 | KR187213 | OK315249 |
| MT589712 | MN800351 | MN427034 | MN798693 | MH632605 | KT379858 | KR823328 | KM258707 | KC888563 | MZ956078 |
| MT588969 | MN802353 | MN427031 | MN798502 | MH220194 | KT379337 | KR823323 | KJ614151 | KC352112 | OR522003 |
| MT589294 | MN800712 | MN427030 | MN797396 | MH220193 | KT379009 | KR823304 | KM258733 | KR187997 | MW957956 |
| MT589107 | MN799932 | MN427021 | MN797291 | MH028347 | KT378958 | KR823302 | KM258781 | KR187815 | OP830982 |
| MT590139 | MT589396 | MN427020 | MN799719 | MH300156 | KT378903 | KR823183 | KP178439 | KR187595 | OR521719 |
| MT590023 | MN801710 | MN427019 | MN797938 | MH028330 | KT378758 | KR823175 | KM258731 | KR187363 | OP190551 |
| MT589964 | MN800046 | MN427016 | MN797290 | MH028343 | KT378650 | KR823162 | KP418604 | KR187289 | OP190547 |
| MT589425 | MN800475 | MN427013 | MN797286 | MH028325 | KT379771 | KR822965 | KP178424 | KR187219 | OK315023 |
| MT589426 | MN802214 | MN427012 | MN797279 | MH028344 | KT379018 | KR822952 | KJ570833 | KR187211 | OK315011 |
| MT589123 | MN802852 | MN427011 | MN797940 | MH028309 | KT378646 | KR823316 | KJ570790 | KR187206 | OP190909 |
| MT368379 | MN802320 | MN427008 | MN798786 | MH028302 | KT378643 | KR823207 | KM258804 | KC888436 | OP190461 |
| MT590036 | MT161835 | MN427006 | MN798761 | MH028300 | KT192026 | KT379739 | KM258786 | KR187498 | OR521711 |
| MT590031 | MN800848 | MN427005 | MN797313 | MH028299 | KT192025 | KT379304 | KJ401490 | KP992374 | OR522147 |
| MT589286 | MN799983 | MK892916 | MN797271 | MH028278 | KT378673 | KT379275 | KM258712 | KR187691 | OR521850 |
| MT589435 | MT589011 | MK892901 | MN797916 | MH028271 | KT192010 | KT379202 | KP250821 | KF857398 | OR521846 |
| MT589716 | MN802972 | MK892898 | MN798066 | MH028265 | KT192028 | KT379145 | KJ401526 | KP992417 | OR521786 |
| MT589343 | MN802622 | MK892897 | MN797416 | MH028263 | KT192009 | KT379028 | KJ401458 | KC352121 | OP190456 |
| MT589016 | MN801067 | MK892880 | MN797256 | MH028260 | KX505973 | KT378926 | KJ401447 | KJ401652 | OP329421 |
| MT589204 | MT589115 | MK892877 | MN799602 | MH028313 | KU050665 | KT378910 | KJ401525 | KR188410 | MW957869 |
| MT588941 | MN801317 | MK892947 | MN797251 | MH028258 | KT191998 | KT378845 | KJ401517 | KR188337 | MW957739 |
| MT589735 | MN801651 | MK892865 | MN799730 | MH028310 | KU050399 | KR823791 | KR822908 | KR188330 | MW957773 |
| MT589202 | MT590209 | MK892882 | MN798881 | MH028286 | KR824018 | KR823436 | KM258714 | KR188277 | OP830979 |
| MT590149 | MT590258 | MK892868 | MN797795 | MH028255 | KR823978 | KR823319 | KR822890 | KR188195 | OP830908 |
| MT590260 | MT589173 | MN426398 | MN797775 | MK286994 | KR823990 | KR823230 | KR822905 | KR188137 | OR522248 |
| MT590001 | MN800944 | MN601647 | MN797745 | MG787433 | KR823952 | KR823224 | KP418629 | KR188019 | OR522199 |
| MT590000 | MT589937 | MN601646 | MN797273 | MG706964 | KR823937 | KR823210 | KF267612 | KR187866 | OP190587 |
| MT589816 | MT589027 | MN425837 | MN797361 | MG706953 | KR823924 | KR823197 | KM258770 | KR187831 | OP190586 |
| MT589814 | MN802139 | MN425809 | MN797239 | MG706970 | KR823940 | KR823189 | KM258809 | KR187684 | MW957893 |
| MT589112 | MN800739 | MN425800 | MN797982 | MG706928 | KR823915 | KR823185 | KP250758 | KR187654 | OR521714 |
| MT589153 | MN800381 | MN425753 | MN797235 | MG706925 | KT379490 | KR823170 | KJ570785 | KR187618 | OR521467 |
| MT589752 | MN800195 | MN425740 | MN797624 | MG706911 | KR823904 | KR823169 | KJ401502 | KR187600 | OP190498 |
| MT590197 | MT588965 | MN425725 | MN797224 | MG706904 | KT379625 | KR823159 | KP250745 | KR187276 | OP190607 |
| MT589767 | MT589333 | MN425711 | MN799039 | MG706940 | KT378716 | KT379724 | KJ401565 | KC888590 | OR522311 |
| MT589741 | MN801451 | MN425710 | MN798557 | MG706899 | KR823876 | KT379033 | KJ401418 | KC888535 | OR521986 |
| MT589598 | MN800771 | MN425697 | MN797213 | MG706868 | KT379616 | KR823951 | KM258745 | KC888427 | MW957917 |
| MT589601 | MN800659 | MN425686 | MN799266 | MG706851 | KR823874 | KR823440 | KF267633 | KC352145 | OR522388 |
| MT588955 | MN800373 | MN425656 | MN797681 | MG706962 | KT379602 | KR823192 | KM258702 | KR188140 | OR522353 |
| MT589432 | MN802507 | MN425649 | MN797240 | MG706784 | KR823871 | KR823093 | KM258872 | KR187841 | OR521811 |
| MT589477 | MT590256 | MN425646 | MN797212 | MG706778 | KT379581 | KR823971 | KM258823 | KR187738 | OR522430 |
| MT589018 | MN802196 | MN425641 | MN798278 | MG706772 | KR823870 | KT379824 | KM258812 | KR187580 | OR521973 |
| MT589006 | MT589064 | MN425625 | MN798705 | MG706761 | KT379589 | KT379730 | KJ570787 | KR187442 | OR521383 |
| MT161965 | MN801933 | MN425618 | MN797985 | MG706903 | KT379574 | KT378679 | KM258799 | KR106846 | OR521834 |
| MT161899 | MN800704 | MN425616 | MN797831 | MG706891 | KT378727 | KR823438 | KP250776 | KR188221 | MW957891 |
| MT161939 | MN800494 | MN425615 | MN797662 | MG706750 | KR823869 | KT379438 | KJ401543 | KR187913 | MW957927 |
| MT161880 | MN800269 | MN425614 | MN797168 | MG706737 | KT379549 | KR823704 | KJ614189 | KR187616 | OP830924 |
| MT161870 | MT986776 | MN425608 | MN797312 | MG706733 | KR823868 | KR823332 | KJ401557 | KR187428 | OR522251 |
| MT161901 | MT588930 | MN425603 | MN797158 | MG706919 | KR823956 | KT379638 | KJ401471 | KR187361 | OR522241 |
| MT161686 | MN801593 | MN425601 | MN797319 | MG706816 | KT379528 | KT379112 | KP250752 | KR187217 | OL684403 |
| MT590169 | MN801300 | MN425258 | MN797154 | MG706794 | KR823862 | KT378975 | KP250737 | KC888655 | OP191543 |
| MT590240 | MN800575 | MN425107 | MN799276 | MG706791 | KT379519 | KT378973 | KJ401482 | KC888560 | OR521751 |
| MT589015 | MN800451 | MN424902 | MN798794 | MG706709 | KR823860 | KT378733 | KM258691 | KC352148 | OR521662 |
| MT590159 | MN802667 | MN424803 | MN797149 | MG706937 | KT379457 | KT378691 | KJ401515 | KJ401684 | MW957672 |
| MT589771 | MN802077 | MN424797 | MN797260 | MG706960 | KR823857 | KR823832 | KR822934 | KJ401767 | OP831017 |
| MT590063 | MN800060 | MN424770 | MN799492 | MG706654 | KT379424 | KR823796 | KR822862 | KR188130 | OP979910 |
| MT589360 | MN802938 | MN424767 | MN799376 | MG706883 | KR823855 | KR823450 | KJ401520 | KR187373 | OR522201 |
| MT589346 | MN802156 | MN424764 | MN798051 | MG706629 | KT379414 | KR823330 | KJ401449 | KR188374 | OR521764 |
| MT590273 | MN801446 | MN424762 | MN797560 | MG706934 | KR823854 | KR823176 | KM258813 | KR188146 | OR521670 |
| MT589251 | MN801374 | MN424758 | MN797144 | MG706930 | KT379390 | KR823919 | KR822893 | KR187901 | OR521681 |
| MT590132 | MT986132 | MN601585 | MN797226 | MG706929 | KR823852 | KX791614 | KR822866 | KR187900 | OR521991 |
| MT589968 | MN801479 | MN601582 | MN797128 | MG706783 | KT379386 | KR823135 | KM258776 | KR187579 | MW957877 |
| MT589787 | MT589686 | MK459117 | MN797850 | MG706693 | KR823851 | KR823141 | KJ401432 | KF857418 | OR522221 |
| MT589560 | MN801637 | MK459057 | MN797170 | MG706602 | KT379850 | KR823120 | KJ401486 | KF857404 | OR522188 |
| MN803048 | MN802090 | MK948159 | MN797124 | MG706963 | KT378672 | KT379784 | KM258739 | KF857372 | OR522186 |
| MN803012 | MN801571 | MK459226 | MN797799 | MG706601 | KT378645 | KT378783 | KP250775 | KR188234 | MW957890 |
| MN803034 | MN800429 | MN426477 | MN797107 | MG706590 | KR823848 | KU954706 | KJ401514 | KR187808 | OR522232 |
| MN802995 | MN800885 | MK459274 | MN797849 | MG706589 | KT379624 | KR823728 | KJ401423 | KR187729 | OR521717 |
| MN803032 | MN800412 | MK459251 | MN797590 | MH921170 | KT379190 | KU954620 | KR822924 | KR187475 | OR521381 |
| MN802914 | MN801313 | MK892869 | MN797097 | MG706585 | KT378717 | KU050546 | KR822923 | KR187207 | OR522233 |
| MN803028 | MN802063 | MN425324 | MN797095 | MG706728 | KR823847 | KU050437 | KM258719 | KC888703 | MW957808 |
| MN802879 | MT589124 | MN426741 | MN797090 | MG706726 | KR823846 | KU050343 | KR822897 | KC888700 | MW957793 |
| MN802892 | MT589171 | MN426687 | MN797833 | MG706606 | KT378713 | KT379722 | KR822869 | KC888665 | MW957889 |
| MN802855 | MT589243 | MN426400 | MN797085 | MG706582 | KR823845 | KT379419 | KJ614117 | KC352097 | OP058640 |
| MN802856 | MN802888 | MN427181 | MN798282 | MG706912 | KT378710 | KT378963 | KJ401465 | KJ401726 | OQ513588 |
| MN802828 | MN802779 | MK892921 | MN797074 | MG706575 | KT379473 | KT378960 | KR605045 | KR188334 | OQ513544 |
| MN802818 | MN802533 | MK892904 | MN799047 | MG706544 | KT379417 | KT378919 | KJ401601 | KR188086 | OK011724 |
| MN802814 | MN802446 | MK892946 | MN798977 | MG706968 | KT378707 | KT378873 | KJ401692 | KR187955 | OP058398 |
| MN802815 | MN802381 | MK892930 | MN797073 | MG706538 | KR823842 | KR823927 | KJ401535 | KR187940 | OP058359 |
| MN802808 | MN802232 | MK543595 | MN798635 | MG706534 | KT379134 | KR823227 | KJ401513 | KR187753 | OP058308 |
| MT588967 | MN802177 | MK459283 | MN798089 | MG706533 | KT378702 | KR822973 | KM258700 | KR187751 | OP329536 |
| MN802746 | MN801929 | MN426839 | MN797502 | MG706518 | KR823838 | KU954561 | KM258753 | KR187201 | OP190748 |
| MN802988 | MN801922 | MN426804 | MN797309 | MG706562 | KT379038 | KU050419 | KM258777 | KC888704 | OP058365 |
| MN802725 | MN801912 | MN426688 | MN797072 | MG706494 | KT378818 | KT379367 | KJ570820 | KC888661 | OK011695 |
| MN802705 | MN801815 | MN426560 | MN798041 | MG706502 | KT378701 | KT379154 | KJ614097 | KC888605 | ON736463 |
| MN802693 | MN801638 | MN426494 | MN797057 | MG706487 | KR823836 | KT378893 | KM258789 | KC888568 | OP058396 |
| MN803135 | MN801537 | MN426493 | MN797098 | MG706476 | KT379529 | KR823637 | KR822877 | KC888548 | OK011581 |
| MN802677 | MN801530 | MN426428 | MN797055 | MG706475 | KT379421 | KR823596 | KM258732 | KR188101 | OK011635 |
| MN802931 | MN801376 | MN426389 | MN797787 | MG706946 | KT379116 | KR823567 | KM258763 | KC352154 | OP058521 |
| MN802673 | MN801260 | MN426334 | MN797045 | MG706657 | KT378699 | KR824016 | KJ570795 | KR188318 | OQ513562 |
| MN802631 | MN801096 | MN426067 | MN797528 | MG706468 | KR823835 | KU954755 | KM258801 | KP992396 | OQ513468 |
| MN802630 | MN801062 | MN426789 | MN797043 | MG706505 | KT378698 | KU954627 | KR822913 | KP992385 | OR521556 |
| MN802606 | MN800655 | MN426558 | MN799702 | MG706464 | KR823834 | KU954610 | KR822857 | KC888649 | OP058546 |
| MN802605 | MN800580 | MK892929 | MN799521 | MG706662 | KT378693 | KU954609 | KP418625 | KP992345 | OP058266 |
| MN802820 | MN800399 | MN425842 | MN799295 | MG706463 | KR823833 | KU954589 | KP250772 | KJ401645 | OQ513472 |
| MN802595 | MN800336 | MN425763 | MN798517 | MH388438 | KT379141 | KU954582 | KP250697 | KR188169 | OK011660 |
| MN802678 | MN800287 | MN425696 | MN797041 | MG706848 | KR823831 | KU954567 | KF835231 | KR188050 | OP329644 |
| MN802566 | MN800167 | MN425399 | MN797567 | MG706703 | KT378684 | KU050673 | KF835163 | KR187898 | OQ513539 |
| MN802679 | MN800095 | MN425378 | MN797038 | MG706694 | KR823830 | KU050503 | KJ570842 | KR188340 | OR521639 |
| MN802564 | MN800052 | MN425354 | MN798448 | MG706474 | KT379119 | KT379872 | KJ570789 | KR106879 | OK011605 |
| MN802552 | MN799972 | MN425329 | MN798272 | MG706470 | KT378982 | KT379854 | KP250779 | KR106847 | OP979644 |
| MN802736 | MN799965 | MN425321 | MN797028 | MG706456 | KR823827 | KT379799 | KJ401538 | KR188436 | MW957721 |
| MN802627 | MN799828 | MN425311 | MN797059 | MG519330 | KR823823 | KT379745 | KJ401566 | KR187904 | OQ513515 |
| MN802497 | MN800577 | MN425099 | MN797026 | MG064457 | KT379082 | KT379735 | KJ614021 | KR187703 | OP190735 |
| MN802927 | MN801768 | MN425098 | MN797591 | MH300306 | KR823820 | KT379732 | KM258742 | KR187566 | OP058250 |
| MN802460 | MT589085 | MN424958 | MN798010 | MF941494 | KT379064 | KT379726 | KM258756 | KR187478 | OP058162 |
| MN802459 | MN803033 | MN424947 | MN797024 | MH010705 | KR823818 | KT379712 | KJ570827 | KR187435 | OQ513546 |
| MN803086 | MN802286 | MN424921 | MN799371 | MF941443 | KR823817 | KT379599 | KM258676 | KR187254 | OP830952 |
| MN803074 | MT986181 | MN424900 | MN797016 | MH573267 | KT378705 | KT379596 | KJ614191 | KR187232 | OL814948 |
| MN802447 | MT986177 | MN424897 | MN797205 | MF941412 | KR823816 | KT379536 | KM258834 | KR187221 | OL814607 |
| MN802431 | MN801652 | MN424885 | MN797008 | MF941368 | KT379406 | KT379505 | KJ570832 | KC888679 | OK011606 |
| MN802684 | MN800934 | MN424867 | MN799347 | MF941367 | KR823849 | KT379449 | KJ570826 | KC888674 | OP058511 |
| MN802635 | MN800415 | MN424855 | MN797054 | MF941360 | KR823826 | KT379437 | KJ570813 | KC888567 | OP058491 |
| MN802408 | MN800157 | MN424832 | MN796994 | MF941314 | KT379306 | KT379432 | KJ570802 | KC888247 | OP058457 |
| MN802366 | MN799832 | MN424795 | MN797493 | MF941261 | KT379153 | KT379428 | KF250372 | KR188352 | OP058368 |
| MN802338 | MN801100 | MN424794 | MN797121 | MF941242 | KT379114 | KT379422 | KJ401468 | KR188303 | OP058284 |
| MN803064 | MT589166 | MN424786 | MN796992 | MF684313 | KT379042 | KT379350 | KM258688 | KR188194 | OP058281 |
| MN802319 | MT589595 | MN424782 | MN796984 | MF684312 | KT378964 | KT379308 | KP250817 | KR188191 | OP058269 |
| MN802224 | MT588950 | MN601559 | MN797649 | MG706769 | KR823804 | KT379297 | KP250782 | KR188188 | OP058208 |
| MN802218 | MT590277 | MK459158 | MN796968 | MF684299 | KT379772 | KT379278 | KP250754 | KR188186 | OK011634 |
| MN802239 | MT590207 | MK459188 | MN797387 | MF684294 | KT379450 | KT379255 | KP250734 | KR188115 | OK011726 |
| MN802215 | MN802521 | MK458993 | MN796955 | MF684291 | KR823803 | KT379216 | KP250717 | KR187969 | MW957926 |
| MN802861 | MN801974 | MK459259 | MN797042 | MG706826 | KT379955 | KT379203 | KP250681 | KR187951 | MW957682 |
| MN802919 | MT589412 | MK459095 | MN798206 | MF684278 | KT379217 | KT379199 | KJ401559 | KR187849 | MW957769 |
| MN802726 | MT590156 | MK459090 | MN796946 | MF684277 | KT378999 | KT379180 | KP250794 | KR187830 | OQ513552 |
| MN802424 | MN802579 | MK458985 | MN797231 | MF684271 | KR823801 | KT379168 | KJ401425 | KR187771 | OP058480 |
| MN802356 | MN802190 | MK458963 | MN796942 | MG706673 | KX583303 | KT379104 | KM258754 | KR187746 | OP058438 |
| MN802172 | MN800510 | MN426817 | MN796953 | MF684254 | KT379003 | KT379075 | KJ401760 | KR187736 | OP058375 |
| MN802152 | MT589651 | MN425193 | MN796940 | MF684253 | KR823800 | KT379058 | KJ401718 | KR187656 | OP058344 |
| MN802151 | MT986084 | MK459044 | MN799530 | MG706584 | KT379352 | KT379041 | KJ401744 | KR187634 | OP058330 |
| MN802853 | MT590294 | MN601667 | MN796939 | MF684250 | KT378986 | KT379039 | KJ401754 | KR187630 | OP058323 |
| MN802103 | MT588920 | MN424837 | MN798057 | MF684225 | KR823798 | KT379030 | KJ401758 | KR187611 | MW957811 |
| MN803003 | MT589847 | MN424811 | MN797011 | MF684224 | KT378959 | KT379014 | KJ364642 | KR187610 | OQ513530 |
| MN802087 | MT589972 | MN425483 | MN797466 | MG706669 | KR823795 | KT379005 | KF857453 | KR187586 | OK011596 |
| MN803031 | MN803117 | MN425291 | MN796930 | MF684197 | KT379342 | KT379004 | KF857452 | KR187576 | OP830970 |
| MN802985 | MN802904 | MN425271 | MN797653 | MF684196 | KT378940 | KT378984 | KF857460 | KR187541 | OK011629 |
| MN802060 | MN802890 | MN426803 | MN796910 | MG706776 | KR823794 | KT378983 | KF857406 | KR187535 | OP058527 |
| MW270702 | MN802845 | MN426646 | MN799314 | MG706697 | KT378899 | KT378945 | KF857400 | KR187528 | OP058512 |
| MN802029 | MN802819 | MK459094 | MN796906 | MG706686 | KR823792 | KT378909 | KF857442 | KR187499 | OP058441 |
| MN802864 | MN802776 | MK459248 | MN796913 | MF684249 | KR823786 | KT378835 | KF857429 | KR187462 | OP058425 |
| MN802024 | MN802768 | MN427002 | MN796905 | MF684182 | KR823776 | KT378832 | KF857424 | KR187447 | OP058424 |
| MN803000 | MN802576 | MN426965 | MN797392 | MF684195 | KU050417 | KT378795 | KF857438 | KR187415 | OP058408 |
| MN802534 | MN802503 | MN426903 | MN797902 | MF684180 | KR823769 | KT378786 | KF857408 | KR187405 | OP058388 |
| MN802836 | MN802502 | MN426894 | MN797870 | MF684181 | KT379605 | KT378785 | KF857407 | KR187402 | OP058379 |
| MN802602 | MN802492 | MN426863 | MN796901 | MF684176 | KT379373 | KT378774 | KF857392 | KR187379 | OP058377 |
| MN801998 | MN802198 | MN426823 | MN798319 | MF684167 | KT378849 | KT378770 | KF857414 | KR187359 | OP058351 |
| MN802013 | MN802130 | MN426821 | MN796895 | MF684166 | KR823873 | KT378737 | KF857389 | KR187351 | OP058277 |
| MN801989 | MN802119 | MN426747 | MN796981 | MF684247 | KR823762 | KT378671 | KF857451 | KR187284 | OP058257 |
| MN801968 | MN802091 | MN426743 | MN796890 | MF684162 | KT379461 | KT378669 | KF857386 | KR187283 | MW957680 |
| MN801967 | MN802082 | MN426717 | MN798974 | MG706633 | KR823759 | KT378664 | KF857384 | KR187280 | OQ513599 |
| MN801962 | MN802054 | MN426710 | MN797406 | MF684161 | KX583304 | KT378653 | KF857425 | KR187266 | OQ513483 |
| MN801953 | MN802038 | MN426709 | MN796885 | MG706885 | KR823757 | KT378642 | KF857458 | KR187265 | OP058391 |
| MN802955 | MN801825 | MN426698 | MN797661 | MG706660 | KX583282 | KR823856 | KF857422 | KR187259 | OP058353 |
| MN801948 | MN801772 | MN426679 | MN796884 | MH921166 | KR823785 | KR823853 | KF857426 | KR187247 | OP058329 |
| MN801942 | MN801765 | MN426644 | MN798333 | MF684135 | KR823748 | KR823797 | KF857399 | KR187246 | OP058328 |
| MN802043 | MN801747 | MN426640 | MN798182 | MG706759 | KR823747 | KR823793 | KF857388 | KR187214 | OP058310 |
| MN801927 | MN801519 | MN426609 | MN797961 | MF684134 | KR823755 | KR823738 | KF857421 | KC888739 | OP058289 |
| MN802105 | MN801440 | MN426604 | MN797185 | MF684123 | KR823746 | KR823631 | KF857370 | KC888438 | OR521613 |
| MN801894 | MN801425 | MN426603 | MN796872 | MF684122 | KR823719 | KR823623 | KF857387 | KJ401768 | ON736610 |
| MN803005 | MN801411 | MN426598 | MN796875 | MF684185 | KR823968 | KR823557 | KF857378 | KJ401710 | OQ513486 |
| MN803002 | MN801393 | MN426595 | MN796870 | MF684121 | KR823710 | KR823515 | KF857409 | KJ401681 | OP058331 |
| MN801878 | MN801143 | MN426581 | MN797796 | MG706646 | KR823713 | KR823433 | KJ401509 | KJ401655 | OP058528 |
| MT589033 | MN801115 | MN426579 | MN796869 | MF684105 | KU050624 | KR823333 | KJ401694 | KR188104 | OP058476 |
| MN801863 | MN801023 | MN426562 | MN799187 | MG706520 | KU050336 | KR823324 | KR188423 | KR188060 | OQ513573 |
| MN802516 | MN801012 | MN426538 | MN798202 | MF684179 | KR823680 | KR823254 | KR188409 | KR187965 | OR521596 |
| MN801860 | MN801004 | MN426528 | MN797659 | MF684098 | KR823677 | KR823172 | KR188406 | KR187943 | MZ359107 |
| MN801851 | MN800990 | MN426516 | MN797505 | MG706920 | KR823668 | KR823155 | KR188366 | KR187864 | OR521624 |
| MN801850 | MN800942 | MN426511 | MN797338 | MG706908 | KR823660 | KR822985 | KR188365 | KR187836 | OP058560 |
| MN802920 | MN800911 | MN426495 | MN797161 | MG706860 | KR823662 | KR822959 | KJ401732 | KR187824 | OP058652 |
| MN801952 | MN800733 | MN426478 | MN797005 | MG706779 | KR823646 | KU050484 | KR188331 | KR187789 | OP058643 |
| MN801788 | MN800729 | MN426418 | MN796862 | MG706751 | KR823639 | KU050470 | KR188325 | KR187769 | OP058587 |
| MN801784 | MN800683 | MN426413 | MN798764 | MG706702 | KR823642 | KU050451 | KR188321 | KR187761 | OP058559 |
| MN802399 | MN800671 | MN426412 | MN797382 | MG706613 | KR823640 | KU050371 | KR188293 | KR187750 | MW957829 |
| MN801775 | MN800666 | MN426411 | MN797012 | MG706572 | KR823627 | KR823392 | KR188430 | KR187749 | OQ513481 |
| MN801868 | MN800470 | MN426402 | MN796938 | MG706531 | KR823661 | KR823064 | KR188205 | KR187706 | OQ513529 |
| MN801761 | MN800396 | MN426395 | MN796857 | MG706499 | KR823649 | KR822995 | KR188263 | KR187688 | OP058535 |
| MN802506 | MN800387 | MN426391 | MN797504 | MF684202 | KR823626 | KT379436 | KR188200 | KR187679 | OR521565 |
| MN802481 | MN800357 | MN426381 | MN797335 | MF684175 | KU050414 | KR823618 | KR188230 | KR187655 | OR521563 |
| MN801741 | MN800356 | MN426377 | MN796848 | MF684156 | KR823621 | KU954722 | KR188187 | KR187609 | OR521552 |
| MN801792 | MN800161 | MN426354 | MN799312 | MF684097 | KR823931 | KT379808 | KR188165 | KR187589 | OP921950 |
| MN801718 | MN800122 | MN426348 | MN797912 | MF684133 | KR823607 | KT379798 | KR188164 | KR187585 | OR521490 |
| MN801674 | MN800110 | MN426344 | MN797018 | MF684094 | KR823612 | KT379751 | KR188402 | KR187561 | OP190747 |
| MN801671 | MN799933 | MN426342 | MN796937 | MG706492 | KR823605 | KT379570 | KR188160 | KR187555 | OP058472 |
| MN801905 | MN799902 | MN426333 | MN796936 | MF684092 | KR823587 | KT379507 | KR188159 | KR187540 | OP191130 |
| MN801667 | MN799791 | MN426328 | MN796841 | MF684192 | KY226024 | KT379472 | KR188158 | KR187480 | OP058531 |
| MN801649 | MN799786 | MN426318 | MN799569 | MF684081 | KR823569 | KT379459 | KR188232 | KR187335 | OP058280 |
| MN802905 | MN801856 | MN426300 | MN798738 | MF684251 | KR823562 | KT379389 | KR188125 | KR187325 | OP058264 |
| MN802436 | MT589135 | MN426251 | MN796840 | MF684078 | KT379559 | KT379382 | KR188210 | KR187249 | MW957822 |
| MN801669 | MN802026 | MN426245 | MN799753 | MG706685 | KT379455 | KT379370 | KR188167 | KR187243 | MW957862 |
| MN801627 | MN801963 | MN426229 | MN796834 | MG706617 | KR823547 | KT379369 | KR188154 | KR187237 | MW957800 |
| MN801733 | MN801463 | MN426228 | MN799013 | MF684077 | KT379215 | KT379351 | KR188117 | KR187193 | OL814664 |
| MN801605 | MN800628 | MN426201 | MN799610 | MG706762 | KR823543 | KT379267 | KR188397 | KR187189 | OP058525 |
| MN801950 | MN799908 | MN426184 | MN796830 | MF684060 | KY226081 | KT379251 | KR188447 | KR187187 | OP058520 |
| MN801757 | MN799890 | MN426177 | MN799703 | MG706790 | KR823542 | KT379201 | KR188193 | KC888741 | OP058499 |
| MN801663 | MN799826 | MN426173 | MN799623 | MG706723 | KR823563 | KT379181 | KR188078 | KC888727 | OP058494 |
| MN801584 | MT589625 | MN426164 | MN799511 | MG706688 | KR823540 | KT379166 | KR188219 | KC888724 | OP058468 |
| MN801608 | MN803121 | MN426159 | MN799456 | MG706614 | KT379130 | KT379090 | KR188052 | KC888716 | OP058460 |
| MN801570 | MN801880 | MN426157 | MN799105 | MF684178 | KT379520 | KT379017 | KR188418 | KC888714 | OP058406 |
| MN801564 | MN799845 | MN426156 | MN799062 | MF684059 | KR823526 | KT379012 | KR188043 | KC888694 | OP058397 |
| MN801524 | MT986168 | MN426152 | MN798619 | MF684051 | KR823517 | KT379006 | KR188183 | KC888683 | OP058393 |
| MN801528 | MT986321 | MN426145 | MN798520 | MF503234 | KR823523 | KT378949 | KR188040 | KC888681 | OP058380 |
| MN801521 | MT589168 | MN426144 | MN798449 | MF503217 | KR823544 | KT378844 | KR188204 | KC888666 | OP058369 |
| MN802851 | MN803119 | MN426141 | MN797943 | MF503173 | KR823513 | KR823726 | KR188355 | KC888663 | OP058307 |
| MN801509 | MN802801 | MN426133 | MN797858 | MF503171 | KR823530 | KR823533 | KF857461 | KC888659 | OP058297 |
| MN801484 | MN801943 | MN426131 | MN797756 | MG450645 | KR823520 | KR823476 | KR188017 | KC888632 | OP058295 |
| MN802918 | MN801690 | MN426112 | MN797722 | MG450621 | KR823546 | KR823469 | KR188157 | KC888627 | OP058282 |
| MN801737 | MN801646 | MN426111 | MN797592 | MG450619 | KR823509 | KU050483 | KR188006 | KC888603 | OP058259 |
| MN801458 | MN801645 | MN426108 | MN797531 | MG450601 | KR823503 | KU050365 | KF857444 | KC888589 | OP190754 |
| MN801477 | MN801590 | MN426093 | MN797478 | MF503154 | KR823497 | KU050309 | KR188003 | KC888572 | OP058537 |
| MN801445 | MN801464 | MN426088 | MN797285 | MF325064 | KR823504 | KT379951 | KR188002 | KC888564 | OP058403 |
| MN801433 | MN801131 | MN426086 | MN797184 | MF325063 | KR823493 | KT379694 | KR188311 | KC888552 | OR521621 |
| MN801874 | MN801040 | MN426077 | MN796863 | MF037196 | KR823984 | KT379591 | KR188132 | KC888468 | MW957691 |
| MN801431 | MN800579 | MN426062 | MN796819 | MF037195 | KR823489 | KT379445 | KR188062 | KC888466 | OP058541 |
| MN801717 | MN800299 | MN426055 | MN798497 | MH300439 | KU050197 | KT379365 | KR188032 | KC888460 | OP058538 |
| MN801424 | MN800249 | MN426053 | MN796816 | MF037202 | KR823484 | KT379348 | KR187999 | KC888450 | OP058536 |
| MN801392 | MT161811 | MN426051 | MN797439 | MF037194 | KR823566 | KT379263 | KR188421 | KC888430 | OP058450 |
| MN802138 | MT590264 | MN426046 | MN797412 | MH573340 | KR823481 | KT379248 | KR188127 | KC888373 | OP058422 |
| MN801822 | MN800785 | MN426043 | MN796810 | MF037167 | KU954699 | KT379240 | KR187972 | KC352218 | OP058274 |
| MN801818 | MN803136 | MN426033 | MN797619 | MF037157 | KR823478 | KT379158 | KR187989 | KC352206 | OP058265 |
| MN801383 | MN802535 | MN426032 | MN797292 | MH573321 | KR823477 | KT379147 | KR187987 | KC352188 | OR521570 |
| MN801944 | MN802316 | MN426031 | MN797091 | MF037142 | KT379236 | KT378906 | KR187963 | KC352184 | OP058509 |
| MN801366 | MN802200 | MN426001 | MN796806 | MH573324 | KR823465 | KT378892 | KR188142 | KC352150 | OP058387 |
| MN802132 | MN799929 | MN425998 | MN798377 | MF037137 | KR823574 | KT378882 | KR187954 | KC352149 | OP058419 |
| MN801353 | MT589214 | MN425990 | MN797483 | MH573516 | KR823463 | KT378866 | KR188026 | KC352130 | OP058303 |
| MN801896 | MN801471 | MN425975 | MN796832 | MF037201 | KR823654 | KT378802 | KR187952 | KC352127 | OP058650 |
| MN801352 | MN801323 | MN425974 | MN796798 | MF037132 | KR823462 | KR823156 | KR188273 | KC352126 | OP058633 |
| MN802536 | MT589587 | MN425972 | MN797911 | MF037130 | KT379517 | KR823589 | KR187936 | KC352110 | OP058626 |
| MN802267 | MN800143 | MN425971 | MN796795 | MF037122 | KR823461 | KR823379 | KR188112 | KC352104 | OP058603 |
| MN801298 | MT590153 | MN425969 | MN797295 | MF037118 | KT379923 | KR823903 | KR188214 | KC352101 | OP058602 |
| MN801679 | MN803114 | MN425924 | MN796787 | MF037117 | KT379182 | KR823516 | KR187912 | KC352100 | OP058586 |
| MN801253 | MN802971 | MN425922 | MN799380 | MH028370 | KR823602 | KT379618 | KR188308 | KC352098 | OP058551 |
| MN802672 | MN802798 | MN425896 | MN796778 | MF037178 | KR823568 | KR823875 | KF857445 | KC352082 | OP191124 |
| MN802416 | MN802179 | MN425890 | MN796774 | MF037165 | KR823518 | KR823318 | KR187893 | KC352080 | OP058374 |
| MN801248 | MN802009 | MK409825 | MN796773 | MF037105 | KR823514 | KX583283 | KR187879 | KC352073 | OP058373 |
| MN801773 | MN801835 | MN601669 | MN798874 | MF037181 | KR823472 | KT379860 | KR188231 | KC888585 | OQ513527 |
| MN802992 | MN801121 | MN601645 | MN796758 | MF037097 | KR823459 | KT379262 | KR187874 | KR188235 | OP058473 |
| MN801201 | MN801080 | MN601642 | MN796943 | MF037076 | KR823448 | KR823885 | KR188260 | KC888456 | OP058437 |
| MN801200 | MN800830 | MK543669 | MN796756 | MF037101 | KT379678 | KR823697 | KR188070 | KR187799 | OP058449 |
| MN802625 | MN800818 | MK543636 | MN796755 | MF037085 | KT378942 | KR823281 | KR188213 | KR187643 | OP058367 |
| MN801187 | MN800738 | MK543581 | MN797388 | MF037075 | KR823599 | KR823737 | KR188446 | KR187493 | OP058532 |
| MN801730 | MN800600 | MK543565 | MN797084 | MH028378 | KR823460 | KY226141 | KR187818 | KR187417 | OP058434 |
| MN801172 | MN800319 | MK543563 | MN796752 | MF037106 | KR823444 | KT379329 | KR187962 | KR187653 | MW957806 |
| MN802585 | MN800212 | MK459281 | MN797220 | MF037073 | KT379832 | KU050290 | KR187816 | KR187619 | MW957883 |
| MN801168 | MN800097 | MK459280 | MN796748 | MH028321 | KT379804 | KT379902 | KR187926 | KR187356 | MW957921 |
| MN802466 | MN799852 | MK459224 | MN798804 | MF037091 | KR823441 | KT379786 | KR187910 | KR187286 | OQ513617 |
| MN801165 | MN800984 | MK459213 | MN797672 | MF037072 | KR823439 | KT379548 | KR187809 | KC888718 | OR521605 |
| MN802933 | MN800068 | MK948172 | MN797133 | MH028304 | KT379164 | KT379425 | KR187976 | KC888712 | OL814700 |
| MN802341 | MN802368 | MK948171 | MN796882 | MF037068 | KR823413 | KT379313 | KR187807 | KC888701 | OK011617 |
| MN801141 | MN800895 | MN424949 | MN796744 | MF037063 | KX791565 | KT379238 | KR188435 | KC888690 | OP058443 |
| MN801339 | MN800802 | MK459153 | MN796907 | MH028257 | KT379219 | KT379159 | KR187803 | KC888685 | OP058423 |
| MN802023 | MN801755 | MK459106 | MN796743 | MF037051 | KR823404 | KT378806 | KR187882 | KC888672 | OP058412 |
| MN801118 | MT589898 | MK458946 | MN799074 | MF037038 | KR823429 | KT378742 | KR187948 | KC888412 | OP058360 |
| MN801681 | MN802548 | MN426962 | MN797453 | MH573318 | KT379513 | KT378670 | KR187778 | KC888226 | OP058321 |
| MN801680 | MN802896 | MN426407 | MN796741 | MF037179 | KR823859 | KR823805 | KR187930 | KC352081 | OP058301 |
| MN801116 | MN802262 | MN426378 | MN797036 | MF037055 | KR823399 | KR823222 | KR187773 | KC888680 | OP058294 |
| MN802188 | MN801630 | MN426343 | MN796732 | MF037035 | KR823815 | KR823974 | KR188392 | KC888588 | MW957961 |
| MN801439 | MN801357 | MN426339 | MN799413 | MH028380 | KR823396 | KT379480 | KR187768 | KC352103 | OP058495 |
| MN802061 | MN800218 | MN426301 | MN796960 | MH028311 | KR823395 | KT379393 | KR188376 | KC888660 | OP058461 |
| MN801119 | MN800065 | MN426243 | MN796730 | MF037174 | KR823394 | KT379295 | KR187914 | KR187832 | OM454559 |
| MN803142 | MT589875 | MN426230 | MN798325 | MF037127 | KR823393 | KT379222 | KR187727 | KC888722 | MW953659 |
| MN803141 | MT589558 | MN426182 | MN798309 | MF037065 | KX583293 | KT379170 | KR187715 | KC888711 | MW953625 |
| MN801094 | MT986145 | MN426161 | MN798130 | MF037056 | KR823391 | KT379138 | KR187977 | KC888607 | MW953709 |
| MN801468 | MN802877 | MN426146 | MN796719 | MF037029 | KX791611 | KT378991 | KR187713 | KC888597 | MW952469 |
| MN801091 | MN801670 | MN426115 | MN799102 | MF037025 | KT379671 | KR823293 | KR187929 | KC888594 | MW951846 |
| MN802093 | MN801082 | MN426050 | MN796713 | KY972142 | KR823675 | KU050537 | KR187801 | KC352162 | MW952445 |
| MN801882 | MN800384 | MN425995 | MN799323 | KY972141 | KR823430 | KU050522 | KR187798 | KC352116 | MW954901 |
| MN801090 | MN800316 | MN425968 | MN798808 | MG706655 | KR823408 | KU050366 | KR187722 | KF857455 | MW954900 |
| MN801883 | MN800224 | MN425933 | MN798197 | KY713580 | KR823406 | KU050304 | KR187681 | KF857448 | MW954800 |
| MN801078 | MT589306 | MN425932 | MN797307 | KY713569 | KR823401 | KU050288 | KR187680 | KF857432 | MW954402 |
| MN801240 | MN801205 | MN425931 | MN799122 | MG706501 | KR823388 | KT379956 | KR187828 | KF857427 | MW954522 |
| MN801073 | MT589471 | MN425894 | MN796702 | KY713568 | KX791630 | KT379954 | KR187834 | KF857420 | MW954030 |
| MN802878 | MN802717 | MN425889 | MN798038 | KY713544 | KR823373 | KT379949 | KR187668 | KF857403 | MW954779 |
| MN801848 | MT589393 | MK459232 | MN797183 | KY713535 | KX010453 | KT379910 | KR188217 | KF857393 | MW953799 |
| MN801051 | MT589317 | MN426906 | MN796693 | KY713510 | KR823365 | KT379906 | KR188282 | KF857376 | MW952408 |
| MN801293 | MN803069 | MN426312 | MN797859 | KY713498 | KR823339 | KT379792 | KR187659 | KF857375 | MW952552 |
| MN801272 | MN802644 | MN426223 | MN796688 | KY713507 | KR823342 | KT379778 | KR188442 | KF857360 | MW953760 |
| MN801034 | MN802530 | MN426080 | MN799733 | KY713496 | KR823317 | KT379765 | KR188139 | KC888656 | MW953259 |
| MN802839 | MN802509 | MK892931 | MN796686 | KY713492 | KY226138 | KT379757 | KR188131 | KC888629 | MW952685 |
| MN802727 | MN802474 | MN426158 | MN798014 | KY713494 | KR823294 | KT379540 | KR187651 | KC888630 | MW952686 |
| MN801030 | MN802454 | MK459183 | MN799541 | KY713458 | KR823279 | KT379501 | KR187642 | KR187620 | MW952709 |
| MN801901 | MN802372 | MK287104 | MN798390 | KY713534 | KY226139 | KT379444 | KR187639 | KR106860 | MW952567 |
| MN801015 | MN802283 | MN601652 | MN796664 | KY713454 | KR823277 | KT379400 | KR187636 | KR187627 | MW953803 |
| MN802135 | MN802256 | MN426525 | MN799567 | KY713452 | KR823571 | KT379315 | KR187875 | KR106825 | MW955166 |
| MN801153 | MN802175 | MK543600 | MN797892 | KY713446 | KR823525 | KT379282 | KR187621 | KC888631 | MW954438 |
| MN801010 | MN802165 | MK459252 | MN796660 | KY713547 | KR823487 | KT379271 | KR187675 | KR187377 | MW955094 |
| MN801634 | MN801990 | MN425583 | MN799230 | KY713406 | KR823268 | KT379270 | KR187612 | KR187707 | MW954657 |
| MN801008 | MN801660 | MK543603 | MN796864 | KY713375 | KT379524 | KT379256 | KR188299 | KR187652 | MW955001 |
| MN803106 | MN801620 | MN425765 | MN796659 | MG706637 | KR823861 | KT379227 | KR188269 | KR187575 | MW954033 |
| MN802101 | MN801545 | MN425500 | MN797333 | KY713357 | KR823265 | KT379225 | KR187852 | KR187297 | MW954035 |
| MN802081 | MN801544 | MN425364 | MN797288 | KY713395 | KR823275 | KT379137 | KR187601 | KR187200 | MW954627 |
| MN801884 | MN801526 | MN425340 | MN796656 | KY713354 | KR823261 | KT378954 | KR187682 | KR188408 | MW954596 |
| MN801497 | MN801513 | MN425297 | MN798374 | MH921083 | KR823739 | KT378950 | KR187597 | KJ401766 | MW954527 |
| MN801465 | MN801423 | MN425200 | MN797025 | KY713348 | KR823242 | KT378931 | KR188346 | KJ401745 | MW954361 |
| MN801098 | MN801341 | MN425188 | MN796669 | MH573323 | KR823226 | KT378925 | KR187631 | KJ401750 | MW954403 |
| MN800989 | MN801269 | MN425168 | MN796649 | MH028296 | KT379604 | KT378874 | KR187583 | KR188388 | MW954064 |
| MN802778 | MN801183 | MN425133 | MN798326 | MF037099 | KT378726 | KT378820 | KR188251 | KR188351 | MW954063 |
| MN801714 | MN801129 | MN425059 | MN796746 | KY244818 | KR823872 | KT378803 | KR188180 | KR188309 | MW952666 |
| MN800979 | MN801057 | MN425048 | MN796646 | MF037094 | KR823225 | KT378759 | KJ401715 | KR188145 | MW952654 |
| MN800961 | MN801049 | MN424973 | MN796871 | KY244817 | KR824015 | KT378665 | KR187562 | KR187699 | MW954973 |
| MN800933 | MN800912 | MN424960 | MN796643 | KY713559 | KR823214 | KR823946 | KR187551 | KR187686 | MW954860 |
| MN802058 | MN800906 | MN424948 | MN799081 | KY244807 | KT379264 | KR823865 | KR187550 | KR187444 | MW954515 |
| MN800926 | MN800862 | MN424945 | MN798447 | MF037113 | KR823362 | KR823821 | KR188266 | KR187191 | MW954789 |
| MN801110 | MN800842 | MN424924 | MN796636 | KY244805 | KR823209 | KR823810 | KR187644 | KC888713 | MW954721 |
| MN800953 | MN800720 | MK459078 | MN797861 | MH573253 | KR823349 | KR823617 | KR187748 | KC888693 | MW954704 |
| MN800903 | MN800713 | MK459161 | MN796635 | MH573245 | KR823215 | KR823352 | KR187571 | KC888565 | MW954674 |
| MN800901 | MN800687 | MN426366 | MN799641 | KY244804 | KR823196 | KR823350 | KR187527 | KC888559 | MW954683 |
| MN801135 | MN800601 | MN426252 | MN798706 | KY244759 | KR823628 | KR823340 | KR188153 | KC888549 | MW954651 |
| MN800883 | MN800520 | MK458996 | MN798249 | MH573543 | KR823181 | KR823310 | KR187860 | KC888532 | MW954446 |
| MN801230 | MN800390 | MN426578 | MN797908 | MH573207 | KR823712 | KR823309 | KR187526 | KC888444 | MW953885 |
| MN800880 | MN800283 | MN426320 | MN797469 | MF037092 | KR823221 | KR823308 | KR187673 | KR187382 | MW954201 |
| MN800935 | MN800245 | MN427274 | MN797467 | MF037071 | KT379221 | KR823301 | KR187530 | KR187258 | MW953833 |
| MN800870 | MN800154 | MN427252 | MN797272 | KY244757 | KT379220 | KR823256 | KR187504 | KC888698 | MW954101 |
| MN801716 | MN800051 | MN426296 | MN796745 | MH573176 | KR823158 | KR823244 | KR187833 | KC352083 | MW954710 |
| MN800863 | MN799966 | MN426214 | MN799696 | KY244748 | KR823150 | KR823239 | KR187770 | OP329645 | MW954755 |
| MN802689 | MN799942 | MN426076 | MN799632 | MH573159 | KR823148 | KR823203 | KR187503 | OP329567 | MW952787 |
| MN800857 | MN799938 | MN426793 | MN799352 | MH573157 | KX791627 | KR823191 | KR188016 | MW957817 | MW954716 |
| MN800898 | MN799913 | MN426729 | MN798963 | KY244735 | KR823138 | KR823187 | KR187827 | MW957880 | MW954131 |
| MN800853 | MN799912 | MN426694 | MN798518 | KY244740 | KX791631 | KR823177 | KR187502 | MW957832 | MW953988 |
| MN801334 | MN799807 | MN426910 | MN798306 | KY244709 | KR823129 | KR823173 | KR187495 | MW957683 | MW954120 |
| MN801216 | MT589390 | MN426705 | MN798166 | KY244701 | KX791635 | KU050372 | KR187494 | MW957874 | MW954935 |
| MN800846 | MT590213 | MN426657 | MN798118 | KY244700 | KR823104 | KT379578 | KR188090 | MW957765 | MW955262 |
| MN802231 | MT589844 | MN426643 | MN797835 | KY244688 | KR823091 | KT379073 | KR188118 | MW957928 | MW954890 |
| MN801415 | MN801138 | MN426570 | MN797805 | KY244687 | KR823753 | KT379072 | KR187850 | MW957875 | MW954217 |
| MN800841 | MN800568 | MN426548 | MN797663 | KY244689 | KR823085 | KT379069 | KR187486 | OR521462 | MW954780 |
| MN801744 | MT589292 | MN426486 | MN797566 | KY244670 | KR823066 | JX960605 | KR187855 | OR521459 | MW956981 |
| MN800826 | MT589531 | MN426347 | MN797441 | MK287002 | KR823052 | JX960604 | KR187744 | OR521469 | MW954445 |
| MN803011 | MT588987 | MN426330 | MN797355 | KY244669 | KR823045 | JX112865 | KR187605 | OR521457 | MW954834 |
| MN801155 | MN803053 | MN426205 | MN797352 | MF503214 | KR823077 | JX112860 | KR187488 | OR521468 | MW954813 |
| MN800825 | MT589977 | MN426203 | MN797195 | KY244620 | KR823032 | JX112852 | KR188027 | OR521455 | MW955283 |
| MT368438 | MN802517 | MN426195 | MN797062 | MH573299 | KX791563 | JX112807 | KR187666 | OR521419 | MW955068 |
| MN800806 | MN802264 | MN426192 | MN797047 | KY244600 | KR823019 | JQ901028 | KR187467 | OR521416 | MW953982 |
| MN800778 | MN802174 | MN426154 | MN796932 | MH573513 | KT379533 | JQ901022 | KR187546 | OR521376 | MW953828 |
| MN800776 | MN800878 | MN426089 | MN796833 | KY244595 | KR823153 | JX679207 | KR188121 | MW957799 | MW953829 |
| MN801918 | MN800413 | MN426479 | MN796768 | MH573468 | KR823139 | JQ898256 | KR188022 | MW957932 | MW953973 |
| MN800754 | MN800326 | MN425988 | MN796626 | KY244586 | KR823017 | JQ898252 | KR187522 | MW957942 | MW955282 |
| MN802000 | MN800111 | MK458992 | MN796625 | MH028362 | KR823016 | JQ898247 | KR187433 | MW957768 | MW954039 |
| MN800750 | MN800086 | MK459033 | MN799055 | MH028335 | KR823030 | JQ302741 | KR188100 | OR521742 | MW957623 |
| MN802998 | MT590104 | MN426288 | MN796616 | MF037061 | KR823015 | JQ302740 | KR188068 | OR521677 | MW957337 |
| MN800749 | MT589666 | MK409792 | MN797029 | MF037037 | KR823007 | JQ302738 | KR188009 | OR521665 | MW957562 |
| MN802640 | MN802875 | MN426590 | MN796606 | KY244577 | KR823900 | JQ302737 | KR187660 | OR521664 | MW957413 |
| MN800741 | MN800873 | MN426103 | MN797169 | MF037163 | KR823131 | JQ302719 | KR187430 | OP831139 | MW957409 |
| MN801491 | MN802997 | MK459217 | MN796805 | KY244563 | KR823043 | JQ302718 | KR187683 | OP831115 | MW957481 |
| MN800728 | MN801144 | MN336588 | MN796603 | MF037114 | KR822999 | JQ302707 | KR187423 | OP831123 | MW957368 |
| MN801703 | MN800354 | MN426345 | MN799666 | KY244558 | KR822996 | JQ302726 | KR187732 | OP831099 | MW957370 |
| MN801558 | MT589706 | MN426589 | MN798097 | MH010691 | KR823003 | JQ302704 | KR187671 | OP831090 | MW957345 |
| MN800716 | MT590152 | MN426567 | MN798081 | KY713531 | KR823009 | JQ302657 | KR187421 | OP831089 | MW957335 |
| MN802312 | MT589853 | MN426512 | MN797640 | MK286984 | KR822994 | JQ302656 | KR187725 | OP831045 | MW953725 |
| MN802185 | MN800256 | MN425989 | MN797446 | MH573175 | KT379703 | JQ302653 | KR187420 | OP831097 | MW952568 |
| MN801394 | MT590012 | MK458991 | MN797203 | MH632387 | KT379292 | JQ302655 | KR187419 | OP831016 | MW953247 |
| MN800717 | MN801754 | MN601651 | MN797145 | MH300243 | KT379179 | JQ302644 | KR188196 | OP830976 | MW953798 |
| MN800702 | MT590040 | MN426959 | MN797125 | MH300225 | KT378688 | JQ302638 | KR187829 | OP830975 | MW954081 |
| MN801619 | MT589774 | MN426767 | MN797000 | MH028276 | KT379802 | JQ302627 | KR187445 | OP830914 | MW954754 |
| MN800685 | MT986426 | MN426496 | MN796767 | MF037120 | KT379230 | JQ302626 | KR188046 | OR522437 | MW955480 |
| MN800847 | MN801139 | MN426472 | MN796679 | MH632351 | KT378966 | JQ302625 | KR187791 | OR522427 | MW957093 |
| MN800670 | MN800820 | MN426327 | MN796662 | MG706713 | KT378781 | JQ302712 | KR187456 | OR521372 | MW952369 |
| MN801162 | MT986182 | MN426194 | MN796854 | MH789893 | KT378766 | JQ302620 | KR187416 | OR522420 | MW952354 |
| MN800667 | MT986073 | MN426185 | MN799379 | MH789867 | KR822970 | JQ302636 | KR187563 | OR521425 | MW955566 |
| MN801522 | MN802180 | MN426151 | MN798718 | MH632597 | KX583276 | JQ302608 | KR187413 | OR522403 | MW954229 |
| MN800665 | MT590002 | MN426102 | MN797893 | MH300168 | KX791593 | JQ302635 | KR187554 | OR522402 | MW957632 |
| MN801474 | MN801238 | MN426081 | MN797746 | MH300150 | KX791535 | JQ302580 | KR187408 | OR522398 | MW956751 |
| MN800658 | MN800997 | MK543569 | MN797258 | MG706849 | KR822966 | JQ302706 | KR187604 | OR522393 | MW954182 |
| MN800654 | MN800743 | KC203214 | MN796904 | MF941325 | KR823730 | JQ302571 | KR187407 | OR522406 | MW955225 |
| MN800707 | MN799962 | MN426901 | MN796896 | MF684120 | KR823021 | JQ028392 | KR187658 | OR522377 | MW954171 |
| MN800637 | MT589702 | MN426834 | MN796596 | MF684111 | KR823013 | JQ028252 | KR187492 | OR521740 | MW954635 |
| MN801785 | MN654108 | MN426652 | MN797080 | MF684068 | KR823002 | JQ028347 | KR187381 | OR522371 | MW955215 |
| MN800629 | MN654106 | MN426544 | MN797881 | KY713513 | KR822967 | JQ028230 | KR187491 | OR522354 | MW953236 |
| MN801430 | MN427297 | MN426532 | MN797847 | MH573310 | KR822964 | JQ302754 | KR187380 | OR522352 | MW953581 |
| MN800627 | MN427246 | MN426447 | MN799745 | MG450629 | KR823471 | JQ028205 | KJ401742 | OR522335 | MW953544 |
| MN802903 | MN427269 | MN426325 | MN797730 | MH300383 | KR822992 | JQ302572 | KR187375 | OR522331 | MW953492 |
| MN800851 | MN427225 | MN426189 | MN797813 | MF941347 | KR822988 | JQ028203 | KR188185 | OR522350 | MW953491 |
| MN800623 | MN427270 | MN426167 | MN797555 | KY713419 | KR822960 | JF340054 | KR188018 | OR522309 | MW953527 |
| MN802520 | MN427209 | MN426094 | MN797305 | MH300406 | KU954598 | JF313463 | KR187885 | OR522300 | MW953481 |
| MN800619 | MN427357 | MN426058 | MN796876 | MH573289 | KU364401 | HG421730 | KR187482 | OR522298 | MW953914 |
| MN800606 | MN427200 | MN426038 | MN796826 | MG450642 | KU364386 | HG421729 | KR187524 | OR522283 | MW954819 |
| MN801097 | MN427260 | MN425994 | MN799581 | MF037093 | KU050548 | HG421734 | KR187523 | OR522282 | MW953429 |
| MN800599 | MN427285 | MN425923 | MN633788 | MH632364 | KU050302 | HG421719 | KR187538 | OR522277 | MW953388 |
| MN800594 | MN427261 | MN425853 | MK771305 | MH300159 | KT379861 | HG421664 | KR188001 | OR522245 | MW952529 |
| MN802206 | MN427191 | MN601672 | MK771197 | MH028377 | KT379795 | HG421663 | KR187560 | OR522229 | MW953362 |
| MN800590 | MN427266 | MK287284 | MN797633 | MG894022 | KT379779 | HG421651 | KR187357 | OR522231 | MW953532 |
| MN800586 | MN427185 | MN425588 | MN797421 | MG706958 | KT379755 | HG421683 | KR187463 | OR522337 | MW953344 |
| MN800584 | MN427264 | MN425184 | MK771264 | MG706867 | KT379682 | HG421637 | KR187349 | OR522227 | MW952431 |
| MN801809 | MN427169 | MN426814 | MN797160 | MG706857 | KT379642 | HG421634 | KR187452 | OR522409 | MW953453 |
| MN801684 | MN427234 | MN426352 | MN799303 | MG706729 | KT379478 | HG421632 | KR187383 | OR522332 | MW953342 |
| MN800559 | MN427179 | MN426029 | MN796695 | MG706625 | KT379460 | HG421622 | KR187346 | OR522327 | MW953296 |
| MN800896 | MN427197 | MK459022 | MN799155 | MG706588 | KT379415 | HG421624 | KR187996 | OR522243 | MW953288 |
| MN801705 | MN427152 | MN425545 | MN633832 | MG706472 | KT379277 | HG421621 | KR187460 | OR522287 | MW954884 |
| MN800546 | MN427182 | MN426219 | MN796727 | MF684245 | KT379194 | HG421639 | KR187343 | OR522218 | MW954589 |
| MN801655 | MN427157 | MK543664 | MK948110 | MF684229 | KT379105 | HG421594 | KR187536 | OR522214 | MW953266 |
| MN800540 | MN427150 | MK543613 | MN633551 | MF684228 | KR823824 | HG421721 | KR187388 | OR522182 | MW953198 |
| MN802269 | MN427188 | MK459263 | MN799363 | MF684214 | KR823811 | HG421592 | KR187339 | OR522180 | MW953712 |
| MN800536 | MN427161 | MN425806 | MN798525 | MF684206 | KT379078 | HG421628 | KR187443 | OR522401 | MW953183 |
| MN800967 | MN427174 | MK543605 | MN798039 | MF684194 | KR823840 | HG421587 | KR187331 | OR522294 | MW952400 |
| MN800527 | MN427136 | MN601507 | MN797954 | MF684189 | KY226198 | HG421646 | KR187369 | OR522183 | MW952605 |
| MN802643 | MN427323 | MK459173 | MN797774 | MF684169 | KY226161 | HG421564 | KR187328 | OR522177 | MW952351 |
| MN800513 | MN427306 | MN426917 | MN797327 | MF684150 | KY226156 | HG421710 | KR187326 | OR522170 | MW953162 |
| MN801546 | MN427120 | MN426449 | MN797146 | MF684137 | KY226047 | HG421551 | KR187863 | OR522169 | MW953145 |
| MN800500 | MN427119 | MN426174 | MN796724 | MF684136 | KU954691 | HG421550 | KR187323 | OR522458 | MW951964 |
| MN802571 | MN427340 | MN426079 | MN633922 | MF684129 | KU954679 | HG421580 | KM011668 | OR522441 | MW953084 |
| MN800650 | MN427110 | MN426735 | MN633667 | MF684124 | KU871416 | HG421523 | KR187315 | OR522162 | MW953059 |
| MN800498 | MN427345 | MN426934 | MN462752 | MF684072 | KU364406 | HG421722 | KR187312 | OR521384 | MW953058 |
| MN801413 | MN427359 | MN426792 | MN799571 | KY713450 | KU364403 | HG421553 | KR187308 | OR522127 | MW953682 |
| MN800489 | MN427189 | MN426599 | MN799139 | KY713428 | KU364394 | HG421516 | KR187305 | OR522256 | MW953052 |
| MN802097 | MN427094 | MN426278 | MK771190 | KY244768 | KU364389 | HG421716 | KR187304 | OR522119 | MW957443 |
| MN800488 | MN427127 | MN426220 | MN797587 | MH632664 | KU364387 | HG421715 | KR187364 | OR521687 | MW952964 |
| MN801225 | MN427180 | MN426162 | MK771325 | MH632611 | KU050527 | HG421588 | KR187303 | OR522117 | MW952958 |
| MN800487 | MN427116 | MN426054 | MN633684 | MH632562 | KU050514 | HG421505 | KR187295 | OR522115 | MW952483 |
| MN800742 | MN427084 | MN426047 | MN633539 | MH632513 | KU050409 | HG421504 | KR187454 | OR522106 | MW952909 |
| MN800485 | MN427083 | MN426960 | MN633471 | MH632497 | KU050358 | HG421596 | KR187290 | OR522293 | MW953351 |
| MN801358 | MN427085 | MN426947 | MN633541 | MH632492 | KU050305 | HG421501 | KR187650 | OR522292 | MW952898 |
| MN801759 | MN427065 | MN426946 | MN633671 | MH632479 | KU050260 | HG421490 | KR187521 | OR522071 | MW952645 |
| MN800467 | MN427172 | MN426928 | MN633785 | MH632476 | KU050233 | HG421735 | KR187282 | OR522319 | MW952853 |
| MN800464 | MN427078 | MN426927 | MN633783 | MH632446 | KT379957 | HG421733 | KR187281 | OR522031 | MW955015 |
| MN801999 | MN427364 | MN426882 | MN633619 | MH632371 | KT379946 | HG421549 | KJ401628 | OR521733 | MW952821 |
| MN800461 | MN427362 | MN426851 | MN633689 | MH300375 | KT379945 | HG421478 | KR187273 | OR522383 | MW951928 |
| MN800454 | MN427351 | MN426820 | MK948104 | MH300172 | KT379935 | HG421476 | KR187437 | OR522012 | MW957019 |
| MN800442 | MN427102 | MN426645 | MN633819 | MG706973 | KT379909 | HG421465 | KR187260 | OR521701 | MW951902 |
| MN801625 | MN427044 | MN426608 | MN633764 | MG706969 | KT379904 | HE591065 | KR187556 | OR522415 | MW951809 |
| MN800437 | MN427041 | MN426605 | MN633463 | MG706768 | KT379903 | HE591038 | KR187241 | OR522007 | MW953371 |
| MN802589 | MN427163 | MN426426 | MN462810 | MG706722 | KT379897 | HE591027 | KR187299 | OR521676 | MW953459 |
| MN800432 | MN427194 | MN426403 | MN799712 | MF941489 | KT379894 | HE591026 | KR187240 | OR521675 | MW951780 |
| MN801109 | MN427126 | MN426380 | MN799592 | MF941423 | KT379889 | HE591063 | KR187813 | OR521674 | MW954840 |
| MN801928 | MN427082 | MN426353 | MN799570 | MF941316 | KT379869 | HE591015 | KR187239 | OR521979 | MW951755 |
| MN800428 | MN427283 | MN426329 | MN799563 | MF941260 | KT379868 | HE591061 | KR187457 | OR521974 | MW952245 |
| MN801813 | MN427240 | MN426305 | MN799547 | MF941272 | KT379805 | HE590985 | KR187230 | OR521970 | MW956634 |
| MN800426 | MN427042 | MN426236 | MN799525 | KY713433 | KT379607 | HE590990 | KR187316 | OR522033 | MW955265 |
| MN801776 | MN427024 | MN426183 | MN799506 | MH632694 | KT379583 | HE590973 | KR187223 | OR521963 | MW956380 |
| MN800404 | MN427045 | MN426176 | MN799408 | MH632610 | KT379575 | HE591060 | KR187924 | OR521962 | MW954740 |
| MN800710 | MN427173 | MN426113 | MN799354 | MH632582 | KT379573 | HE590970 | KR187676 | OR521961 | MW953142 |
| MN800388 | MN427023 | MN426002 | MN799342 | MF941491 | KT379532 | HE591059 | KR187210 | OR522394 | MW954963 |
| MN802118 | MN427017 | MN426000 | MN799307 | MH028353 | KT379500 | HE590969 | KR188211 | OR522125 | MW956220 |
| MN800382 | MN427014 | MN425997 | MN799293 | MH028341 | KT379336 | HE591058 | KR187204 | OR522081 | MW953918 |
| MN802697 | MN427010 | MN425958 | MN799245 | MF037111 | KT379333 | HE590956 | KR187264 | OR522187 | MW956205 |
| MN802149 | MN427009 | MN425888 | MN799090 | MH921154 | KT379024 | HE591057 | KR187598 | OR521924 | MW954346 |
| MN801837 | MN426986 | MN426319 | MN799076 | MH921151 | KT378979 | HE590948 | KR187196 | OR521922 | MW956171 |
| MN801265 | MN426983 | MN601650 | MN799071 | MH921132 | KT378935 | HE591056 | KR187919 | OR521921 | MW954215 |
| MN801211 | MN426900 | MN601649 | MN799045 | MH921099 | KT378929 | HE590946 | KR187917 | OR521917 | MW954475 |
| MN801159 | MN426941 | MN601643 | MN799007 | MH632663 | KT378896 | HE591055 | KR187876 | OR522094 | MW954231 |
| MN801035 | MN426832 | MK459200 | MN798981 | MH632506 | KT378895 | HE590940 | KR187752 | OR522076 | MW954258 |
| MN800719 | MN426809 | MK948151 | MN798929 | MH632487 | KT378887 | HE591000 | KR187539 | OR521912 | MW956082 |
| MN800706 | MN426800 | MN426502 | MN798890 | MH632412 | KT378855 | HE590929 | KR187489 | OR522225 | MW954389 |
| MN800377 | MN426799 | MN426226 | MN798743 | MH573534 | KT378848 | HE591053 | KR187426 | OR521890 | MW954498 |
| MN800587 | MN426706 | MN426109 | MN798634 | MH573520 | KT378838 | HE590917 | KR187327 | OR521889 | MW953814 |
| MN800374 | MN426680 | MK458973 | MN798600 | MH573508 | KT378827 | HE591054 | KR187248 | OR521868 | MW954979 |
| MN800402 | MN426668 | MK459086 | MN798515 | MH573224 | KT378824 | HE591052 | KR187199 | OR521864 | MW953788 |
| MN800365 | MN426666 | MK459041 | MN798466 | MH573218 | KT378812 | HE590914 | KR106896 | OR522362 | MW955522 |
| MN802003 | MN426667 | MN425165 | MN798375 | MH573202 | KT378805 | HE590913 | KR106888 | OR522178 | MW956043 |
| MN800338 | MN426664 | MN426916 | MN798274 | MH300387 | KT378800 | HE590911 | KR106872 | OR522157 | MW954655 |
| MN800389 | MN426711 | MN426886 | MN798133 | MH300372 | KT378792 | HE591051 | KR106938 | OR521923 | MW956038 |
| MN800816 | MN426695 | MN426885 | MN798123 | MH300336 | KT378788 | HE590910 | KR106871 | OR521853 | MW954916 |
| MN800330 | MN426622 | MN426818 | MN798105 | MH300324 | KT378782 | HE591050 | KR106907 | OR521865 | MW956020 |
| MN802900 | MN426675 | MN426541 | MN798069 | MH300263 | KT378779 | HE590909 | KR106863 | OR521847 | MW954119 |
| MN802068 | MN426529 | MN426425 | MN798056 | MH300178 | KT378719 | HE591049 | KR106899 | OR521881 | MW955880 |
| MN800328 | MN426530 | MN426356 | MN797958 | MH300146 | KR823960 | HE590908 | KR106844 | OR521837 | MW956366 |
| MN800322 | MN426612 | MN426210 | MN797889 | MH028365 | KR823452 | HE591047 | KR106852 | OR521407 | MW954968 |
| MT368472 | MN426508 | MN426100 | MN797877 | MH028284 | KR823473 | HE590905 | KR106838 | OR521737 | MW955803 |
| MN802045 | MN426580 | MN426090 | MN797842 | MH028266 | KR824024 | HE590901 | KR106885 | OR522297 | MW957507 |
| MN801508 | MN426504 | MN426084 | MN797812 | MH010670 | KX583340 | HE591046 | KR106845 | OR522286 | MW955341 |
| MN800312 | MN426408 | MN426068 | MN797798 | MG706931 | KT379691 | HE590899 | KR106837 | OR522281 | MW955743 |
| MN800332 | MN426781 | MN426064 | MN797783 | MG706915 | KU050518 | HE591045 | KR106831 | OR522268 | MW955728 |
| MN800468 | MN426397 | MN426036 | MN797782 | MG706913 | KU050516 | HE590894 | KR106834 | OR522261 | MW955708 |
| MN800306 | MN426592 | MN425966 | MN797727 | MG706894 | KT379044 | HE591044 | KR106824 | OR521882 | MW955574 |
| MN802031 | MN426514 | MN425963 | MN797724 | MG706753 | KR823807 | HE590893 | KR106859 | OR521836 | MW954823 |
| MN802850 | MN426394 | MN425920 | MN797699 | MG706752 | KR823280 | GU345416 | KR106823 | OR522423 | MW952326 |
| MN800819 | MN426731 | MN425895 | MN797583 | MG706656 | KX791620 | GU345392 | KR106814 | OR522078 | MW953349 |
| MN800298 | MN426719 | MK543555 | MN797544 | MG706586 | KX791572 | GU345705 | KR106813 | OR521822 | MW953301 |
| MN801370 | MN426515 | MK458957 | MN797481 | MF941480 | KX791508 | GU345387 | KR106812 | OR521935 | MW952907 |
| MN800297 | MN426649 | MN426531 | MN797472 | MF941414 | KT379229 | GU345697 | KR106811 | OR521821 | MW952263 |
| MN800789 | MN426686 | MN426331 | MN797328 | MF325061 | KR823326 | GU345362 | KR106878 | OR521819 | MW952719 |
| MN800289 | MN426473 | MK459264 | MN797310 | MF037175 | KR823655 | GU345337 | KR106810 | OR521814 | MW952141 |
| MN801613 | MN426457 | MK459268 | MN797159 | MF037173 | KR823930 | HQ198023 | KR106779 | OR521807 | MW951753 |
| MN800286 | MN426385 | MN426540 | MN797101 | MF037171 | KY226045 | GU328958 | KR106775 | OR522274 | MW951911 |
| MN800572 | MN426365 | MN427027 | MN796970 | MF037170 | KX583285 | GU328887 | KR106804 | OR521806 | MW951749 |
| MN800280 | MN426363 | MN427025 | MN796927 | MF037108 | KX583272 | GU328951 | KR106772 | OR521392 | MW951676 |
| MN802537 | MN426551 | MK892945 | MN796874 | MF037031 | KU364405 | GU328880 | KP992441 | OR521390 | MW951675 |
| MN802936 | MN426484 | MN426999 | MN796800 | KY713524 | KU050621 | GU345296 | KP992438 | OR521773 | MW955416 |
| MN800260 | MN426349 | MN426930 | MN796780 | KY713523 | KU050618 | GU328915 | KP992422 | OR521395 | MW953143 |
| MN802006 | MN426533 | MN426791 | MN796769 | KY713436 | KU050613 | GU328879 | KP992420 | OR522310 | MW956413 |
| MN801601 | MN426335 | MN426518 | MN796707 | KY713398 | KU050599 | GU345431 | KP992410 | OR522295 | MW955736 |
| MN801596 | MN426575 | MN426498 | MN796701 | KY244564 | KU050581 | GU345419 | KP992409 | OR522137 | MW956243 |
| MN800258 | MN426311 | MN426360 | MN796623 | MF941433 | KU050498 | GU345401 | KR188358 | OR521770 | MW955964 |
| MN802490 | MN426681 | MN426235 | MN796617 | MH010688 | KU050487 | GU345393 | KR188357 | OR521713 | MW954859 |
| MN800333 | MN426310 | MN426227 | MN796600 | MH921078 | KU050486 | GU328875 | KR188236 | OR521712 | MW955677 |
| MN800254 | MN426753 | MN426187 | MN633920 | MH632686 | KU050482 | GU345244 | KR188109 | OR521984 | MW955442 |
| MN802834 | MN426467 | MN426180 | MN633591 | MH632443 | KU050478 | GU328873 | KR187861 | OR521795 | MW955028 |
| MN801569 | MN426361 | MN426170 | MN633578 | MH632397 | KU050477 | GU328945 | KR187742 | OR521792 | MW955031 |
| MN800253 | MN426309 | MN426073 | MK771322 | MH300297 | KU050461 | GU328864 | KR187677 | OP329634 | MW954668 |
| MN802382 | MN426308 | MN425883 | MK771175 | MH028331 | KU050454 | GU328862 | KP992397 | OP191568 | MW954817 |
| MN800251 | MN426810 | MN426107 | MN799724 | MH028268 | KU050448 | GU328861 | KP992395 | OP191567 | MW957577 |
| MN802271 | MN426299 | MN425938 | MN799681 | MF037158 | KU050447 | GU328842 | KP992393 | OP329547 | MW957511 |
| MN800645 | MN426298 | MN426853 | MN799591 | MF037081 | KU050439 | GU328835 | KR188103 | OP191489 | MW957295 |
| MN800236 | MN426297 | MN426573 | MN799497 | MF037060 | KU050430 | GU328834 | KP992392 | OP191413 | MW957495 |
| MN802723 | MN426386 | MN426549 | MN799403 | KY713504 | KU050428 | GU328807 | KP992387 | OP191412 | MW956978 |
| MN802448 | MN426249 | MN426460 | MN799294 | MH632405 | KU050406 | GU328836 | KR187854 | OP191277 | MW957267 |
| MN800231 | MN426463 | MN426423 | MN799227 | MF037140 | KU050397 | GU328828 | KR187532 | OP191275 | MW957069 |
| MN800518 | MN426247 | MN426302 | MN799091 | MH632427 | KU050384 | GU328802 | KP992384 | OP191427 | MW957393 |
| MN800230 | MN426650 | MN426211 | MN799022 | KY244741 | KU050380 | GU328829 | KR188403 | OP191245 | MW953554 |
| MN800229 | MN426376 | MN426199 | MN798845 | MH010710 | KU050377 | GU328803 | KR188348 | OP191060 | MW956138 |
| MN801871 | MN426240 | MN426143 | MN798740 | MH632564 | KU050362 | GU328801 | KR188175 | OP191059 | MW956602 |
| MN800228 | MN426587 | MN426136 | MN798732 | MH632367 | KU050329 | GU328799 | KR188172 | OP190992 | MW957474 |
| MN800827 | MN426351 | MN426110 | MN798661 | MH632362 | KU050314 | GU328798 | KR188099 | OP190916 | MW957088 |
| MN800222 | MN426350 | MN426101 | MN798657 | MF941246 | KU050278 | GU345712 | KP992381 | OP190893 | MW956420 |
| MN802756 | MN426237 | MN426095 | MN798545 | MF941237 | KU050243 | GU328846 | KP992434 | OP190883 | MW957537 |
| MN801853 | MN426317 | MN426092 | MN798442 | MF941232 | KU050229 | GU328845 | KP992362 | OP191074 | MW956499 |
| MN800216 | MN426248 | MN426085 | MN798292 | KY713493 | KU050219 | GU328844 | KR188327 | OP190922 | MW956291 |
| MN800745 | MN426233 | MN426082 | MN798111 | KY713555 | KT379908 | GU328843 | KP992348 | OP190870 | MW954671 |
| MN800209 | MN426929 | MN426060 | MN797886 | MH632498 | KT379737 | GU328841 | KP992344 | OP190726 | MW957095 |
| MN802116 | MN426924 | MN426059 | MN797864 | MG706796 | KT379693 | GU328840 | KP992343 | OP190721 | MW956189 |
| MN801586 | MN426594 | MN426037 | MN797762 | MH632646 | KT379611 | GU328839 | KR187664 | OP190505 | MW953082 |
| MN800766 | MN426556 | MN425935 | MN796947 | MF941244 | KT379585 | GU328838 | KC888744 | OP190649 | MW956132 |
| MN800202 | MN426554 | MN601644 | MN796722 | MH300355 | KT379481 | GU328837 | KR188319 | OP190474 | MW954381 |
| MN801576 | MN426468 | MK459195 | MN633881 | MG706503 | KT379467 | GU328833 | KR187427 | OP191564 | MW955829 |
| MN800421 | MN426244 | MK459244 | MN633883 | MF941312 | KT379429 | GU328832 | KC888731 | OP191561 | MW955828 |
| MN800189 | MN426232 | MN426890 | MN797069 | MF684102 | KT379305 | GU328831 | KC888738 | OP191287 | MW954631 |
| MN800457 | MN426224 | MN426878 | MN796709 | MH632668 | KT379266 | GU328830 | KC888736 | OP191398 | MW955494 |
| MN800185 | MN426384 | MN426819 | MK771213 | KY713427 | KT379163 | GU328827 | KC888728 | OP329680 | MW955840 |
| MN801787 | MN426231 | MN426777 | MK771168 | MH632604 | KT379161 | GU328826 | KC888720 | OP191175 | MW955406 |
| MN800179 | MN426222 | MN426765 | MN797749 | MH300359 | KT379150 | GU328825 | KC888719 | OP191193 | MW957339 |
| MN800165 | MN426696 | MN426584 | MN798870 | KY713455 | KT379142 | GU328824 | KC888687 | OP191174 | MW957336 |
| MN800210 | MN426501 | MN426569 | MN798218 | MF037133 | KT379093 | GU328822 | KC888684 | OP191076 | MW955550 |
| MN800162 | MN426221 | MN426489 | MN797817 | KY226218 | KT379089 | GU328821 | KC888705 | OP191065 | MW955069 |
| MN803094 | MN426371 | MN426485 | MN797701 | MK286990 | KT379029 | GU328820 | KC888671 | OP191056 | MW955547 |
| MN800155 | MN426364 | MN426375 | MN633769 | MK286969 | KT379020 | GU328819 | KF857428 | OP190986 | MW954234 |
| MN801095 | MN426155 | MN426370 | MK771269 | MH632378 | KT378978 | GU328818 | KF857397 | OP191011 | MW956157 |
| MN800152 | MN426414 | MN426250 | MK771262 | MH632648 | KT378972 | GU328817 | KF857369 | OP191179 | MW954184 |
| MN800226 | MN426368 | MN426209 | MK771186 | MH632558 | KT378971 | GU328816 | KF857368 | OP190957 | MW955046 |
| MN800346 | MN426216 | MN426196 | MK771178 | MH632538 | KT378912 | GU328815 | KF857365 | OP190881 | MW954051 |
| MN800145 | MN426948 | MN426178 | MN799689 | MH632507 | KT378883 | GU328814 | KF857358 | OP190942 | MW954025 |
| MN800130 | MN426124 | MN426150 | MN799586 | MG706592 | KT378860 | GU328813 | KC888662 | OP329548 | MW954026 |
| MN802002 | MN426171 | MN426148 | MN799580 | MG706550 | KT378821 | GU328812 | KC888654 | OP190933 | MW952816 |
| MN801658 | MN426971 | MN426135 | MN799496 | KY713567 | KT378764 | GU328810 | KR188048 | OP190591 | MW952812 |
| MN800107 | MN426591 | MN426105 | MN799450 | MH632636 | KT378714 | GU328806 | KR187892 | OP190601 | MW952962 |
| MN802800 | MN426114 | MN426063 | MN799365 | MG706938 | KT378649 | GU328805 | KC888647 | OP190553 | MW952900 |
| MN800106 | MN426435 | MN425991 | MN799275 | MG706749 | KR823989 | GU328804 | KC888637 | OP190552 | MW952794 |
| MN802042 | MN426931 | MN425980 | MN799212 | MG706458 | KR823955 | GU328800 | KC888640 | OP329422 | MW952906 |
| MN800105 | MN426574 | MN425979 | MN799174 | MG706457 | KR823932 | GU328797 | KC888634 | OP329488 | MW952742 |
| MN801273 | MN426091 | MN425939 | MN799075 | MF941418 | KR823898 | GU328796 | KC888643 | OP190523 | MW952230 |
| MN800104 | MN426458 | MN425927 | MN798970 | KY713527 | KR823844 | GU328792 | KR187756 | OP329423 | MW952112 |
| MN802358 | MN426388 | MN425919 | MN798922 | MH789870 | KR823802 | GU328791 | KR187578 | OP329489 | MW952087 |
| MN800708 | MN426215 | MN425897 | MN798887 | MH300227 | KR823779 | GU328790 | KR187558 | OP190515 | MW952384 |
| MN801561 | MN426083 | MN425882 | MN798865 | MH300216 | KR823777 | GU328789 | KC888635 | OP329660 | MW957427 |
| MN800101 | MN426372 | MN425881 | MN798790 | MH300143 | KR823765 | GU328787 | KC888633 | OP329582 | MW956203 |
| MN800100 | MN426246 | MK287163 | MN798662 | MG706755 | KR823749 | GU328786 | KC888628 | OP329657 | MW957348 |
| MN801799 | MN426895 | MN426938 | MN798648 | MG706512 | KR823684 | GU328785 | KR187301 | OP329579 | MW954750 |
| MN800098 | MN426057 | MN426867 | MN798561 | MG706506 | KR823679 | GU328784 | KC888622 | OP329642 | MW957297 |
| MN800478 | MN426922 | MN425928 | MN798486 | MG706479 | KR823624 | GU328783 | KC888609 | OP329564 | MW952690 |
| MN800092 | MN426172 | MN425884 | MN798485 | MF037074 | KR823548 | GU328782 | KC888608 | OP190562 | MW957232 |
| MN801583 | MN426041 | MN426213 | MN798483 | MF037048 | KR823474 | GU328781 | KJ401653 | OP329552 | MW957008 |
| MN800085 | MN426369 | MN426065 | MN798440 | KY713579 | KR823292 | GU328780 | KF914624 | OP191338 | MW955594 |
| MN802805 | MN426332 | MN601666 | MN798431 | KY713404 | KR823186 | GU328779 | KC888625 | OP329542 | MW957070 |
| MN800746 | MN426429 | MN427337 | MN798424 | KY713370 | KR823161 | GU328778 | KC888581 | OP191212 | MW954648 |
| MN800083 | MN426241 | MN427329 | MN798255 | KY226221 | KR822998 | GU328777 | KJ401755 | OP329525 | MW956995 |
| MN800163 | MN425976 | MN427145 | MN798224 | KY972174 | KY226115 | GU328776 | KC888580 | OP190695 | MW956893 |
| MN800077 | MN425978 | MK892883 | MN798199 | MG706552 | KU954728 | GU328775 | KP992382 | OP329523 | MW956541 |
| MN802076 | MN426445 | MK459234 | MN798125 | MH573215 | KU954721 | GU328774 | KC888579 | OP191519 | MW956765 |
| MN800075 | MN426072 | MK543583 | MN798052 | MH010713 | KU954644 | GU328773 | KR187805 | OP329512 | MW956441 |
| MN800207 | MN425973 | MN427133 | MN797888 | MH010696 | KU954581 | GU328772 | KC888577 | OP191359 | MW956425 |
| MN800074 | MN426116 | MN426677 | MN797865 | MH632653 | KU954577 | GU328771 | KC888619 | OP329511 | MW956353 |
| MN802952 | MN425960 | MN424807 | MN797818 | MF684079 | KT379885 | GU328770 | KR187883 | OP190550 | MW956412 |
| MN802052 | MN425959 | MN426766 | MN797772 | MH632699 | KT379530 | GU328769 | KR187669 | OP329510 | MW953498 |
| MN801499 | MN425926 | MK543686 | MN797757 | MH028292 | KT379439 | GU328768 | KC888562 | OP191549 | MW956398 |
| MN800053 | MN425981 | MK459179 | MN797738 | MG706954 | KT379136 | GU328767 | KC888611 | OP329509 | MW954763 |
| MN800419 | MN425983 | MK459003 | MN797604 | MG706594 | KR823718 | GU328766 | KC888546 | OP191331 | MW954875 |
| MN800233 | MN425823 | MN426866 | MN797535 | MF941401 | KR823580 | GU328765 | KC888596 | OP329508 | MW956095 |
| MN801384 | MN425816 | MN426855 | MN797455 | MF941394 | KR823267 | GU328764 | KC888538 | OP190544 | MW955978 |
| MN800050 | MN425707 | MN426737 | MN797427 | MF941389 | KR823004 | GU328763 | KR187966 | OP329507 | MW956068 |
| MN800048 | MN425706 | MN426655 | MN797299 | MF941366 | KX583301 | GU328762 | KR187755 | OP190792 | MW955452 |
| MN800019 | MN425734 | MN426306 | MN797282 | MH010673 | KT379121 | GU328761 | KR187714 | OP329505 | MW956042 |
| MN802456 | MN425694 | MN426197 | MN797206 | MF684141 | KR823163 | GU328760 | KR187565 | OP191499 | MW954305 |
| MN801340 | MN425698 | MN426181 | MN797117 | KY713347 | KY226171 | GU328759 | KR187531 | OP329504 | MW955968 |
| MN800010 | MN425678 | MN426160 | MN796964 | MH028279 | KU954718 | GU328758 | KR187505 | OP190943 | MW955894 |
| MN800596 | MN425650 | MN426075 | MN796917 | MG894025 | KU050551 | GU328757 | KR187496 | OP329503 | MW955915 |
| MN800009 | MN425648 | MN426061 | MN796891 | MF037169 | KT379924 | GU328755 | KR187338 | OP191588 | MW957346 |
| MN802236 | MN425653 | MN426056 | MN796877 | MH632700 | KT379883 | GU328754 | KR187313 | OP329502 | MW955877 |
| MN800008 | MN425647 | MN426052 | MN796873 | MH632579 | KT379880 | GU328753 | KR187310 | OP190530 | MW957067 |
| MN800054 | MN425597 | MN426049 | MN796738 | MH632491 | KT379754 | GU328752 | KR187236 | OP329501 | MW955865 |
| MN800002 | MN425596 | MN426044 | MN796721 | MH300427 | KT379286 | GU328751 | KC888571 | OP190527 | MW955959 |
| MN799997 | MN425521 | MN425985 | MN796705 | MH921165 | KT379257 | GU328750 | KC888576 | OP329500 | MW955861 |
| MN799995 | MN425511 | MN425982 | MN796684 | MH921079 | KT379026 | GU328749 | KC888529 | OP191311 | MW954360 |
| MN802161 | MN425514 | MN425965 | MN796665 | MH632708 | KT378993 | GU328748 | KC888617 | OP329499 | MW955853 |
| MN800422 | MN425510 | MN425936 | MK459191 | MH632602 | KT378976 | GU328747 | KC888520 | OP191545 | MW957565 |
| MN800243 | MN425494 | MN425893 | MN799620 | MH632526 | KT378951 | GU328746 | KC888543 | OP329498 | MW955826 |
| MN799988 | MN425493 | MN425891 | MN799367 | MH632475 | KT378877 | GU328729 | KC888509 | OP191308 | MW955441 |
| MN802210 | MN425552 | MN425886 | MN797826 | MH632422 | KT378847 | GU328741 | KP992388 | OP329497 | MW955786 |
| MN802004 | MN425487 | MN425880 | MN797689 | MH632373 | KT378780 | GU328714 | KC888595 | OP191307 | MW955732 |
| MN801333 | MN425523 | MK459170 | MN799043 | MH632372 | KT378747 | GU328728 | KC888505 | OP329496 | MW954532 |
| MN800937 | MN425465 | MK459089 | MN798653 | MH632326 | KT378657 | GU328717 | KC888531 | OP191306 | MW955499 |
| MN800431 | MN425458 | MK459020 | MN799691 | MH573525 | KY226137 | GU328710 | KC888527 | OP329495 | MW956331 |
| MN800325 | MN425457 | MN601668 | MN799548 | MH300237 | KY226125 | GU345711 | KR188292 | OP190791 | MW955561 |
| MN799984 | MN425454 | MN425967 | MN799522 | MH028369 | KY226066 | GU328795 | KC888539 | OP329494 | MW955528 |
| MT590060 | MN425451 | MN426218 | MN799499 | MH028359 | KX791549 | GU328794 | KC888485 | OP190520 | MW957126 |
| MN802496 | MN425557 | MN426307 | MN799200 | MH028358 | KX583328 | GU328793 | KP992358 | OP329493 | MW955523 |
| MN802140 | MN425448 | MN426106 | MN798407 | MH028316 | KU954740 | GU328744 | KC888732 | OP190789 | MW955510 |
| MN801811 | MN425434 | MN425970 | MN798383 | MH028306 | KU954707 | GU328743 | KC888481 | OP329492 | MW954949 |
| MN801540 | MN425433 | MN425961 | MN798171 | MH028291 | KU954694 | GU328742 | KC888514 | OP190516 | MW955463 |
| MN801447 | MN425394 | MN425887 | MN798150 | MH028282 | KU954659 | GU328740 | KC888477 | OP329490 | MW954420 |
| MN801287 | MN425471 | MN427059 | MN798109 | MH028269 | KU954647 | GU328738 | KR188440 | OP190976 | MW955422 |
| MN800420 | MN425386 | MN427057 | MN798077 | MH028259 | KU954634 | GU328736 | KR188202 | OP329551 | MW955447 |
| MN800181 | MN425374 | MN427035 | MN798059 | MG706852 | KU954633 | GU328734 | KR188088 | OP329485 | MW957574 |
| MN800144 | MN425368 | MK892920 | MN798050 | MG706818 | KU954623 | GU328733 | KR187826 | OP190578 | MW955431 |
| MN800021 | MN425571 | MK543677 | MN797697 | MG706803 | KU954619 | GU328732 | KC888569 | OP190573 | MW954728 |
| MN799982 | MN425432 | MK459157 | MN797596 | MG706595 | KU954608 | GU328731 | KC888545 | OP329545 | MW955426 |
| MN802773 | MN425355 | MN426697 | MN797496 | MG706480 | KU954585 | GU328730 | KC888475 | OP329479 | MW951878 |
| MN801285 | MN425384 | MN426357 | MN797402 | MF037156 | KU954580 | GU328727 | KC888525 | OP191632 | MW955358 |
| MN799975 | MN425350 | MN426572 | MN797113 | MF037134 | KU954576 | GU328726 | KC888508 | OP329544 | MW957606 |
| MN801038 | MN425503 | MK458951 | MN796982 | MF037125 | KU050321 | GU328725 | KC888492 | OP329478 | MW955267 |
| MN800102 | MN425392 | MN426975 | MN796956 | MF037044 | KT379937 | GU328722 | KC888473 | OP191106 | MW955879 |
| MN799974 | MN425589 | MN426961 | MN796860 | KY972198 | KT379932 | GU328721 | KP250698 | OP329540 | MW955211 |
| MN802044 | MN425308 | MN426006 | MN797367 | KY713487 | KT379920 | GU328720 | KC888533 | OP329474 | MW954485 |
| MN802005 | MN425326 | MN425962 | MK771266 | KY713465 | KT379916 | GU328719 | KC888469 | OP191103 | MW956247 |
| MN800160 | MN425305 | MN426846 | MN633772 | KY713438 | KT379915 | GU328718 | KC888530 | OP329473 | MW955200 |
| MN799971 | MN425276 | MN426783 | MN796803 | KY713409 | KT379859 | GU328716 | KC888467 | OP191102 | MW955193 |
| MN800247 | MN425275 | MN426734 | MN798571 | KY713383 | KT379844 | GU328715 | KC888550 | OP329472 | MW955180 |
| MN799970 | MN425515 | MN426601 | MN633468 | MH632689 | KT379829 | GU328713 | KC888465 | OP190712 | MW954441 |
| MN801360 | MN425266 | MN426505 | MN633454 | MH632630 | KT379822 | GU328709 | KR187581 | OP190710 | MW955131 |
| MN799964 | MN425314 | MN426069 | MN797506 | MH632353 | KT379803 | FM251984 | KR187360 | OP329537 | MW955072 |
| MN800045 | MN425264 | MN425987 | MN799436 | MH010712 | KT379801 | FM251982 | KR187271 | OP329471 | MW954082 |
| MN802770 | MN425226 | MN425984 | MN799269 | MH028381 | KT379777 | FM251976 | KC888453 | OP190534 | MW955679 |
| MN801289 | MN425293 | MN425898 | MN797855 | MH632333 | KT379776 | FM251974 | KR187637 | OP329549 | MW954896 |
| MN800551 | MN425211 | MN425885 | MN797186 | MH300260 | KT379746 | FM251973 | KR187277 | OR521736 | MW954622 |
| MN799935 | MN425205 | MK409842 | MN796878 | MH300152 | KT379692 | FM251967 | KR187543 | OP191612 | MW957107 |
| MN802796 | MN425390 | MN426206 | MN633858 | MH028267 | KT379664 | FM251966 | KC888451 | OP190595 | MW954623 |
| MN800146 | MN425369 | MN426190 | MN633915 | MF941477 | KT379643 | FM251963 | KR187251 | OP190579 | MW954394 |
| MN799930 | MN425204 | MN426139 | MN633696 | MF037135 | KT379615 | EF471865 | KC888449 | OP190568 | MW954330 |
| MN802257 | MN425199 | MN426074 | MK459015 | MF037036 | KT379613 | EF394231 | KR187252 | OP190525 | MW953892 |
| MN799928 | MN425196 | MN426007 | MN462813 | KY713441 | KT379600 | EF394226 | KC888448 | OP277913 | MW954080 |
| MN800024 | MN425285 | MN425993 | MN633859 | MH300280 | KT379597 | EF394225 | KR187275 | OP277912 | MW953747 |
| MN799927 | MN425401 | MN425986 | MN633833 | MH632385 | KT379554 | HQ198035 | KC888447 | OP277914 | MW953767 |
| MN801861 | MN425173 | MN425977 | MN799052 | MH573220 | KT379502 | EF420986 | KR187759 | OP277911 | MW953372 |
| MN800542 | MN425171 | MN425964 | MN799016 | MG706600 | KT379488 | EF368370 | KR187269 | OP277898 | MW953318 |
| MN799925 | MN425187 | MN425929 | MN633721 | MH300253 | KT379485 | EF036533 | KC888446 | OP277891 | MW953276 |
| MN802261 | MN425161 | MN425921 | MN633560 | KY713365 | KT379454 | EF036531 | KC888445 | OP277890 | MW953303 |
| MN799921 | MN425453 | MK459272 | MN633712 | MH632657 | KT379447 | JX112869 | KR187696 | OP277882 | MW952360 |
| MN801581 | MN425449 | MK459190 | MN798622 | MH300400 | KT379431 | DQ859178 | KR187261 | OP277881 | MW953350 |
| MN799920 | MN425348 | MN424810 | MN633896 | MH300346 | KT379412 | HQ198039 | KP992430 | OP277875 | MW951851 |
| MN802620 | MN425203 | MN633857 | MN798901 | MH632516 | KT379403 | HQ198036 | KR187250 | OP277874 | MW953212 |
| MN799919 | MN425147 | MN633855 | MN798165 | MH632383 | KT379387 | HQ198016 | KC888442 | OP277856 | MW952385 |
| MN801941 | MN425309 | MN633853 | MN799486 | MH300190 | KT379376 | HQ198015 | KR187587 | OP277855 | MW953150 |
| MN799992 | MN425186 | MN633841 | MN798938 | MG706847 | KT379317 | HQ198013 | KR187414 | OP277854 | MW953264 |
| MN800877 | MN425093 | MN633823 | MN798496 | MF037143 | KT379311 | EF394217 | KC888452 | OP277851 | MW952060 |
| MN799909 | MN425337 | MN633895 | MN797248 | MF037040 | KT379293 | DQ990880 | KC888440 | OP277850 | MW952407 |
| MN802263 | MN425319 | MN633851 | MN797075 | MH010700 | KT379287 | DQ833428 | KR187257 | OP277843 | MW957355 |
| MN799999 | MN425086 | MN633802 | MN798687 | MF037126 | KT379250 | DQ833416 | KC888439 | OP277842 | MW952380 |
| MN799907 | MN425222 | MN633817 | MN797957 | MF037109 | KT379210 | DQ833415 | KR188008 | OP277837 | MW952383 |
| MN801288 | MN425067 | MN633798 | MN797993 | KY713501 | KT379132 | DQ833429 | KR187670 | OP277836 | MW952330 |
| MN801065 | MN425335 | MN633808 | MN797448 | MF684103 | KT379113 | DQ833412 | KR187274 | ON412004 | MW952329 |
| MN800610 | MN425052 | MN633777 | MN797188 | MH028349 | KT379096 | DQ007903 | KR187270 | ON412003 | MW952328 |
| MN800482 | MN425559 | MN633834 | MK771258 | MH028256 | KT379057 | AY275556 | KR187218 | ON412000 | MW952333 |
| MN799987 | MN425334 | MN633774 | MN799265 | MF037032 | KT379013 | GU345708 | KC888455 | ON411999 | MW952199 |
| MN799904 | MN425041 | MN633761 | MN798044 | MF037190 | KT378995 | GU345694 | KC888434 | ON411997 | MW952100 |
| MN800368 | MN425540 | MN633760 | MN797878 | MF037098 | KT378992 | GU328952 | KP992380 | ON411998 | MW953503 |
| MN800173 | MN425409 | MN633839 | MN796651 | MH632697 | KT378965 | EF394228 | KC888502 | OK315309 | MW953599 |
| MN799892 | MN425366 | MN633756 | MN799409 | MH300396 | KT378927 | EF057102 | KC888432 | OK315270 | MW953598 |
| MN801728 | MN425356 | MN633715 | MN799315 | KY244698 | KT378902 | AY180905 | KR187220 | OK315190 | MW952999 |
| MN799889 | MN425273 | MN633714 | MN799605 | KY972211 | KT378890 | JF719819 | KC888416 | OK315186 | MW951708 |
| MN802211 | MN425269 | MN633862 | MN799470 | MG706909 | KT378888 | AY008716 | KC888613 | OK315153 | MW952476 |
| MN799887 | MN425232 | MN633656 | MN798505 | MH573523 | KT378871 | AY008715 | KC888544 | OK315159 | MW957589 |
| MN799885 | MN425221 | MN633821 | MN798493 | KY713469 | KT378868 | HG421491 | KC888406 | OK315151 | MW957198 |
| MN800515 | MN425210 | MN633638 | MN796622 | KY713451 | KT378813 | AM886209 | KC888391 | OK315150 | MW954467 |
| MN799882 | MN425176 | MN633632 | MN633923 | KY713368 | KT378746 | HG421635 | KC888386 | OK315179 | MW956706 |
| MN800561 | MN425143 | MN633631 | MN799242 | MG894035 | KT378708 | AM886195 | KC888365 | OK315148 | MW954983 |
| MN799881 | MN425138 | MN633650 | MN799186 | MF037152 | KT378703 | AM886180 | KC888336 | OK315114 | MW956212 |
| MN802073 | MN425057 | MN633607 | MN798537 | MH632434 | KR824027 | AM886179 | KC888417 | OK315102 | MW956078 |
| MN800613 | MN425562 | MN633806 | MN798387 | MF684064 | KR823965 | AB773885 | KC888318 | OK315076 | MW956528 |
| MN799870 | MN425189 | MN633605 | MN798379 | MH573205 | KR823866 | AB773884 | KR188284 | OK315030 | MW954590 |
| MN799993 | MN425020 | MN633610 | MN633852 | MH300302 | KR823843 | AB746343 | KJ401703 | OK315021 | MW956521 |
| MN799867 | MN425839 | MN633604 | MN633627 | MF684230 | KR823839 | AB746342 | KC888319 | OK315009 | MW956974 |
| MN800856 | MN425215 | MN633577 | MN633910 | MF684087 | KR823799 | DQ007901 | KC888317 | ON411873 | MW956710 |
| MN800078 | MN425175 | MN633576 | MK771268 | KY713565 | KR823789 | AY275557 | KC888312 | ON411872 | MW956486 |
| MN800016 | MN425095 | MN633600 | MK771195 | MH632350 | KR823761 | JQ028380 | KC888305 | ON411862 | MW952482 |
| MN799863 | MN425054 | MN633559 | MK771167 | MH921087 | KR823745 | JQ028411 | KC888297 | ON411861 | MW955024 |
| MN801493 | MN425043 | MN633546 | MN799670 | MH632662 | KR823733 | FJ387060 | KC888393 | OP329556 | MW953154 |
| MN799860 | MN425019 | MN633699 | MN799504 | MH632625 | KR823725 | FJ387054 | KC888295 | OP277897 | MW956476 |
| MN801573 | MN425265 | MN633697 | MN799503 | MH632521 | KR823717 | FJ387049 | KC888337 | OP277893 | MW956276 |
| MN801410 | MN425008 | MN633693 | MN799423 | MH632432 | KR823686 | JN848945 | KC888294 | OP277896 | MW956449 |
| MN799856 | MN425585 | MN633543 | MN799238 | MH632365 | KR823653 | JN848943 | KC888360 | OP277895 | MW952632 |
| MT589921 | MN425005 | MN633869 | MN798921 | MH300232 | KR823583 | JN848938 | KR187584 | OP277834 | MW956447 |
| MN799849 | MN425159 | MN633533 | MN798780 | MH300141 | KR823558 | JN848931 | KC888285 | OP277886 | MW955322 |
| MN801533 | MN425080 | MN633659 | MN798779 | MG706603 | KR823491 | JN848925 | KC888547 | OP277869 | MW956335 |
| MN799843 | MN425030 | MN633532 | MN798639 | MG706596 | KR823485 | JN848923 | KC888421 | OP277868 | MW957039 |
| MN802942 | MN425004 | MN633657 | MN798569 | MG706570 | KR823475 | HE591004 | KC888311 | OP277872 | MW956356 |
| MN802250 | MN424999 | MN633530 | MN798508 | MG706495 | KR823451 | JN848914 | KC888303 | OP277858 | MW955608 |
| MN801807 | MN424998 | MN633580 | MN798342 | MF941406 | KR823449 | JN848912 | KC888278 | OP277857 | MW956338 |
| MN801764 | MN425065 | MN633501 | MN798332 | MF941315 | KR823377 | JN848909 | KC888410 | OP277860 | MW955029 |
| MN801417 | MN424995 | MN633700 | MN798279 | MF037186 | KR823367 | JN848879 | KC888401 | ON411996 | MW954656 |
| MN800458 | MN425012 | MN633550 | MN798246 | KY713478 | KR823341 | JN848889 | KC888293 | ON411995 | MW956562 |
| MN800196 | MN424991 | MN633493 | MN798234 | MH632633 | KR823320 | JN848876 | KC888267 | ON412005 | MW955330 |
| MN800025 | MN425375 | MN633549 | MN798201 | MH632581 | KR823314 | JN848891 | KC888344 | ON412001 | MW956146 |
| MN800011 | MN425170 | MN633490 | MN798154 | MH632335 | KR823306 | JN848893 | KC888292 | ON411991 | MW954069 |
| MN801122 | MN425039 | MN633759 | MN798012 | MH573228 | KR823287 | JN848866 | KC888262 | ON411990 | MW955461 |
| MN799835 | MN424990 | MN633537 | MN797814 | MF037115 | KR823255 | JN848895 | KC888385 | ON411989 | MW957259 |
| MN802253 | MN425822 | MN633465 | MN797810 | KY244587 | KR823250 | JN848864 | KC888313 | ON411988 | MW956104 |
| MN801899 | MN424989 | MN633622 | MN797717 | MH300345 | KR823247 | HE590958 | KC888258 | ON411987 | MW955987 |
| MN799821 | MN425195 | MN633782 | MN797556 | MH632702 | KR823245 | JN848892 | KC888383 | ON411964 | MW954830 |
| MN800136 | MN425172 | MN633621 | MN797545 | MH632651 | KR823243 | JN848847 | KC888320 | ON411963 | MW955704 |
| MN799817 | MN425139 | MN633450 | MN797432 | MH632310 | KR823229 | JN848883 | KC888254 | ON411962 | MW955875 |
| MN799816 | MN425135 | MN633752 | MN797425 | KY244825 | KR823206 | JN848840 | KC888354 | ON411994 | MW956656 |
| MN801971 | MN425077 | MN633599 | MN797405 | MG450594 | KR823202 | AY008717 | KC888249 | ON411961 | MW956287 |
| MN801639 | MN425063 | MN462778 | MN797369 | KY713471 | KR823198 | FJ387039 | KC888280 | ON411952 | MW955805 |
| MN800358 | MN425055 | MN462779 | MN797289 | KY713394 | KR823179 | HG421508 | KC888246 | ON411951 | MW954714 |
| MN799814 | MN425038 | MN462728 | MN797259 | MH632352 | KR823171 | JX412342 | KR187197 | ON411986 | MW955596 |
| MN800736 | MN425026 | MN462727 | MN797208 | MG706765 | KR823160 | JQ028422 | KC888343 | ON411949 | MW955533 |
| MN800394 | MN424987 | MN633747 | MN797166 | MG706641 | KR823010 | JX960611 | KC888223 | ON411985 | MW955417 |
| MN799851 | MN425081 | MN633748 | MN797111 | MH632389 | KR822990 | JX112851 | KC888230 | ON411948 | MW955534 |
| MN799840 | MN424956 | MN633592 | MN796922 | MH632330 | KR822989 | JX112858 | KC888220 | ON411984 | MW956892 |
| MN799812 | MN425754 | MN633566 | MN796612 | MH632329 | KR822986 | JX112857 | KC888290 | ON411947 | MW957030 |
| MN799809 | MN424953 | MN633565 | MN797804 | MH300438 | KR822977 | JX112845 | KC888272 | ON411983 | MW955527 |
| MN803050 | MN425000 | MN633562 | MN797705 | MH300414 | KR822972 | JX112842 | KC888218 | ON411946 | MW954905 |
| MN801715 | MN424972 | MN633558 | MN796739 | MH300367 | KR822957 | JX112836 | KC888282 | ON411981 | MW955465 |
| MN799802 | MN424951 | MN633508 | MN796613 | MH300250 | KR824019 | JX112832 | KC888219 | ON411945 | MW954262 |
| MN801966 | MN424942 | MN633461 | MN797615 | MH028372 | KX583295 | JX112828 | KC888216 | ON411980 | MW954242 |
| MN800958 | MN424971 | MN633483 | MN797444 | MH028356 | KU954698 | JX112826 | KR187781 | ON411944 | MW956215 |
| MN799789 | MN424939 | MN462832 | MN633820 | MH010701 | KU954626 | JX112815 | KC888500 | ON411978 | MW955477 |
| MN801891 | MN425007 | MN462831 | MN633899 | MH010697 | KU050606 | JX112811 | KC888488 | ON411943 | MW954718 |
| MN800221 | MN424954 | MN462745 | MN633902 | KY713562 | KU050218 | JQ302753 | KC888356 | ON411976 | MW954118 |
| MN799770 | MN424927 | MN462707 | MN633762 | KY713420 | KT379595 | JQ302744 | KC888340 | ON411939 | MW956248 |
| MN800517 | MN424926 | MN462749 | MN798543 | MH632394 | KT379531 | JQ302708 | KC888241 | ON411975 | MW955429 |
| MN800348 | MN425530 | MN633749 | MN797164 | KY226196 | KT379518 | JQ302705 | KC888398 | ON411938 | MW955337 |
| MN800342 | MN425436 | MN633513 | MN633826 | KY226195 | KT379514 | JQ302698 | KC888314 | ON411935 | MW955142 |
| MN799765 | MN424974 | MN462766 | MN633795 | KY226211 | KT379071 | JQ302692 | KC888211 | ON411934 | MW955240 |
| MN800993 | MN424923 | MN462768 | MN633790 | KY226187 | KR823999 | JQ302682 | KC888390 | ON411972 | MW955231 |
| MN801906 | MN424928 | MN462800 | MN633717 | KY226134 | KR823822 | JQ302679 | KC888341 | ON411933 | MW957401 |
| MN801255 | MN424922 | MN462799 | MN633655 | KY226130 | KR823766 | JQ302675 | KC888209 | ON411971 | MW955009 |
| MN801218 | MN425083 | MN462731 | MN633643 | KY226085 | KR823601 | JQ302674 | KC888214 | ON411932 | MW955176 |
| MN801827 | MN425021 | MN462838 | MN633470 | KY226083 | KR823468 | JQ302672 | KC888202 | ON411969 | MW954920 |
| MN800994 | MN425025 | MN462767 | MK771321 | KY226082 | KR823412 | JQ302668 | KP992356 | ON411928 | MW955161 |
| MN801797 | MN424912 | MK771204 | MK771237 | KY226075 | KR823348 | JQ302646 | KP992354 | ON411966 | MW957330 |
| MN802129 | MN425461 | MN633701 | MN799578 | KY226061 | KR823211 | JQ302597 | KP992357 | ON411927 | MW955108 |
| MN802111 | MN425141 | MN799747 | MN799442 | KY226056 | KR823204 | JQ302574 | KC888414 | ON411965 | MW955159 |
| MN801677 | MN425034 | MN799728 | MN799279 | KY022563 | KR823178 | JQ302550 | KC888330 | ON411926 | MW954531 |
| MN801247 | MN424895 | MN799701 | MN799278 | KY022540 | KR823168 | JQ028351 | KC888325 | ON412002 | MW957600 |
| MN800798 | MN425844 | MN799692 | MN799270 | KY022536 | KR823164 | JQ028324 | KC888300 | ON411960 | MW955810 |
| MN801137 | MN425345 | MN799679 | MN799250 | KX791618 | KR823070 | JQ028261 | KC888419 | ON411959 | MW955107 |
| MT589985 | MN425312 | MN799675 | MN799069 | KX791619 | KT379609 | JQ028240 | KC352177 | ON411925 | MW954473 |
| MT589542 | MN425244 | MN799537 | MN798984 | KX791608 | KT379695 | JQ028227 | KC352174 | ON411871 | MW954667 |
| MT589067 | MN425160 | MN799495 | MN798806 | KX791595 | KU871461 | HE591032 | KC352143 | ON411922 | MW955093 |
| MT589450 | MN425089 | MN799493 | MN798800 | KX791531 | KT379635 | HE591003 | KC888715 | ON411956 | MW954786 |
| MT589428 | MN425069 | MN799490 | MN798667 | KX583284 | KT379620 | HE590979 | KC888673 | ON411917 | MW954210 |
| MT588946 | MN425047 | MN799440 | MN798621 | KX583271 | KR823295 | HE590978 | KC888433 | ON411955 | MW954709 |
| MT589379 | MN425040 | MN799732 | MN798360 | KX306168 | KT379094 | HE590971 | KC352108 | ON411916 | MW955724 |
| MN801297 | MN424931 | MN799407 | MN798181 | KX306164 | KT379088 | HE590961 | KC352155 | ON411950 | MW955675 |
| MN799877 | MN425749 | MN799378 | MN798148 | KX306162 | KR823149 | HE590954 | KC888667 | ON411910 | MW955496 |
| MN799875 | MN424934 | MN799435 | MN798134 | KX306161 | KT379509 | HE590925 | KC352147 | ON411979 | MW955063 |
| MT590087 | MN424933 | MN799370 | MN798090 | KX306138 | KR823858 | GU564229 | KC352084 | ON411905 | MW956610 |
| MT589353 | MN424932 | MN799424 | MN798003 | KX306139 | KR823590 | GU564227 | KR188243 | ON411942 | MW954798 |
| MT589349 | MN424893 | MN799357 | MN797925 | KX306133 | KT379187 | GU564223 | KF857459 | ON411904 | MW956227 |
| MT589326 | MN424890 | MN799350 | MN797834 | KX306124 | KR822961 | GQ845126 | KF857454 | ON411977 | MW954745 |
| MT589649 | MN425581 | MN799356 | MN797554 | KX306158 | KR823353 | FM252030 | KF857443 | ON411903 | MW954642 |
| MT589005 | MN425270 | MN799349 | MN797553 | KX306101 | KR824028 | FM252029 | KF857437 | ON411970 | MW954633 |
| MT589407 | MN424955 | MN799395 | MN797532 | KX306104 | KT379662 | FM252028 | KF857423 | ON411930 | MW956058 |
| MT589237 | MN424884 | MN799332 | MN797437 | KX306094 | KR823864 | FM252026 | KF857413 | ON411929 | MW954277 |
| MT589025 | MN424878 | MN799281 | MN797433 | KX306152 | KT379743 | FM252025 | KF857411 | ON411898 | MW955022 |
| MT589772 | MN425045 | MN799754 | MN797331 | KX306091 | KT379673 | FM252023 | KF857405 | ON411897 | MW955794 |
| MT589661 | MN424877 | MN799635 | MN797261 | KX306105 | KR823536 | FM252022 | KF857395 | ON411957 | MW955753 |
| MT589387 | MN424978 | MN799272 | MN797099 | KX306102 | KR823945 | FM252019 | KF857390 | ON411923 | MW953893 |
| MT590022 | MN424873 | MN799549 | MN797030 | KX306090 | KU954695 | FM252018 | KF857381 | ON411895 | MW953609 |
| MT589028 | MN425318 | MN799252 | MN796995 | KX306111 | KR823565 | FM252017 | KF857367 | ON411867 | MW951743 |
| MT589917 | MN425033 | MN799249 | MN796952 | KX306092 | KR823505 | FM252016 | KF857366 | ON411921 | MW953255 |
| MT590203 | MN424871 | MN799247 | MN796926 | KX306089 | KR823572 | FM252015 | KF857364 | ON411920 | MW953157 |
| MT589395 | MN424935 | MN799222 | MN796879 | KX306108 | KR823500 | FM252014 | KF857362 | ON411894 | MW952997 |
| MT589388 | MN424859 | MN799201 | MN796829 | KX306088 | KU050405 | FM252013 | KF857361 | ON411919 | MW952996 |
| MN802674 | MN425752 | MN799154 | MN796818 | KX306072 | KU050325 | FM252012 | KR188102 | ON411893 | MW953443 |
| MT590283 | MN425154 | MN799153 | MN796751 | KX306170 | KT378937 | FM252011 | KR187823 | ON411892 | MW952880 |
| MT589378 | MN424857 | MN799414 | MN799235 | KX306078 | KR823916 | FM252010 | KR187534 | ON411891 | MW952094 |
| MT589299 | MN425784 | MN799146 | MN798408 | KX306070 | KR823674 | FM252009 | KR187944 | ON411866 | MW957041 |
| MT590134 | MN425747 | MN799133 | MN797967 | KX306099 | KR823431 | FM252008 | KC888378 | ON411890 | MW955802 |
| MT590190 | MN424840 | MN799449 | MN797233 | KX306065 | KR823422 | FM252007 | KC888284 | ON411954 | MW957422 |
| MT589951 | MN425688 | MN799135 | MN797173 | KX306068 | KU954749 | FM252004 | KF914622 | ON411915 | MW954960 |
| MT590085 | MN424919 | MN799079 | MN796965 | KX306028 | KR823088 | FM252003 | KR106953 | ON411914 | MW956226 |
| MT590182 | MN424854 | MN799056 | MN633751 | KX306077 | KR823266 | FM252002 | KR106947 | ON411888 | MW957298 |
| MT590239 | MN424834 | MN799036 | MN799381 | KX306027 | KT893578 | FM252001 | KR106934 | ON411912 | MW957512 |
| MT590266 | MN424915 | MN799420 | MN799353 | KX306175 | KR823311 | FM252000 | KR106930 | ON411886 | MW957112 |
| MT590048 | MN424831 | MN799034 | MN799331 | KX306174 | KR823218 | FM251999 | KR106928 | ON411911 | MW956829 |
| MT589708 | MN424830 | MN799033 | MN799277 | KX306163 | KR823018 | FM251998 | KR106924 | ON411885 | MW956955 |
| MT589692 | MN425756 | MN799286 | MN799258 | KX306100 | KT379538 | FM251997 | KR106908 | ON411870 | MW954944 |
| MT589807 | MN425751 | MN799018 | MN799159 | KX306083 | KR823907 | FM251996 | KR106900 | ON411868 | MW952516 |
| MT589935 | MN424894 | MN799391 | MN798972 | KX306069 | KX791609 | FM251995 | KR106897 | ON411869 | MW954687 |
| MT590178 | MN424829 | MN799000 | MN798936 | KX306026 | KR823288 | FM251994 | KR106889 | ON411858 | MW956922 |
| MT589863 | MN424819 | MN799467 | MN798837 | KX306157 | KR823967 | FM251993 | KR106887 | ON411857 | MW957105 |
| MT589501 | MN425703 | MN798982 | MN798772 | KX306135 | KY226059 | FM251992 | KR106886 | OP277848 | MW955782 |
| MT589500 | MN424818 | MN798939 | MN798765 | KX306114 | KT379631 | FM251991 | KR106884 | OP277847 | MW956808 |
| MT589514 | MN424816 | MN799437 | MN798235 | KX306071 | KT379592 | FM251990 | KR106874 | ON411896 | MW954785 |
| MT589491 | MN424809 | MN798935 | MN798027 | KX306063 | KT379582 | FM251988 | KR106870 | ON411958 | MW954680 |
| MT589159 | MN424793 | MN799340 | MN797964 | KX306018 | KT379546 | FM251987 | KR106869 | ON411864 | MW956668 |
| MT589160 | MN424812 | MN798889 | MN797935 | KX306013 | KT379522 | FM251985 | KR106866 | ON411863 | MW955939 |
| MT589196 | MN425153 | MN799193 | MN797903 | KX306012 | KT379521 | FM251983 | KR106857 | OR606504 | MW956392 |
| MT590228 | MN425670 | MN799723 | MN797897 | KX306107 | KT379413 | FM251981 | KR106853 | OR606478 | MW956377 |
| MT590157 | MN424824 | MN798849 | MN797558 | KX306004 | KT379247 | FM251980 | KR106848 | OP831061 | MW954864 |
| MT590078 | MN424814 | MN798843 | MN797487 | KX306036 | KT379185 | FM251979 | KR106843 | OR606508 | MW956176 |
| MT589997 | MN424790 | MN799111 | MN797479 | KX306002 | KT379111 | FM251978 | KR106842 | OR606461 | MW957075 |
| MT589534 | MN424789 | MN798793 | MN797435 | KX306085 | KT378930 | FM251977 | KR106828 | OR606480 | MW955165 |
| MT589499 | MN424781 | MN462770 | MN797311 | KX305997 | KT378725 | FM251964 | KR106822 | OR606473 | MW956982 |
| MT589823 | MN424780 | MN799445 | MN797297 | KX306067 | KR823867 | FM251962 | KR106809 | OR606459 | MW956065 |
| MT589780 | MN424778 | MN798728 | MN797269 | KX305995 | KR823825 | FM251960 | KR106808 | OP831056 | MW954614 |
| MT589591 | MN424776 | MN799464 | MN797263 | KX306021 | KR823272 | FM251959 | KR106986 | OP830947 | MW956048 |
| MT589934 | MN424775 | MN798857 | MN797191 | KX305993 | KR823259 | FM251956 | KR106982 | ON411941 | MW955592 |
| MT589998 | MN424702 | MN798818 | MN797143 | KX305991 | KT379718 | FM251952 | KR106973 | ON411940 | MW954240 |
| MT589505 | MN424701 | MN798715 | MN797009 | KX305990 | KT379566 | JQ028399 | KR106971 | ON411902 | MW955902 |
| MN800893 | MN424704 | MN798686 | MN796978 | KX305987 | KT379167 | GQ290710 | KP992399 | ON411973 | MW955779 |
| MT986174 | MN424699 | MN798760 | MN796692 | KX305984 | KX791534 | JN848919 | KR106873 | ON411900 | MW956056 |
| MN802807 | MN424700 | MN798647 | MN797894 | KX306055 | KX791517 | JN848917 | KC352096 | ON411931 | MW955839 |
| MN801817 | MN424696 | MN798642 | MN797608 | KX305977 | KT379819 | JN848916 | KC352079 | ON411899 | MW957502 |
| MN801368 | MN424705 | MN799752 | MN797241 | KY022560 | KT379545 | JN848915 | KC352076 | ON411918 | MW955833 |
| MN801267 | MN424694 | MN799649 | MN633733 | KX159285 | KT379261 | JN848907 | KR188405 | ON411889 | MW954712 |
| MN801158 | MN424693 | MN798578 | MN633773 | KY226111 | KR823097 | JN848899 | KR188152 | ON411953 | MW955692 |
| MN801261 | MN424692 | MN798593 | MN633474 | KU954717 | KR823976 | JN848896 | KR188036 | ON411913 | MW954644 |
| MN800250 | MN424669 | MN798572 | MN633473 | KU954703 | KX791603 | JN848894 | KR188014 | ON411887 | MW955456 |
| MN801852 | MN424648 | MN798690 | MN633466 | KU954748 | KX583334 | JN848888 | KR187980 | ON411909 | MW955253 |
| MT589007 | MN424685 | MN798556 | MN798811 | KU954637 | KX583306 | JN848887 | KR106836 | ON411884 | MW956423 |
| MN802688 | MN424647 | MN799214 | MN798609 | KU954747 | KX583270 | JN848875 | KR188079 | ON411860 | MW956322 |
| MT986074 | MN424630 | MN798554 | MN798026 | KU954636 | KX306171 | JN848874 | KJ401764 | ON411859 | MW956044 |
| MN802379 | MN424631 | MN798552 | MN797797 | KU871424 | KX306066 | JN848871 | KC888389 | ON411883 | MW955454 |
| MT589868 | MN424627 | MN798567 | MN633690 | KU871418 | KX306022 | JN848869 | KF857441 | ON411879 | MW955043 |
| MT589839 | MN424731 | MN798538 | MN633913 | KU378041 | KU954572 | JN848865 | KF857410 | ON411878 | MW955427 |
| MT589532 | MN424618 | MN798876 | MN633912 | KU378040 | KU050525 | JN848859 | KR188384 | ON411882 | MW955164 |
| MN802921 | MN424730 | MN798521 | MN797684 | KU378038 | KU050453 | JN848851 | KR188259 | ON411877 | MW955419 |
| MN800669 | MN424604 | MN798491 | MN799646 | KU364413 | KU050396 | JN848846 | KR188245 | ON411876 | MW954972 |
| MT589545 | MN424725 | MN798477 | MN799478 | KU364412 | KT379913 | JN848845 | KR188239 | ON411937 | MW955332 |
| MT589427 | MN424602 | MN798476 | MN797968 | KX505984 | KT379870 | JN848843 | KR188238 | ON411875 | MW955099 |
| MT588997 | MN424601 | MN799656 | MN798399 | KX505982 | KT379809 | HG421509 | KR188057 | ON411901 | MW954889 |
| MN800814 | MN424588 | MN798475 | MN799156 | KX505977 | KT379750 | JX112801 | KR188005 | ON411874 | MW955098 |
| MN802989 | MN424726 | MN798580 | MN798781 | KU050658 | KT379331 | JQ901082 | KR187949 | ON411856 | MW955440 |
| MN802588 | MN424587 | MN798461 | MN798437 | KU050657 | KT379140 | JQ901078 | KR187908 | ON411865 | MW955348 |
| MN801557 | MK287294 | MN798460 | MN798291 | KU050648 | KT379106 | JQ901064 | KJ401682 | MZ956108 | MW954553 |
| MN800118 | MK287214 | MN798459 | MN798225 | KU050647 | KT379000 | JQ901054 | KR187983 | MZ956102 | MW954430 |
| MN802086 | MK287209 | MN798464 | MN798190 | KU050578 | KT378953 | JQ901050 | KJ401727 | MZ956083 | MW954428 |
| MT589099 | MK287228 | MN798433 | MN798060 | KU050564 | KT378853 | JQ901035 | KJ401664 | MZ956079 | MW954125 |
| MT590122 | MK287180 | MN798422 | MN797914 | KU050560 | KT378775 | JQ302662 | KC203320 | MZ956069 | MW955012 |
| MN801388 | MK287191 | MN798420 | MN797825 | KU050557 | KT378773 | JQ028206 | KJ401753 | MZ956067 | MW954124 |
| MN801296 | MK287174 | MN798412 | MN797572 | KU050556 | KT378718 | HQ215580 | KJ401746 | MZ956092 | MW953930 |
| MN800823 | MK287171 | MN799369 | MN797518 | KY226206 | KT192019 | HE590996 | KC203319 | MZ956066 | MW954852 |
| MN801766 | MK287167 | MN798410 | MN797454 | KU050544 | KR823997 | HE590900 | KJ401747 | MZ956072 | MW954734 |
| MN801722 | MK287179 | MN799059 | MN797236 | KU050510 | KR823409 | GQ290723 | KJ401728 | MZ956026 | MW955815 |
| MT590222 | MK287157 | MN798406 | MN797017 | KU050508 | KR823400 | GQ290722 | KR188252 | MZ956019 | MW953844 |
| MT161986 | MK287193 | MN798558 | MN796852 | KU050561 | KR823321 | JN848908 | KR188244 | MZ956037 | MW953096 |
| MT589038 | MK287131 | MN798381 | MN796597 | KU050506 | KR823236 | JN848900 | KR187991 | MZ956004 | MW953776 |
| MN800238 | MK287114 | MN798910 | MN797134 | KU050505 | KR823090 | JN848882 | KR187907 | OP190414 | MW953010 |
| MT589978 | MK287094 | MN798367 | MN797767 | KU050504 | KU871409 | JN848863 | KR187871 | OR522338 | MW953509 |
| MT588966 | MK287183 | MN799463 | MN799313 | KU050502 | KT378808 | JN848862 | KR187857 | OR521456 | MW953161 |
| MT588945 | MK287086 | MN798366 | MN798481 | KU050481 | KR823586 | JQ302652 | KR187851 | OR522242 | MW953751 |
| MT589794 | MN427107 | MN798351 | MN798590 | KU050479 | KR823527 | JX960602 | KR187793 | OR522192 | MW952044 |
| MT589583 | MK892952 | MN798714 | MN799568 | KU050667 | KT379135 | JQ028316 | KR187777 | OP190504 | MW953374 |
| MT589254 | MK892950 | MN798340 | MN799387 | KU050475 | KX583302 | HG421631 | KR187697 | OL684389 | MW952124 |
| MT589347 | MN427363 | MN798723 | MN799290 | KU050579 | KR823944 | JQ901095 | KR187690 | OR522054 | MW953290 |
| MN802987 | MN427242 | MN798320 | MN798920 | KU050533 | KT379099 | JQ901023 | KR187649 | OR522052 | MW952671 |
| MN802946 | MN427096 | MN798314 | MN633516 | KU050418 | KU050247 | JQ028382 | KR187632 | OR522435 | MW953158 |
| MN802923 | MK892927 | MN798313 | MK771241 | KU050672 | KR823935 | JQ901075 | KR187590 | OR521981 | MW953071 |
| MN802783 | MN427147 | MN798812 | MN633497 | KU050401 | KR823909 | KC183780 | KR187564 | OR521382 | MW952723 |
| MN802513 | MK892926 | MN798394 | MN797687 | KU050499 | KR823633 | JX412332 | KR187537 | OP831140 | MW952820 |
| MN802442 | MN427217 | MN798305 | MN797629 | KU050392 | KR823357 | JQ235020 | KR187449 | OP831046 | MW954892 |
| MN802432 | MN427216 | MN799224 | MN797617 | KU050370 | KT379378 | JX392381 | KR187404 | OP831012 | MW952806 |
| MN802158 | MK892925 | MN798268 | MN797599 | KU050295 | KR824020 | JQ302558 | KR187386 | OP830916 | MW952704 |
| MN802141 | MN427292 | MN799044 | MN797293 | KU050674 | KY226166 | HQ215552 | KR187366 | OP830912 | MW955697 |
| MN801938 | MN427247 | MN798893 | MN799717 | KU050271 | KU954742 | HG421696 | KR187330 | OP830911 | MW956340 |
| MN801920 | MK892924 | MN798238 | MN799067 | KU050269 | KU954658 | HG421614 | KR187318 | OK315199 | MW957037 |
| MN801628 | MK892923 | MN799012 | MN798478 | KU050266 | KU954643 | HG421539 | KR187311 | OK315198 | MW956173 |
| MN801610 | MN427208 | MN798451 | MN797581 | KY226159 | KU954604 | HG421522 | KR187298 | OK315183 | MW951691 |
| MN801597 | MK892919 | MN798214 | MN797370 | KU050265 | KU954603 | HG421462 | KR187244 | OK315135 | MW955347 |
| MN801566 | MN427064 | MN798215 | MN797180 | KY226168 | KU954595 | HE590926 | KR187238 | OK315133 | MW955313 |
| MN801453 | MK892902 | MN798195 | MN796642 | KU050250 | KU954579 | AF503396 | KR187224 | OK315126 | MW954586 |
| MN801452 | MN427154 | MN798194 | MN633903 | KX505970 | KU954574 | AF286230 | KR187215 | OK315029 | MW953790 |
| MN801361 | MK892900 | MN798994 | MN633707 | KU050244 | KT379623 | AF286226 | KR106839 | OK315001 | MW954726 |
| MN801227 | MN427312 | MN798191 | MK771291 | KX505972 | KT379543 | FJ387090 | KP992426 | OP830985 | MW953550 |
| MN801108 | MN427263 | MN799731 | MK771288 | KU050241 | KT379494 | JN848955 | KP992407 | MZ269681 | MW956177 |
| MN801092 | MN427367 | MN798694 | MK771185 | KU050227 | KT379493 | JN848951 | KC888723 | OR521435 | MW955531 |
| MN800963 | MN427143 | MN798213 | MK771158 | KU050224 | KT379152 | JX960600 | KC888692 | OR522412 | MW955439 |
| MN800924 | MN427253 | MN798187 | MN799761 | KU050222 | KT379045 | JQ302604 | KC888664 | OR521378 | MW956152 |
| MN800872 | MN427130 | MN798713 | MN799755 | KU050221 | KT378712 | JQ235012 | KC888648 | MW957881 | MW956150 |
| MN800821 | MN427105 | MN798186 | MN799671 | KU050213 | KR824031 | HQ215583 | KC888601 | OR521708 | MW955455 |
| MN800764 | MN427071 | MN798180 | MN799616 | KU050284 | KR823813 | HG421630 | KC888592 | OP190665 | MW955413 |
| MN800556 | MN427079 | MN798179 | MN799598 | KU050212 | KR823724 | HE590964 | KC888570 | OP190645 | MW954031 |
| MN800534 | MN427164 | MN798174 | MN799559 | KU050283 | KR823691 | JN848949 | KC888566 | OP190555 | MW953879 |
| MN800455 | MN427123 | MN798173 | MN799479 | KU050211 | KR823532 | JQ901069 | KC888537 | OR521432 | MW956087 |
| MN800400 | MN427108 | MN799482 | MN799345 | KU050282 | KR823529 | JQ901072 | KC888515 | OR521754 | MW956619 |
| MN800363 | MN427081 | MN799021 | MN799339 | KU050226 | KR823528 | JQ901027 | KC888464 | OR522273 | MW955670 |
| MN800314 | MK892879 | MN798172 | MN799106 | KU050208 | KR823382 | JQ901026 | KC888443 | OR522270 | MW952424 |
| MN800309 | MK892878 | MN798164 | MN798996 | KU050281 | KR823381 | JQ235021 | KC888269 | OR521476 | MW954021 |
| MN800268 | MK892874 | MN798328 | MN798917 | KU050207 | KU050474 | HG421562 | KC352223 | OR521454 | MW954463 |
| MN800169 | MK892928 | MN798159 | MN798862 | KY226150 | KU050267 | JN848935 | KJ401668 | OR522134 | MW954386 |
| MN800151 | MK892933 | MN799491 | MN798799 | KU050276 | KT379303 | JQ901094 | KR188438 | OR522129 | MW954338 |
| MN800063 | MK892888 | MN798141 | MN798782 | KU050202 | KT379184 | JQ901076 | KR188404 | OR522039 | MW954307 |
| MN800056 | MN426699 | MN798140 | MN798754 | KU050274 | KT378842 | JQ901073 | KR188317 | OP191187 | MW954020 |
| MN800029 | MN426427 | MN798139 | MN798719 | KU050201 | KT378666 | JN848934 | KR188105 | OR521715 | MW953940 |
| MN799991 | MN426396 | MN462772 | MN798676 | KU050272 | KR824012 | JQ235014 | KR188038 | OR521956 | MW954941 |
| MN799952 | MN425937 | MN798127 | MN798668 | KU050200 | KR823957 | HE590974 | KR187959 | OP831006 | MW954764 |
| MN799922 | MK409843 | MN798126 | MN798633 | KT893677 | KR823942 | KF714419 | KR187865 | OP830984 | MW954216 |
| MN799916 | MK409841 | MN798382 | MN798627 | KT893669 | KR823619 | KR822926 | KR187844 | OR606506 | MW953887 |
| MN799844 | MK409828 | MN798100 | MN798570 | KT893633 | KR823616 | KJ570822 | KR187718 | OR606503 | MW953845 |
| MN799833 | MK409827 | MN798792 | MN798479 | KT893617 | KR823372 | KJ570788 | KR187626 | OR606502 | MW952957 |
| MN799769 | MN426186 | MN798080 | MN798453 | KT893606 | KR823977 | KF927151 | KC888642 | OR606501 | MW954729 |
| MN799768 | MK409816 | MN798327 | MN798452 | KT893600 | KR823934 | KJ570824 | KC352192 | OR606500 | MW953935 |
| MN801823 | MK409811 | MN798078 | MN798353 | KU871467 | KR823918 | KF835184 | KC888392 | OR606498 | MW955285 |
| MT986072 | MN427190 | MN798717 | MN798290 | KT893567 | KR823914 | KC924493 | KP992402 | OR606496 | MW955185 |
| MT590143 | MK892875 | MN798061 | MN798239 | KT893524 | KR823912 | KP178427 | KP992351 | OR606495 | MW954597 |
| MT588973 | MK892943 | MN798147 | MN798226 | KT893520 | KR823911 | KP178420 | KR187625 | OR606494 | MW954587 |
| MT590225 | MN427115 | MN799545 | MN798149 | KT893498 | KR823079 | KJ570806 | KJ401689 | OR606492 | MW954440 |
| MT590199 | MK892891 | MN798189 | MN798063 | KT893496 | KY226153 | KJ570805 | KR188450 | OR606491 | MW954854 |
| MT590201 | MK892908 | MN798037 | MN797997 | KT893494 | KR823972 | KJ570804 | KR188444 | OR606490 | MW954757 |
| MT589947 | MK892890 | MN799087 | MN797980 | KT893492 | KR823947 | KM258858 | KR188434 | OR606489 | MW954572 |
| MT589373 | MK892914 | MN798032 | MN797942 | KU161144 | KX791610 | KF835247 | KR188427 | OR606488 | MW954477 |
| MT161687 | MK892884 | MN798863 | MN797927 | KT625869 | KU954646 | KF835208 | KR188412 | OR606487 | MW954251 |
| MT589177 | MN427335 | MN798030 | MN797896 | KU161145 | KT379232 | KF835191 | KR188370 | OR606486 | MW954619 |
| MT590050 | MN427327 | MN798308 | MN797880 | KT625865 | KT379645 | KF835147 | KR188335 | OR606482 | MW954758 |
| MT589442 | MN427314 | MN798021 | MN797816 | KT625855 | KX583335 | KF835140 | KR188332 | OR606481 | MW954432 |
| MN802966 | MN427214 | MN799401 | MN797784 | KT625854 | KX583265 | KF835202 | KR188271 | OR606475 | MW953917 |
| MN802911 | MN427125 | MN799302 | MN797711 | KT625806 | KU954714 | KF835126 | KR188233 | OR606474 | MW954894 |
| MN802873 | MN427072 | MN798474 | MN797650 | KT625805 | KU954710 | KF835118 | KR188199 | OR606472 | MW952972 |
| MN802791 | MN427068 | MN798016 | MN797600 | KT379928 | KU954651 | KJ614119 | KR188197 | OR606471 | MW956400 |
| MN802527 | MN427063 | MN798094 | MN797573 | KT379926 | KU954618 | KJ614083 | KR188168 | OR606470 | MW955726 |
| MN802486 | MN427062 | MN798961 | MN797568 | KU050324 | KU954573 | KJ614082 | KR188136 | OR606469 | MW955752 |
| MN802326 | MN336579 | MN798832 | MN797526 | KT379782 | KU050596 | KJ614081 | KR188091 | OR606467 | MW955254 |
| MN802186 | MN336533 | MN798002 | MN797515 | KT379787 | KU050590 | KJ614076 | KR188069 | OR606466 | MW956267 |
| MN802114 | MN601616 | MN799686 | MN797476 | KT379826 | KU050562 | KJ614071 | KR188064 | OR606463 | MW952452 |
| MN802083 | MN601615 | MN797998 | MN797413 | KT379768 | KU050355 | KJ614063 | KR188004 | OR606462 | MW952825 |
| MN801985 | MN601610 | MN798856 | MN797317 | KT379763 | KU050313 | KJ614059 | KR187994 | OR606460 | MW953560 |
| MN801893 | MN601609 | MN798956 | MN797300 | KT379747 | KU050306 | KJ614057 | KR187967 | OR522450 | MW953458 |
| MN801729 | MN601607 | MN798040 | MN797238 | KT379780 | KT379842 | KJ614050 | KR187945 | OR521716 | MW953697 |
| MN801661 | MN601581 | MN798736 | MN797217 | KT379683 | KT379761 | KJ614048 | KR187935 | OR522174 | MW953234 |
| MN801604 | MN601580 | MN798566 | MN797210 | KU364392 | KT379740 | KJ614047 | KR187902 | OR522120 | MW954318 |
| MN801342 | MN601563 | MN797976 | MN797201 | KT379689 | KT379658 | KJ614046 | KR187899 | MZ956070 | MW952763 |
| MN801331 | MN601562 | MN798716 | MN797162 | KT379663 | KT379648 | KJ614031 | KR187796 | MW957786 | MW951900 |
| MN801320 | MN601561 | MN798161 | MN797131 | KT379619 | KT379626 | KJ614030 | KR187790 | OR521666 | MW955353 |
| MN801318 | MN601536 | MN797970 | MN797130 | KT379614 | KT379504 | KJ614025 | KR187731 | OR521408 | MW952441 |
| MN801130 | MN601535 | MN799742 | MN797120 | KU954607 | KT379268 | KJ614202 | KR187709 | OR522111 | MW953026 |
| MN801075 | MN424909 | MN799360 | MN797096 | KT379590 | KT379101 | KJ614196 | KR187633 | OR521423 | MW953648 |
| MN801026 | MN601519 | MN797966 | MN797094 | KT379633 | KT379091 | KJ614186 | KR187617 | OP191111 | MW952553 |
| MN801011 | MN601518 | MN798649 | MN797082 | KT379580 | KT379053 | KJ614154 | KR187613 | OP329541 | MW952598 |
| MN800969 | MN427171 | MN799558 | MN797056 | KX378999 | KT378834 | KF835187 | KR187577 | OP329475 | MW953117 |
| MN800724 | MK892973 | MN798591 | MN797010 | KT379711 | KT378706 | KC924463 | KR187574 | OR521400 | MW952623 |
| MN800722 | MK892899 | MN798710 | MN796996 | KT379572 | KR824030 | KM395732 | KR187567 | OP191631 | MW952474 |
| MN800663 | MN427259 | MN798456 | MN796990 | KT379556 | KR824014 | KJ401624 | KR187409 | OP191024 | MW953000 |
| MN800562 | MN427087 | MN798941 | MN796983 | KT379835 | KR824000 | KP418612 | KR187406 | MW957854 | MW952991 |
| MN800411 | MK892922 | MN798884 | MN796957 | KT379843 | KR823905 | KJ401558 | KR187319 | OP831051 | MW952986 |
| MN800277 | MK892876 | MN798106 | MN796912 | KT379508 | KR823841 | KP250733 | KR187296 | OP191201 | MW952700 |
| MN800215 | MN336586 | MN797959 | MN796846 | KT379496 | KR823771 | KM258794 | KC888710 | OP191041 | MW953838 |
| MN800127 | MN336583 | MN799023 | MN796777 | KT379495 | KR823716 | KM258841 | KC888696 | OP190867 | MW955014 |
| MN800069 | MN427015 | MN797951 | MN796618 | KT379477 | KR823715 | KJ570799 | KC888653 | OK315203 | MW951895 |
| MN799796 | MK892866 | MN799751 | MN796610 | KT379469 | KR823714 | KJ570797 | KC888639 | OP329424 | MW951806 |
| MT589330 | MN427368 | MN799762 | MN796608 | KT379796 | KR823705 | KP418621 | KC888638 | MW957933 | MW951792 |
| MT589653 | MN427032 | MN797921 | MN796601 | KT379526 | KR823667 | KJ401464 | KC888636 | OP831118 | MW957238 |
| MT589519 | MK892873 | MN798207 | MN799268 | KT379523 | KR823595 | KP418626 | KC888504 | OP831052 | MW954862 |
| MN801589 | MK892910 | MN797904 | MN798855 | KT379451 | KR823521 | KP418622 | KC888472 | OR522172 | MW957086 |
| MN802183 | MK892867 | MN799217 | MN797378 | KT379666 | KR823512 | KP418611 | KC888431 | OR521901 | MW954888 |
| MN802150 | MK287268 | MN798967 | MN797373 | KT379407 | KR823492 | KM258728 | KC888376 | OR521880 | MW952901 |
| MN801291 | MK287324 | MN797885 | MN796999 | KT379568 | KR823467 | KJ570836 | KC888306 | OR521765 | MW953674 |
| MN800076 | MN427244 | MN799416 | MN796856 | KT379363 | KR823410 | KJ570794 | KC888621 | OP190799 | MW954613 |
| MT589495 | MN427230 | MN797882 | MN796598 | KT379357 | KR823375 | KP250786 | KC352141 | OP190577 | MW954521 |
| MT589284 | MN427061 | MN798729 | MN799184 | KT379770 | KR823371 | KP250684 | KC352124 | OP190494 | MW954129 |
| MT589106 | MK543684 | MN798357 | MN797294 | KT379285 | KR823359 | KJ401532 | KC352114 | OP190443 | MW952699 |
| MT589859 | MK543674 | MN798331 | MN633545 | KU050289 | KR823358 | KJ401488 | KC352111 | OP190428 | MW954559 |
| MT590164 | MK543644 | MN797913 | MN633739 | KT379269 | KR823345 | KP250777 | KC352075 | OP190416 | MW954824 |
| MT589098 | MK543643 | MN797876 | MN798470 | KT379736 | KR823344 | KP250755 | KR188360 | OP190624 | MW954638 |
| MN801262 | MK543579 | MN799633 | MN798925 | KT379309 | KR823337 | KJ401612 | KR188389 | OP190604 | MW954591 |
| MT986805 | MK543578 | MN799384 | MN798162 | KT379260 | KR823331 | KM258717 | KR187862 | MZ956097 | MW954549 |
| MT589960 | MK459298 | MN797875 | MN798007 | KT379244 | KR823327 | KM258682 | KR187663 | OR522421 | MW954256 |
| MT161779 | MK459284 | MN797907 | MN796784 | KT379588 | KR823307 | KJ614120 | KC888476 | OP191603 | MW954151 |
| MN802707 | MN178646 | MN797871 | MN633698 | KT379577 | KR823305 | KJ614069 | KR187800 | OR522037 | MW953871 |
| MN801442 | MK459269 | MN799051 | MN633860 | KT379233 | KR823286 | KJ614036 | KC888677 | OR521978 | MW954449 |
| MT161964 | MN427166 | MN798900 | MN633692 | KT379338 | KR823274 | KJ401485 | KR188182 | OR521654 | MW954228 |
| MW270352 | MK459266 | MN798107 | MN633625 | KT379226 | KR823273 | KJ401478 | KR188072 | OR522357 | MW955418 |
| MN801666 | MN178645 | MN797869 | MN633522 | KT379291 | KR823260 | KR822904 | KC888244 | OR521940 | MW955355 |
| MT589088 | MK459243 | MN798894 | MK459241 | KT379224 | KR823258 | KP250757 | KC352204 | OR521818 | MW954769 |
| MT986099 | MK459290 | MN797862 | MN797639 | KT379552 | KR823249 | KP250711 | KR188067 | OR521793 | MW957631 |
| MN800310 | MK459242 | MN797930 | MN798907 | KT379223 | KR823232 | KM258792 | KR188203 | OP191082 | MW957064 |
| MT986568 | MK459267 | MN797821 | MN799474 | KX791590 | KR823213 | KJ401467 | KJ401608 | MW957654 | MW956077 |
| MN802599 | MK459282 | MN797934 | MN797918 | KT379197 | KR823212 | KJ401445 | KR188310 | OR522228 | MW951924 |
| MT986106 | MK459199 | MN797811 | MN797499 | KT379218 | KR823180 | KM258743 | KR188250 | OR521472 | MW951876 |
| MT986071 | MK459209 | MN798851 | MN797431 | KT379196 | KT379849 | KR822863 | KR188381 | OP191485 | MW951714 |
| MT589730 | MK459193 | MN799358 | MN796796 | KT379195 | KT379847 | KR822858 | KR187547 | OP831116 | MW956455 |
| MT589227 | MK459187 | MN797800 | MN796749 | KT379207 | KT379752 | KJ614156 | KR187424 | OR521900 | MW955742 |
| MT589001 | MK459201 | MN797802 | MN633478 | KT379193 | KT379749 | KR822870 | KP992439 | OP190528 | MW956094 |
| MT588951 | MK459185 | MN797776 | MN797559 | KT379175 | KT379651 | KM258768 | KP992404 | OP190671 | MW953736 |
| MT588943 | MK459114 | MN798530 | MN799489 | KT379212 | KT379647 | KJ401463 | KC888215 | OP191615 | MW956435 |
| MT590205 | MK459139 | MN797857 | MN797809 | KT379169 | KT379629 | KM258715 | KF857401 | OP190510 | MW955466 |
| MT590204 | MK459105 | MN797747 | MN797682 | KT379482 | KT379608 | KP418586 | KF857396 | OR521667 | MW956593 |
| MT590165 | MK459270 | MN799507 | MN797060 | KT379139 | KT379498 | KM258865 | KF857391 | OR522303 | MW955396 |
| MT589322 | MK459100 | MN797743 | MN633586 | KT379302 | KT379434 | KJ401452 | KF857380 | OR522302 | MW953791 |
| MT589949 | MK459130 | MN799261 | MH921141 | KT379259 | KT379381 | KF267702 | KF857373 | MW957729 | MW957494 |
| MT589929 | MK459092 | MN797735 | MH921120 | KT379143 | KT379346 | KJ401424 | KR188054 | MW957901 | MW957258 |
| MT589785 | MK459085 | MN797729 | MH921137 | KT379129 | KT379332 | KJ570845 | KJ401717 | MW957696 | MW955153 |
| MT589605 | MK459083 | MN798376 | MH921117 | KT379296 | KT379284 | KM258802 | KJ401656 | OR521413 | MW957479 |
| MT589403 | MK459159 | MN797710 | MH921097 | KT379110 | KT379231 | KM258787 | KR187985 | MW957857 | MW955993 |
| MT589367 | MK459069 | MN799058 | MH921091 | KT379109 | KT379208 | KJ401542 | KR187741 | OR522429 | MW952438 |
| MT589356 | MK459148 | MN798960 | MH921090 | KT379076 | KT379191 | KM258697 | KR187628 | OR522252 | MW956246 |
| MT590008 | MK459143 | MN797693 | MH921081 | KT379265 | KT379176 | KP250749 | KR187422 | OR521798 | MW956069 |
| MT161925 | MK459054 | MN798584 | MH789906 | KT379068 | KT379160 | KP250720 | KR187368 | OP190689 | MW953474 |
| MT589890 | MK459152 | MN799130 | MH789862 | KT379200 | KT379157 | KM258797 | KR187285 | OP190458 | MW952639 |
| MT589912 | MK459136 | MN797668 | MH789814 | KT379066 | KT379133 | KJ401484 | KR187278 | MW957919 | MW954218 |
| MN803087 | MK459050 | MN799734 | MH789813 | KT379321 | KT379118 | KR822917 | KR187194 | MZ956051 | MW953409 |
| MN803079 | MK459125 | MN797655 | MH789812 | KT379800 | KT379117 | KR822906 | KP992433 | OP191144 | MW953098 |
| MN803077 | MK459046 | MN799083 | MH789855 | KT378987 | KT379100 | KR822887 | KJ401600 | OP190413 | MW953817 |
| MN803070 | MK459297 | MN797644 | MH789769 | KT378977 | KT379070 | KR822868 | KR188190 | OR522396 | MW953927 |
| MN803026 | MK459145 | MN798053 | MH789821 | KT379192 | KT378898 | KR822861 | KR187878 | OR522315 | MW954691 |
| MN802817 | MK459084 | MN798659 | MH789748 | KT378952 | KT378744 | KR822840 | KR187640 | OR522237 | MW954924 |
| MN802755 | MK459096 | MN798341 | MH789926 | KT378933 | KT378732 | KM258734 | KF857382 | OR522220 | MW952404 |
| MN802653 | MK459039 | MN797960 | MH789745 | KU050287 | KR824022 | KJ401495 | KF857379 | OR522163 | MW952417 |
| MN802626 | MK459093 | MN797920 | MH632713 | KT379060 | KR823881 | KJ401553 | KR187468 | OR522161 | MW957036 |
| MN802554 | MK459037 | MN797637 | MH632701 | KU050323 | KX791629 | KM258784 | KP992413 | OR521983 | MW951736 |
| MN802500 | MK459036 | MN798073 | MH632685 | KT378923 | KU954750 | KP250667 | KP992412 | OR521800 | MW955914 |
| MN802464 | MK459121 | MN799057 | MH632684 | KU050319 | KU954739 | KJ614185 | KP992411 | OP190794 | MW955504 |
| MN802449 | MK459116 | MN798200 | MH632681 | KT378916 | KU050550 | KP250791 | KR106891 | OR522160 | MW956864 |
| MN802335 | MK459110 | MN797607 | MH632678 | KT378905 | KT379729 | KJ401439 | KR106881 | OR521724 | MW957607 |
| MN802234 | MK459032 | MN799664 | MH632710 | KU050292 | KT379557 | KP418589 | KR106858 | OR522165 | MW955916 |
| MN802223 | MK459288 | MN797603 | MH632660 | KT379657 | KT379551 | KP418609 | KR106850 | MW957718 | MW955876 |
| MN802209 | MK459031 | MN798995 | MH921177 | KT379047 | KT379290 | KP418617 | KR106833 | MW957878 | MW953401 |
| MN802169 | MN425565 | MN797594 | MH632527 | KT378901 | KT379062 | KP418616 | KR187939 | OR521369 | MW952830 |
| MN802033 | MK459024 | MN797627 | MH632650 | KT379061 | KT378875 | KP418587 | KR187760 | OR522442 | MW952396 |
| MN801993 | MK459238 | MN798946 | MH632552 | KT379007 | KR823629 | KJ401442 | KR187344 | OR522073 | MW952212 |
| MN801842 | MK459051 | MN798298 | MH632512 | KT378900 | KR823507 | KM258761 | KC888721 | OR521859 | MW954273 |
| MN801770 | MK459011 | MN798031 | MH632342 | KT379149 | KR823470 | KR822852 | KR188333 | OR521817 | MW953942 |
| MN801734 | MN178644 | MN797844 | MH632331 | KU050315 | KR823376 | KJ570844 | KJ401737 | OP830923 | MW953551 |
| MN801614 | MK459005 | MN797578 | MH632655 | KT378885 | KR823338 | KP250788 | KJ614035 | MW957678 | MW955700 |
| MN801606 | MK459104 | MN798533 | MH632324 | KT378876 | KR823248 | KM405336 | KJ401437 | OP191540 | MW956659 |
| MN801432 | MK458999 | MN797576 | MH632419 | KT378869 | KR823157 | KF267642 | KJ401683 | OR522347 | MW954909 |
| MN801412 | MK459076 | MN798724 | MH632312 | KT378881 | KT379173 | KM258806 | KJ401676 | OR522364 | MW954466 |
| MN801332 | MK458981 | MN797563 | MH573490 | KT378856 | KT379368 | KP418606 | KJ401661 | OR522348 | MW954397 |
| MN801275 | MK459253 | MN798025 | MH573489 | KU050303 | KT379327 | KJ401497 | KR188422 | OR522087 | MW954362 |
| MN801257 | MK459138 | MN797561 | MH573483 | KT378840 | KT379322 | KP250694 | KR188280 | OR521897 | MW954344 |
| MN801219 | MK459052 | MN798512 | MH573477 | KT378948 | KR823721 | KM258769 | KR188256 | OR521759 | MW954314 |
| MN801191 | MK458962 | MN799472 | MH573540 | KT378823 | KR823993 | KP418627 | KR188138 | OR521437 | MW952513 |
| MN801111 | MK458960 | MN798840 | MH573450 | KT379394 | KX791589 | KM258729 | KR188084 | OR521899 | MW954601 |
| MN801052 | MK459081 | MN797551 | MH573445 | KT379188 | KU954666 | KM258710 | KR188045 | OR521445 | MW954510 |
| MN800920 | MK459026 | MN797731 | MH573444 | KT378985 | KR823199 | KM258773 | KR188023 | OR522324 | MW954474 |
| MN800860 | MK458958 | MN797530 | MH573440 | KU050320 | KR824040 | KJ401595 | KR187992 | OR521954 | MW954289 |
| MN800567 | MK459146 | MN798615 | MH573425 | KT379644 | KR823592 | KJ401550 | KR187981 | OR522184 | MW954037 |
| MN800504 | MK459101 | MN797521 | MH573533 | KT379027 | KR823556 | KM258814 | KR187968 | OR522077 | MW952913 |
| MN800481 | MK458976 | MN798275 | MH573378 | KT378816 | KY226060 | KM258824 | KR187941 | OR521417 | MW955010 |
| MN800480 | MK459018 | MN797755 | MH573366 | KT379827 | KR823263 | KR822918 | KR187810 | MW957809 | MW954212 |
| MN800465 | MK458956 | MN797513 | MH573362 | KT379544 | KU050296 | KM258686 | KR187784 | OR521452 | MW953860 |
| MN800341 | MK459294 | MN798256 | MH573506 | KT379243 | KT379561 | KM395795 | KR187772 | OR521694 | MW953654 |
| MN800340 | MK459167 | MN797508 | MH573329 | KT379103 | KU954628 | KR605093 | KR187623 | OR521693 | MW955199 |
| MN800270 | MK459137 | MN797507 | MH573328 | KT379010 | KT379899 | KP235187 | KR187374 | OR522418 | MW955121 |
| MN800191 | MK459120 | MN797714 | MH573296 | KT378974 | KT379790 | KP235169 | KR187329 | OR522395 | MW954918 |
| MN800170 | MK459059 | MN797501 | MH573295 | KT378815 | KT379774 | KP235142 | KC888708 | OR522358 | MW954851 |
| MN800119 | MK459056 | MN799274 | MH573266 | KT378817 | KT379762 | KP235111 | KC888658 | OR522263 | MW954848 |
| MN800070 | MK459040 | MN797491 | MH573257 | KT378810 | KT379632 | KP235091 | KC352125 | OR522011 | MW954053 |
| MN799873 | MK458954 | MN797486 | MH573239 | KT379940 | KT379420 | KP235019 | KC352088 | OR521910 | MW954146 |
| MN801836 | MK459006 | MN798731 | MH573226 | KT378809 | KT379055 | KP235014 | KR106923 | OR521813 | MW954760 |
| MN801779 | MK458944 | MN797485 | MH573180 | KT378928 | KT379019 | KP234954 | KR106882 | OP190486 | MW954743 |
| MT590016 | MK459144 | MN798352 | MH632563 | KT378798 | KT378651 | KP234935 | KR106864 | OR521810 | MW954431 |
| MT588995 | MK459142 | MN799590 | MH573179 | KU050374 | KT378648 | KP234917 | KR106821 | MW957713 | MW957636 |
| MT589570 | MK458938 | MN799251 | MH573151 | KU050468 | KR824025 | KJ820399 | KR106800 | OK315236 | MW957531 |
| MT589573 | MN427036 | MN797460 | MH573149 | KT378776 | KR823552 | KJ820356 | KC352090 | OK315235 | MW957476 |
| MT589996 | MK287206 | MN798227 | MH300437 | KT379816 | KR823490 | KJ820325 | KC888478 | OR522307 | MW957376 |
| MN799911 | MN426684 | MN797452 | MH300429 | KT378762 | KR823414 | KJ820310 | KP992352 | OR521434 | MW957624 |
| MN801692 | MN426323 | MN798683 | MH300393 | KT379234 | KR823354 | KJ820245 | KR188431 | OP831092 | MW953529 |
| MN800204 | MK459275 | MN797656 | MH300392 | KT379205 | KR823240 | KJ820240 | KR188386 | OP190430 | MW953115 |
| MT589046 | MK458994 | MN797438 | MH300365 | KT379124 | KR823216 | KJ820182 | KR187915 | OP831044 | MW953023 |
| MT589392 | MN427346 | MN799289 | MH300343 | KT378750 | KR823205 | KJ820162 | KP992364 | OR521409 | MW953005 |
| MT590175 | MN427343 | MN797426 | MH300385 | KT378749 | KR823182 | KJ820145 | KJ401721 | OR521393 | MW951759 |
| MT589420 | MN427334 | MN799040 | MH300329 | KT379846 | KR823167 | KJ401554 | KJ401757 | OP191176 | MW951711 |
| MN802769 | MN427333 | MN797410 | MH300353 | KT378748 | KR823165 | KJ401415 | KJ401730 | OP831065 | MW956657 |
| MN801991 | MN427332 | MN798019 | MH300323 | KT378946 | KR823140 | KJ401568 | KR187320 | OR521955 | MW956512 |
| MN801102 | MN427330 | MN797408 | MH300321 | KT378751 | KU954745 | KJ614127 | KR187222 | OK315128 | MW956451 |
| MN800921 | MN427328 | MN799727 | MH300379 | KT378736 | KU954564 | KJ614005 | KC888221 | OR521877 | MW956437 |
| MN800803 | MN427326 | MN798555 | MH300307 | KT379328 | KU050563 | KJ401499 | KJ401748 | OR521761 | MW956375 |
